# Mechanism-based traps enable protease and hydrolase substrate discovery

**DOI:** 10.1038/s41586-022-04414-9

**Published:** 2022-02-16

**Authors:** Shan Tang, Adam T. Beattie, Lucie Kafkova, Gianluca Petris, Nicolas Huguenin-Dezot, Marc Fiedler, Matthew Freeman, Jason W. Chin

**Affiliations:** 1 Medical Research Council Laboratory of Molecular Biology, Cambridge, UK; 2 Sir William Dunn School of Pathology, University of Oxford, Oxford, UK

**Keywords:** Chemical tools, Enzymes

## Abstract

Hydrolase enzymes, including proteases, are encoded by 2–3% of the genes in the human genome and 14% of these enzymes are active drug targets^1^. However, the activities and substrate specificities of many proteases—especially those embedded in membranes—and other hydrolases remain unknown. Here we report a strategy for creating mechanism-based, light-activated protease and hydrolase substrate traps in complex mixtures and live mammalian cells. The traps capture substrates of hydrolases, which normally use a serine or cysteine nucleophile. Replacing the catalytic nucleophile with genetically encoded 2,3-diaminopropionic acid allows the first step reaction to form an acyl-enzyme intermediate in which a substrate fragment is covalently linked to the enzyme through a stable amide bond^2^; this enables stringent purification and identification of substrates. We identify new substrates for proteases, including an intramembrane mammalian rhomboid protease RHBDL4 (refs. ^3,4^). We demonstrate that RHBDL4 can shed luminal fragments of endoplasmic reticulum-resident type I transmembrane proteins to the extracellular space, as well as promoting non-canonical secretion of endogenous soluble endoplasmic reticulum-resident chaperones. We also discover that the putative serine hydrolase retinoblastoma binding protein 9 (ref. ^5^) is an aminopeptidase with a preference for removing aromatic amino acids in human cells. Our results exemplify a powerful paradigm for identifying the substrates and activities of hydrolase enzymes.

## Abstract (web summary)

Light-activated trapping is used in a mechanism-based assay to capture and identify substrates of protein hydrolases.

## Main

Activity-based probes have confirmed the presence and selective reactivity of the catalytic serine or cysteine nucleophile for many hydrolase proteins in cells^6–8^. Efforts to define hydrolase specificity have captured non-covalent interactors with hydrolases and investigated the substrates that accumulate in the absence of a hydrolase or the products that accumulate in the presence of a hydrolase^9^. Current approaches to identifying protease substrates mostly aim to either co-immunoprecipitate substrates that are non-covalently bound to catalytically inactive protease variants^10–12^, or identify the peptides resulting from the action of the protease from experiments with and without the protease^13–15^. In the first approach, substrates may be lost in the washing steps, and bound proteins may not be substrates. The second approach typically underestimates the number of substrates, and the cleavages identified may be indirect^16,17^. The identification of intramembrane protease substrates by current approaches is particularly challenging^18,19^. The methodological challenges in defining hydrolase and protease specificity mean that the substrates of many proteases remain unknown or incomplete, and many hydrolases remain orphans—with unknown substrates and uncharacterized specificity.

We previously demonstrated the genetically encoded, site-specific incorporation of photocaged Dap ((2*S*)-2-amino-3-{[(2-{[1-(6-nitrobenzo[*d*][1,3]dioxol-5-yl)ethyl] thio}ethoxy)carbonyl]amino}propanoic acid) (pc-Dap) into proteins expressed in *Escherichia coli*^2,20^. We converted pc-Dap to Dap (2,3-diaminopropionic acid) in proteins in vitro by illuminating purified proteins followed by incubation for up to 2 days at pH 8. By incubating purified proteases or thioesterases—in which we had replaced the catalytic cysteine or serine with Dap—with known substrates, we captured the otherwise transient thioester or ester intermediates—resulting from the first step of the reaction of these enzymes with their substrates—as their stable amide analogues. We demonstrated the utility of this approach for structural studies of acyl-enzyme intermediates^2^.

**Extended Data Fig. 1:**
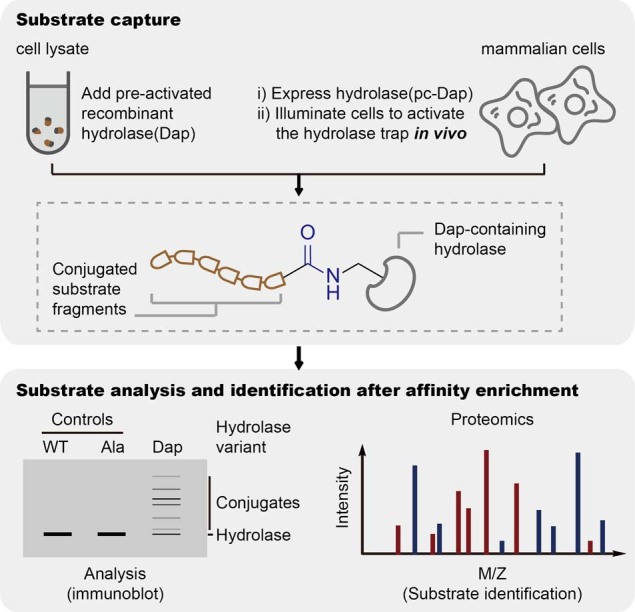
Strategies for Dap-mediated hydrolase substrate discovery. Genetically encoded pc-Dap in place of the catalytic serine or cysteine in the active site of a hydrolase enables the deprotection of pc-Dap to Dap and the activation of a hydrolase substrate trap that can covalently capture substrates. Purified recombinant and pre-activated Dap-containing hydrolase can be added to lysate or extract and used to capture substrates. Hydrolase containing pc-Dap can also be expressed directly in mammalian cells. The hydrolase substrate trap can then be optically activated in live cells to capture substrates. Covalent conjugates can be enriched by immunoprecipitation with stringent washing to remove non-covalent binders and the conjugates can be visualized and identified by immunoblot and mass spectrometry-based methods. Control experiments use the wild-type (WT) enzyme that does not form a stable covalent acyl-enzyme conjugate and the catalytically inactive mutant (Ala, catalytic serine/cysteine is mutated to alanine) of the hydrolase that does not react with substrates. We note that not all the Dap-containing hydrolase will necessarily be found in conjugates; in general, we expect the fraction of Dap-containing hydrolase in conjugates to be a function of hydrolase abundance, substrate abundance, effects on the rate of acyl-intermediate formation resulting from replacing the catalytic nucleophile with Dap, and the stability of the acyl-intermediate.

Here we demonstrate that genetically encoded pc-Dap coupled to mass spectrometry provides a powerful approach for discovering hydrolase substrates in complex mixtures and in live mammalian cells (Extended Data Fig. 1). We first demonstrate that recombinant proteases containing Dap in place of their catalytic nucleophile can be used to covalently link the N-terminal fragment of substrates to the protease, via a stable amide bond, in cell lysates. Through stringent purification of substrates linked to high-temperature requirement protein A2 (HtrA2) protease in combination with a mass spectrometry workflow we identify more than 200 new substrates for this protease. We then demonstrate that pc-Dap can be genetically encoded into proteins in mammalian cells. Illumination effects the rapid, post-translational conversion of pc-Dap to Dap in live cells and enables the specific covalent capture of the N-terminal fragments of protease substrates in cells. We identify new substrates for the mammalian rhomboid protease RHBDL4, an intramembrane protease that resides in the endoplasmic reticulum (ER). Our results demonstrate that RHBDL4 can shed luminal fragments of type I transmembrane proteins to the extracellular space. Upon removal of the ER retention motif, RHBDL4 promotes the secretion of a wide range of ER chaperones from cells. Finally, we develop a pipeline to directly identify the branched peptides formed between a substrate fragment and Dap and thereby reveal that the putative serine hydrolase retinoblastoma binding protein 9 (RBBP9) is an aminopeptidase with a preference for removing aromatic amino acids in mammalian cells.

## Trapping substrates in complex mixtures

**Extended Data Fig. 2:**
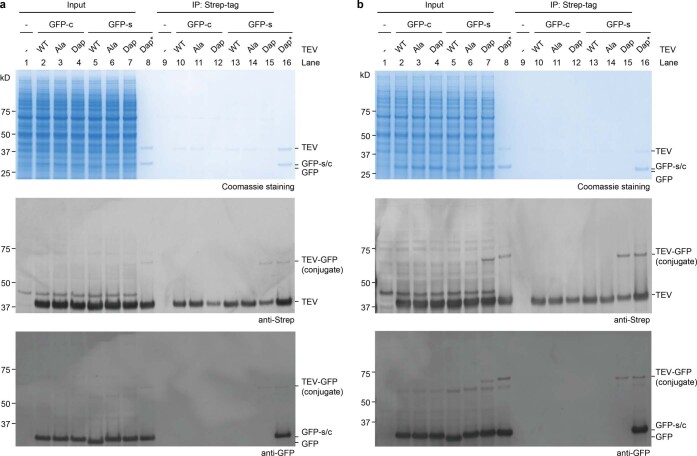
TEV(C151Dap) specifically traps its substrate in Expi293 cell lysate. TEV variants (WT, Ala, Dap, 1 μM) were incubated with control GFP-c (a polypeptide “Gly-Gly-Gly-Ser-Gly-Gly-Gly-His6” was attached at the C-terminus of GFP) or substrate GFP-s (a TEV cleavage sequence “Glu-Asn-Leu-Tyr-Phe-Gln-Gly-His6” was attached at the C-terminus of GFP. The cleavage between Gln and Gly is underlined) in Expi293 cell lysate at 37 °C for 3 h. The concentration of GFP-s or GFP-c was (**a**) 1 μM or (**b**) 5 μM. Dap^*^: reaction of TEV(C151Dap) with GFP-s in Tris buffer. The input (reaction before IP) and Strep-tag enriched TEV species were analyzed by SDS-PAGE (Coomassie staining) and WB (anti-Strep for TEV and anti-GFP). (**a**) and (**b**) were repeated in three biological replicates with similar results. For gel source data, see Supplementary Fig. 1.

**Extended Data Fig. 3:**
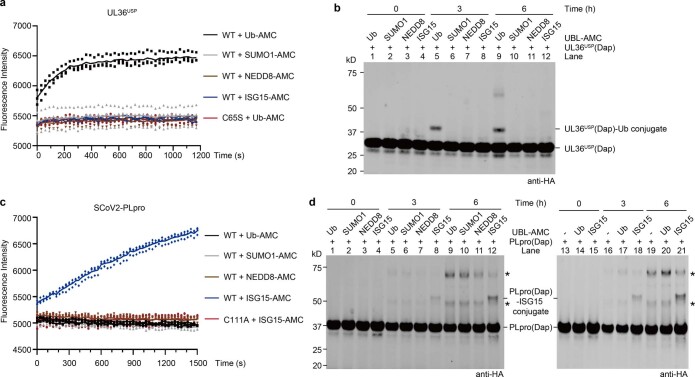
Dap-containing proteases selectively capture known substrates of their parent enzymes. (**a**) The change of AMC fluorescence resulting from cleavage from the C-terminus of Ubiquitin (Ub) or ubiquitin-like molecules (SUMO, NEDD8, ISG15) was followed upon mixing with UL36^USP^. WT UL36^USP^ from human herpesvirus 1 specifically hydrolyses Ub-AMC, but not other ubiquitin-like protein-AMC (UBL-AMC) molecules. The deubiquitination activity of UL36^USP^ is lost when Cys65 is mutated to Ser. This data is consistent with the previously reported specificity of UL36^USP^^58^. (**b**) UL36^USP^(C65Dap) specifically reacts with Ub-AMC to form the UL36^USP^(Dap)-Ub conjugate. In contrast, no conjugates were observed between UL36^USP^(Dap) and SUMO1, NEDD8 or ISG15. (**c**) WT SCoV2-PLpro selectively hydrolyses ISG15-AMC in preference to other Ub/UBL-AMC molecules; this data is consistent with the previously reported specificity of SCoV2-PLpro^59^. The hydrolysis of ISG15-AMC is abrogated when the catalytic Cys111 of SCoV2-PLpro is mutated to Ala. (d) SCoV2-PLpro(C111Dap) specifically reacts with ISG15-AMC, generating the SCoV2-PLpro(Dap)-ISG15 conjugate. *: UBL-AMC independent higher MW bands resulting from PLpro(C111Dap) self-reaction. (**a**) and (**c**) were generated using n = 3 independent measurements. The line represents the means of three measurements. (**b**) was repeated twice and (**d**) was performed in three biological replicates with similar results. For gel source data, see Supplementary Fig. 1. Source data

We demonstrated that recombinant Tobacco etch virus (TEV) protease containing Dap in place of its catalytic cysteine (TEV(C151Dap)) formed a specific conjugate with GFP with a TEV protease cleavage site appended at its C-terminus (GFP-s) when the two proteins were incubated together in human cell lysate (Extended Data Fig. 2). Although GFP-s was present in the lysate at comparable levels to many other proteins, TEV(C151Dap) formed a specific conjugate, observed by immunoblotting, with its substrate partner and not with any other proteins in the lysate. Control experiments demonstrated that conjugate formation was dependent on both the presence of Dap in the protease and the presence of the TEV cleavage site in the substrate. Replacing the catalytic cysteine of other proteases (UL36^USP^ and SCoV2-PLpro) with Dap also led to the selective capture of their known substrates (Extended Data Fig. 3). We conclude that replacing the active site nucleophile with Dap allows the specific covalent capture of substrates.

## Profiling HtrA2 substrates

Next, we demonstrated the utility of Dap-containing proteases for the capture and identification of protease substrates from cell lysate. We focussed on the mature cytosolic form of HtrA2, which is released from the mitochondrial intermembrane space and relocated to the cytosol upon stress^21,22^. Several substrates have been identified for cytosolic HtrA2, including caspase inhibitors^21^ (for example, XIAP and cIAP1/2). HtrA2 contains a PDZ domain and chymotrypsin-like protease domain; since PDZ domains bind a wide range of proteins and chymotrypsin-like domains commonly have broad specificity^23^, we hypothesized that there may be undiscovered substrates of HtrA2.

**Fig. 1:**
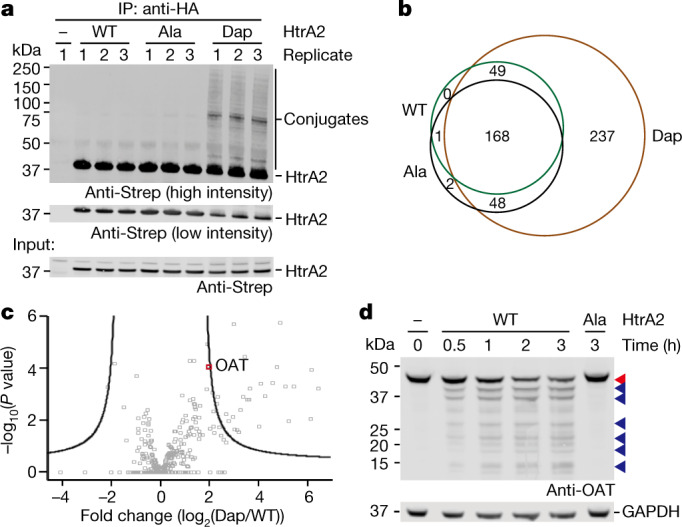
Dap-mediated HtrA2 substrate identification in mammalian cell lysate. **a**, HtrA2(S306Dap)–HA–Strep and its conjugates were enriched from cell lysate with anti-HA beads and detected with an anti-Strep antibody. Control experiments were performed with wild-type (WT) HtrA2 and the catalytically inactive S306A mutant. Input: HtrA2 variants in cell lysates before incubation. **b**, Venn diagram showing the number of proteins identified in HtrA2(S306Dap) elution compared with controls. Proteins identified in at least two of the three replicates were considered as positively identified. **c**, Volcano plot based on label-free quantification (LFQ) values for the proteins identified in HtrA2(S306Dap) and wild-type HtrA2 samples. The black line represents the cut-off curve for significance (*S*_0_ = 1, FDR < 0.01). Each data point is calculated in Perseus using *n* = 4 for each HtrA2 variant. The dot representing ornithine aminotransferase (OAT) is labelled in red. **d**, Wild-type HtrA2 or HtrA2(S306A) (1 μM) was added into Expi293 cell lysate and incubated for the indicated time at 37 °C. Red arrowhead, full-length OAT; blue arrowhead, wild-type HtrA2-dependent proteolytic fragments. GAPDH was used as a loading control. The experiment in **a** was performed in biological triplicate, and the experiment in **d** was performed in two biological replicates, both with similar results. For gel source data, see Supplementary Fig. 1.

We replaced the catalytic serine of HtrA2 with Dap, creating HtrA2(S306Dap)–HA–Strep (Supplementary Fig. 2). We incubated this protein with human cell lysate and enriched covalent conjugates to the protease by immunoprecipitation followed by stringent washing. The conjugates were eluted and immunoblotting against the Strep-tag on Dap-containing HtrA2 revealed numerous species with a higher apparent molecular mass (Fig. 1a). These species were not observed in the control experiments and are primarily covalent conjugates to Dap-containing HtrA2.

Proteomic analysis identified 274 proteins that were specifically captured by HtrA2(S306Dap); 237 of these proteins were not found in control experiments (Fig. 1b, Supplementary Data File 1) and 37 proteins were significantly enriched (minimum fold change (*S*_0_) = 1, false discovery rate (FDR) < 0.01) in the HtrA2(S306Dap) elution with respect to controls (Fig. 1c, Supplementary Fig. 2). Our study identified 17 times more potential HtrA2 substrates than previous work, and this approach identified the majority of the proteins previously identified as HtrA2 substrates by N-terminal proteomics^24^ (Supplementary Table 1).

We tested 29 of the newly identified potential substrates for proteolysis with wild-type HtrA2. Eighty-six percent of the candidates showed a decrease in the abundance of full-length protein or the generation of defined proteolytic fragments over time upon addition of the wild-type protease (Fig. 1d, Supplementary Fig. 3, Supplementary Tables 2, 3). Overall, our results demonstrate that we have developed an approach for the efficient and specific identification of protease substrates in complex mixtures.

**Fig. 2:**
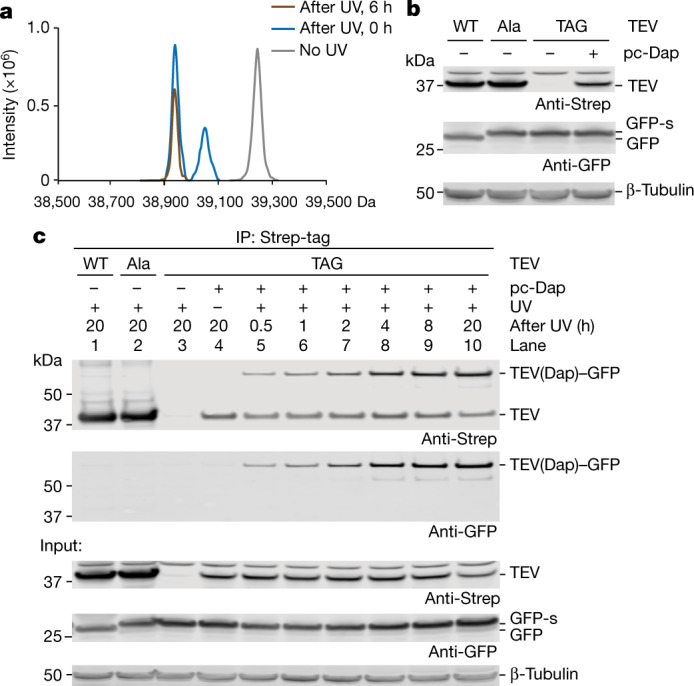
Optical activation of a Protease(Dap) substrate trap in human cells. **a**, The mass of TEV(C151pc-Dap) before and after illumination of human cells expressing the protein. TEV containing pc-Dap was produced using the DapRS–tRNA_CUA_ pair and a TEV gene bearing the amber codon (TAG) at position 151 in the presence of pc-Dap. The grey trace shows proteins purified before illumination. Fully protected TEV(C151pc-Dap) bearing an acetyl group ([Ac-TEV(C151pc-Dap)]: expected 39,245.4 Da, observed 39,244.4 Da). The blue trace shows proteins purified immediately after illumination of cells. The fully deprotected product ([Ac-TEV(C151Dap)]: expected 38,948.2 Da, observed 38,945.2 Da); the deprotection intermediate ([Ac-TEV(C151Dap_inter_)]: expected 39,052.2 Da, observed 39,054.0 Da). The brown trace shows proteins purified 6 h after illumination of cells. **b**, TEV variants and GFP-s were co-expressed in HEK293T cells for 48 h. Total lysate was analysed by anti-Strep (for TEV) and anti-GFP antibodies. Wild-type TEV quantitatively cleaved GFP-s to GFP. β-Tubulin was used as a loading control. **c**, Detection of TEV(Dap)–GFP conjugate after Strep-tag enrichment. Samples, not illuminated (lane 4) or after UV illumination (lanes 1–3 and 5–10) were collected at indicated time points. Input, cell lysates before immunoprecipitation probed with anti-Strep and anti-GFP antibodies. β-Tubulin was used as a loading control. Experiments in **a**–**c** were performed in two biological replicates with similar results. For gel source data, see Supplementary Fig. 1.

## Trapping substrates in live human cells

**Extended Data Fig. 4:**
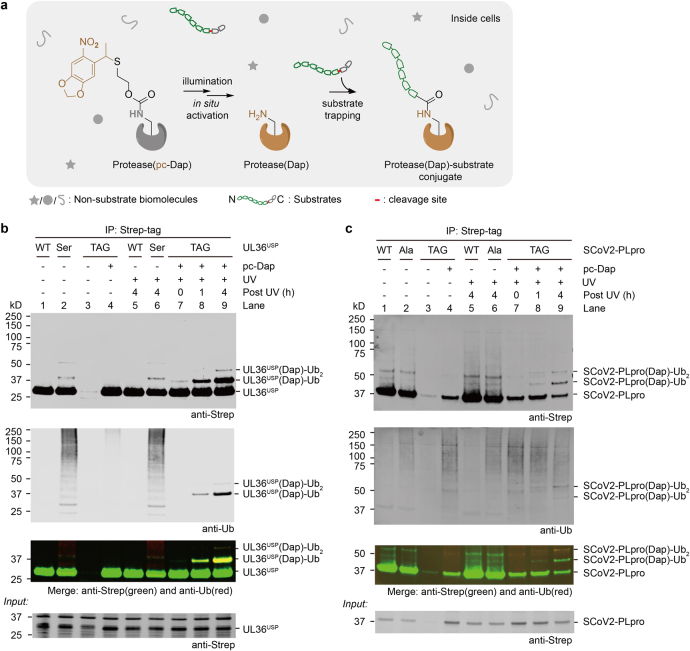
UL36^USP^(C65Dap) and SCoV2-PLpro(C111Dap) form conjugates with endogenous ubiquitin in live cells. (**a**) A protease containing pc-Dap in place of its catalytic serine or cysteine is produced in mammalian cells by genetic code expansion, creating a photocaged protease trap. The trap is activated by illuminating cells. The activated trap covalently and specifically captures substrate fragments in acyl-enzyme complexes linked through stable amide bonds. (**b**) Immunoblotting analysis of UL36^USP^ variants enriched from an equal number of cells after optical activation and substrate capture. The conjugates formed between UL36^USP^(C65Dap) and endogenous proteins were detected by an anti-Strep antibody and an anti-Ub antibody; this demonstrates that UL36^USP^(C65Dap) captures Ub and di-ubiquitin (Ub_2_) in cells. Catalytically inactive UL36^USP^(C65S) non-covalently associated with Ub chains to co-IP them (lanes 2 and 6), while UL36^USP^(C65Dap) formed conjugates with endogenous Ub molecules (lanes 7–9). Input: cell lysates before IP probed with an anti-Strep antibody. These data are consistent with previous work demonstrating that UL36^USP^ is a deubiquitinase in cells^58^. (**c**) Immunoblotting analysis of SCoV2-PLpro(C111Dap) variants enriched from an equal number of cells after optical activation and substrate capture. The conjugates formed between SCoV2-PLpro(C111Dap) and endogenous proteins were detected by an anti-Strep antibody and an anti-Ub antibody; this demonstrates that SCoV2-PLpro(C111Dap) captures Ub and Ub_2_ in cells. Catalytically inactive SCoV2-PLpro(C111A) and SCoV2-PLpro(C111pc-Dap) without illumination non-covalently associated with Ub chains to co-IP them (lanes 2, 4 and 6), while SCoV2-PLpro(C111Dap) also formed conjugates with endogenous Ub molecules (lanes 8 and 9). Input: cell lysates before IP probed with an anti-Strep antibody. Note that SCoV2-PLpro has previously been shown to cleave poly-Ub chains in cells^59,60^. While SCoV2-PLpro will also cleave ISG15, this protein is not expressed in Expi293 cells without stimulation^61,62^. (**b**) and (**c**) were repeated in two biological replicates with similar results. For gel source data, see Supplementary Fig. 1.

Next, we set out to extend our approach to capturing and identifying protease substrates in human cells (Extended Data Fig. 4a). It was unclear—on the basis of previous work^2^—whether pc-Dap could be converted to Dap in live cells to activate protease substrate traps. We set out to: encode pc-Dap in human cells, investigate the activation of a protease substrate trap through deprotection of pc-Dap to Dap in human cells, and demonstrate the covalent capture of protease substrates in human cells.

We demonstrated the efficient, site-specific, genetically directed incorporation of pc-Dap into proteins in human cells (Supplementary Figs. 4, 5). We produced TEV protease in which the catalytic cysteine was replaced by pc-Dap (TEV(C151pc-Dap)) in human cells (Supplementary Fig. 6). To deprotect pc-Dap we illuminated cells^25,26^ expressing TEV(C151pc-Dap) for 2 min. Mass spectrometry revealed that more than 60% of the TEV purified from cells immediately after illumination was fully deprotected to TEV(C151Dap); the remaining 40% of TEV contained the deprotection intermediate. Six hours after illumination, we detected only TEV(C151Dap) (Fig. 2a); this demonstrated that deprotection, from pc-Dap to Dap—to activate the protease substrate trap—proceeds rapidly in live cells.

To demonstrate that we can capture protease substrates in human cells, we co-expressed TEV(C151pc-Dap) and GFP-s (Fig. 2b). We illuminated HEK293T cells to generate TEV(C151Dap) and followed the formation of the TEV(Dap)–GFP conjugate in cells by immunoblotting (Fig. 2c, Supplementary Fig. 7). The conjugate was observed 30 min after illumination of cells and accumulated over time. We did not detect the conjugate by immunoblotting from cells expressing wild-type TEV, TEV(C151A) or cells expressing TEV(C151pc-Dap) before illumination. No other conjugates were observed, further confirming the specificity of the approach. Tandem mass spectrometry (MS/MS) explicitly demonstrated the formation of an amide bond between TEV(C151Dap) and GFP in cells (Supplementary Fig. 8). Additional experiments confirmed that replacing the catalytic residue with Dap in other cysteine proteases (UL36^USP^ and SCoV2-PLpro) led to the covalent capture of their known substrates in human cells (Extended Data Fig. 4b, c). Overall, these experiments demonstrated that: pc-Dap can be site-specifically incorporated into soluble proteases in human cells, protease substrate traps can be rapidly activated by illumination of human cells, and activated protease substrate traps can be used to specifically and covalently capture protease substrates in human cells.

## Trapping a model substrate of RHBDL4

Intramembrane proteases have diverse and important roles in biological regulation^27,28^. However, defining the physiological substrates of intramembrane proteases in mammalian cells has proved exceptionally challenging^18,29,30^. Rhomboid proteases are an important class of intramembrane protease that use a catalytic serine for catalysis^3,31,32^. We focused on RHBDL4, a rhomboid protease located in the ER membrane of mammalian cells, which has been associated with multiple cellular pathways^4,12,33–35^, and potentially Alzheimer disease pathology^36^. However, physiologically relevant substrates of RHBDL4 remain essentially unknown. We set out to: encode pc-Dap in place of the catalytic serine in RHBDL4, activate the rhomboid protease substrate trap by illuminating cells, and demonstrate the covalent capture and identification of substrates for RHBDL4.

**Extended Data Fig. 5:**
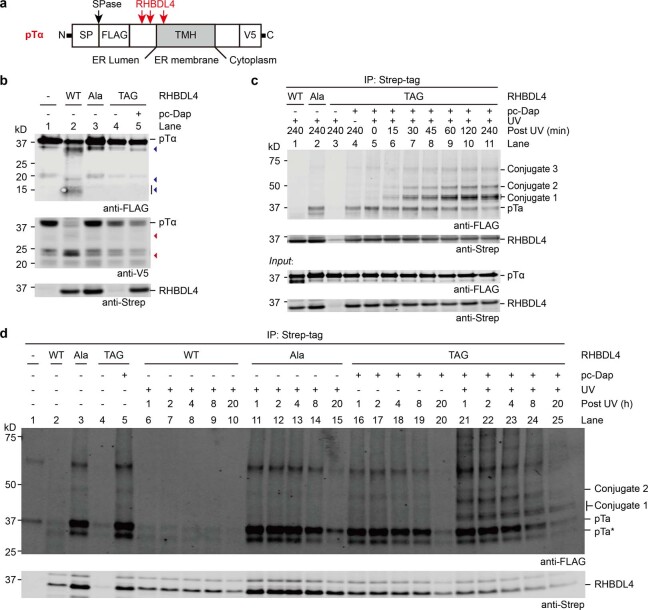
Formation of RHBDL4(Dap)-pTα conjugates in Expi293 cells. (**a**) Schematic representation of the model substrate pTα. SP: signal peptide; FLAG: FLAG-tag; TMH: transmembrane helix; V5: V5-tag. Red arrows: RHBDL4 cleavage sites; black arrow: SPase cleavage site. (**b**) Co-expression of C-terminal Strep-tagged RHBDL4 variants and pTα in Expi293 cells for 40 h. pc-Dap was added to produce full-length RHBDL4(S144pc-Dap). Cell lysates were analysed by anti-Strep (for RHBDL4), anti-FLAG and anti-V5 antibodies. Blue triangles: N-terminal proteolytic fragments of pTα; Red triangles: C-terminal proteolytic fragments of pTα. (**c**) Detection of RHBDL4(Dap)-pTα conjugates after Strep-tag enrichment. Samples without illumination (lane 4) and with illumination (lanes 1–3 and 5–11) were collected at indicated time points. Input: cell lysate before IP analysed by anti-Strep and anti-FLAG antibodies. (**d**) The formation of conjugates was monitored for a longer period of time. RHBDL4(Dap)-pTα conjugates were gradually degraded 4 h after UV irradiation. RHBDL4 variants were also gradually degraded over time pTα*, the deglycosylated form of pTα. The experiments were repeated in two biological replicates with similar results. For gel source data, see Supplementary Fig. 1.

We produced RHBDL4(S144pc-Dap) at a similar level to a wild-type control, and confirmed that the protein localized predominantly in the ER membrane (Supplementary Fig. 9), as expected^4,35^. We co-expressed a model substrate pTα, which is efficiently cleaved by wild-type RHBDL4 at multiple sites^4^ (Extended Data Fig. 5a, b). We illuminated cells containing RHBDL4(S144pc-Dap) and pTα to activate the protease substrate trap and followed conjugate formation by immunoblot against the N-terminal Flag tag on pTα after affinity purification using the twin-Strep-tag on RHBDL4 (Extended Data Fig. 5c). We detected Dap-specific conjugates 15 min after illumination of cells expressing RHBDL4(S144pc-Dap); these conjugates rapidly accumulated within 4 h (Extended Data Fig. 5d, Supplementary Fig. 10). Overall, these experiments demonstrated that we can express and optically activate a protease substrate trap for RHBDL4, and that the trap efficiently captures its model substrate.

## Identifying RHBDL4 substrates

**Fig. 3:**
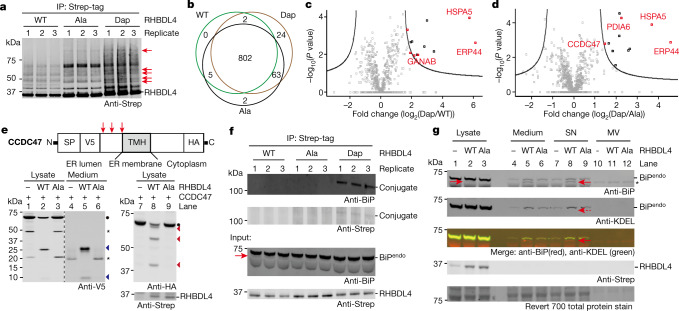
Discovery of RHBDL4 substrates. **a**, RHBDL4 variants were expressed in Expi293 cells. Immunoblotting analysis of RHBDL4 variants enriched from an equal number of cells after optical activation and substrate capture. Ala, RHBDL4(S144A); Dap, RHBDL4(S144Dap). Red arrows, RHBDL4(S144Dap)-specific higher molecular mass bands. **b**, Venn diagram showing the number of proteins identified in RHBDL4(S144Dap) elution with respect to controls. Proteins found in at least two of the three replicates were considered to be positively identified. **c**, **d**, Volcano plots based on the LFQ values for the identified proteins for RHBDL4(S144Dap) versus wild-type RHBDL4 (**c**; *S*_0_ = 1, FDR < 0.01) and RHBDL4(S144Dap) versus RHBDL4(S144A) (**d**; *S*_0_ = 1.5, FDR < 0.05). Black lines represent the cut-off curve for significance. Each point was calculated in Perseus using *n* = 3 for each RHBDL4 variant. ER-resident candidates are marked in red and select candidates are also labelled. **e**, RHBDL4 cleaves CCDC47. SP, signal peptide; V5, V5-tag; TMH, transmembrane helix; HA, HA-tag; red arrows, RHBDL4 cleavage sites; black circles, full-length CCDC47; blue triangles, N-terminal proteolytic fragments; red triangles, C-terminal proteolytic fragments; asterisk, bands present without RHBDL4. **f**, RHBDL4(S144Dap) conjugates to endogenous BiP and the cleavage of endogenous BiP by wild-type RHBDL4. Cell lysates before immunoprecipitation (input) and the conjugates were directly visualized by anti-BiP and anti-Strep antibodies after Strep-tag enrichment. Red arrow, the proteolytic fragment of endogenous BiP cleaved by wild-type RHBDL4. **g**, Cleaved BiP is secreted into the medium. The medium was separated into supernatant (SN) and microvesicles (MV). Red arrow, the proteolytic fragment. Whereas full-length BiP was detected by both anti-BiP and anti-KDEL antibodies, cleaved BiP can only be detected with an anti-BiP antibody. Asterisk indicates non-specific bands. Revert 700 total protein stain was used as a loading control. Experiments in **a**, **f**, were performed in biological triplicate; the experiment in **e** was repeated in three biological replicates; the experiment in **g** was repeated in two biological replicates; all with similar results. For gel source data, see Supplementary Fig. 1.

To identify RHBDL4 substrates, we illuminated human cells producing RHBDL4(S144pc-Dap). We collected the membrane fraction and purified the protease conjugates by Strep-tag affinity enrichment. Anti-Strep analysis of the affinity-enriched samples showed several bands of higher apparent molecular mass in the RHBDL4(S144Dap) experiments that were not observed in control experiments (Fig. 3a); this suggested that the Dap-containing protease had covalently captured RHBDL4 substrates.

Proteomic analysis identified 43 potential RHBDL4 substrates; 24 of these proteins were detected only in the RHBDL4(S144Dap) elution (Fig. 3b) and 19 of them were significantly enriched in the RHBDL4(S144Dap) samples relative to controls (Fig. 3c, d). We note that proteins enriched or uniquely identified in both the RHBDL4(S144Dap) and RHBDL4(S144A) samples with respect to the wild-type control may also be substrates (Supplementary Data File 2), but these were not investigated in our subsequent analysis. Twenty-five of the candidate substrates are ER-associated proteins, including 15 ER-resident proteins (5 of which are transmembrane proteins), 2 nuclear transmembrane proteins, and 8 proteins in secretory pathways (Supplementary Table 4). We also identified non-ER-associated proteins, primarily nuclear proteins (Supplementary Fig. 11, Supplementary Note 1). We investigated the RHBDL4-mediated cleavage of a subset of the ER-associated candidate substrates.

**Extended Data Fig. 6:**
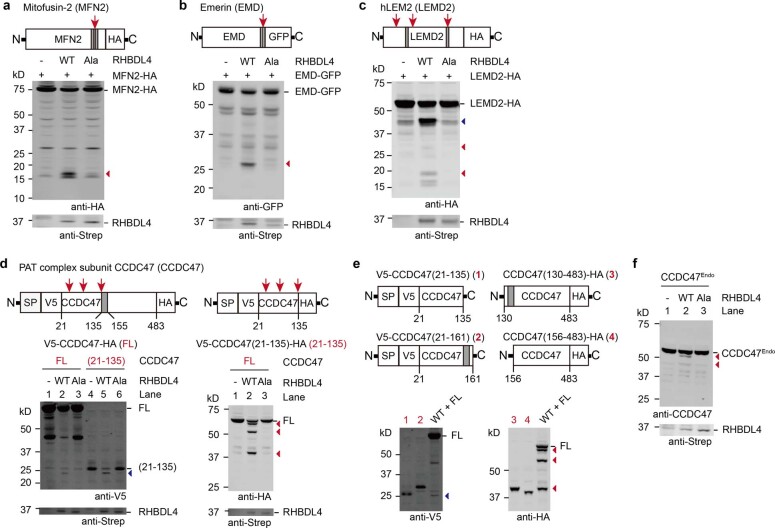
RHBDL4 cleaves transmembrane substrate candidates. RHBDL4 cleavage assays for (**a**) MFN2, (**b**) EMD, (**c**) LEMD2 and (**d**) CCDC47. The putative cleavage sites – the approximate positions of which were estimated based on the MW of the proteolytic bands – are indicated by red arrows in schematic representations. HA: HA-tag; GFP: GFP-tag; SP: signal peptide; V5: V5-tag. The transmembrane helices are labeled in grey. (**a**) WT RHBDL4 cleaved MFN2, a multiple membrane-spanning protein resident in mitochondria or the ER. Red triangle: the C-terminal proteolytic fragment. (**b**) WT RHBDL4 cleaved EMD, a type II transmembrane protein in the nuclear inner membrane. Red triangle: the C-terminal proteolytic fragment. (**c**) WT RHBDL4 cleaved LEMD2, a multiple membrane-spanning protein in the nuclear inner membrane. Two minor cleavages (red triangles) would be in the perinuclear space, but the major cleavage site (blue triangle) in the domain resident in nucleus might be cut by the inverted RHBDL4 (see Supplementary Note 1). (**d**) RHBDL4 cleaved both full-length (FL) CCDC47 and CCDC47(21–135), generating the same MW N-terminal proteolytic fragment (lanes 2 and 5, blue triangle); this suggested that RHBDL4-mediated cleavage is independent of the transmembrane helix of CCDC47. Blue triangle: the N-terminal fragment; Red triangles: C-terminal proteolytic fragments. (**e**) Comparison of the proteolytic fragments to truncated CCDC47 standards confirmed that the cleavages are in the luminal domain of CCDC47. WT + FL: FL CCDC47 cleaved by WT RHBDL4. (**f**) Over-expressed WT RHBDL4 cleaved endogenous CCDC47. Red triangles: proteolytic fragments detected by an anti-CCDC47 antibody. The C-terminal proteolytic fragments of MFN2, LEMD2 and CCDC47 were probed with an anti-HA antibody, while the proteolytic fragment of EMD was detected by an anti-GFP antibody. The N-terminal proteolytic fragments were probed with an anti-V5 antibody. RHBDL4 variants were detected by an anti-Strep antibody. (**a**-**d**) were repeated in three biological replicates with similar results. (**e**) was performed once. (**f**) was repeated in two biological replicates with similar results.  For gel source data, see Supplementary Fig. 1.

We first validated transmembrane protein candidates—the protein class that is conventionally considered as rhomboid protease substrates—using a cell-based rhomboid gain-of-function cleavage assay^4^. Over-expressed wild-type RHBDL4, but not the catalytically inactive S144A mutant, cleaved all tested transmembrane proteins identified by Dap conjugation (Fig. 3e, Extended Data Fig. 6a–c). RHBDL4 cleaved CCDC47 (ref. ^37^) at three positions in the luminal domain, and the ectodomains resulting from proteolysis were secreted into extracellular media (Fig. 3e and Extended Data Fig. 6d, e**)**. In addition, endogenous CCDC47 was cleaved by exogenously expressed wild-type RHBDL4 (Extended Data Fig. 6f). Our data collectively demonstrated that RHBDL4 can proteolyze the transmembrane proteins identified by our approach and, upon cleavage by RHBDL4, luminal fragments of CCDC47 are released into the extracellular medium.

Soluble proteins have not been reported as endogenous RHBDL4 substrates. However, a substantial percentage (67%) of the ER-resident proteins that we identified are soluble; the majority of these proteins (90%) are chaperones (Supplementary Table 4). We therefore focussed on further investigating the cleavage of ER soluble chaperones by RHBDL4.

**Extended Data Fig. 7:**
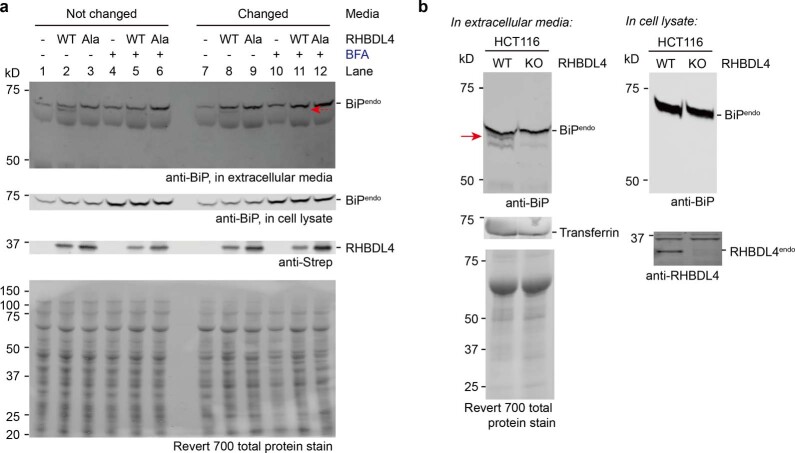
RHBDL4 cleaves endogenous BiP in mammalian cells and facilitates the secretion of N-terminal proteolytic fragments. (**a**) BFA inhibitory assay was performed for endogenous BiP. The secretion of BiP proteolytic fragment into extracellular media was inhibited by Brefeldin A (BFA) compared to cells treated with DMSO (lanes 5 vs 2, 11 vs 8, anti-BiP). Especially when the medium was changed, no BiP proteolytic fragment was detected in the medium (lane 11, dashed red arrow). Treatment of BFA greatly increased the endogenous level of BiP (lanes 4–6 vs 1–3, anti-BiP in cell lysate). In comparison, the expression of endogenous BiP was slightly increased when WT RHBDL4 or RHBDL4(S144A) was expressed (lanes 2 and 3 vs 1, anti-BiP in cell lysate). BFA treatment or RHBDL4 variant expression facilitated the secretion of full-length BiP (lane 4 vs 1 or 2 vs 1), but not the proteolytic fragment generated by RHBDL4 cleavage. RHBDL4 expression was detected by an anti-Strep antibody in cell lysate. Revert 700 total protein staining was used as loading control. (**b**) Endogenous BiP was cleaved by endogenously expressed RHBDL4. WT RHBDL4-dependent BiP proteolytic fragment in the media was detected by an anti-BiP antibody in WT HCT116 cells (red arrow), but not in RHBDL4 knockout HCT116 cells. Transferrin: loading control; additionally, the same samples run in different lanes were stained by Revert 700 for total protein loading control. Endogenous BiP was detected by an anti-BiP antibody and RHBDL4 was detected by an anti-RHBDL4 antibody in the cell lysate. All experiments were repeated in two biological replicates with similar results. For gel source data, see Supplementary Fig. 1.

We identified binding immunoglobulin protein (BiP) (encoded by *HSPA5*) as a potential substrate using our approach (Fig. 3c, d). We directly visualized the conjugates of RHBDL4(Dap) and endogenous BiP in human cells (Fig. 3f, Supplementary Fig. 12). The RHBDL4 cleavage assay confirmed that BiP can be cleaved at its interdomain linker region and C-terminus by wild-type RHBDL4. The N-terminal fragments generated by these cleavages—which do not possess the ER retention motif^38^ (KDEL sequence from the C-terminus of BiP)—were secreted into the medium (Fig. 3g and Supplementary Fig. 13). Endogenous BiP was cleaved by exogenously expressed WT RHBDL4, and secretion of the proteolytic fragment into the medium was Brefeldin A (BFA) sensitive (Extended Data Fig. 7a), consistent with secretion by the conventional pathway. Importantly, the proteolytic fragment from endogenous BiP was detected in the extracellular medium from WT cells, but not from an RHBDL4 knockout cells (Extended Data Fig. 7b), confirming the cleavage of endogenous BiP by endogenous RHBDL4. Collectively, our data demonstrate that a fraction of the soluble BiP present in cells is a physiological substrate of endogenous RHBDL4.

**Extended Data Fig. 8:**
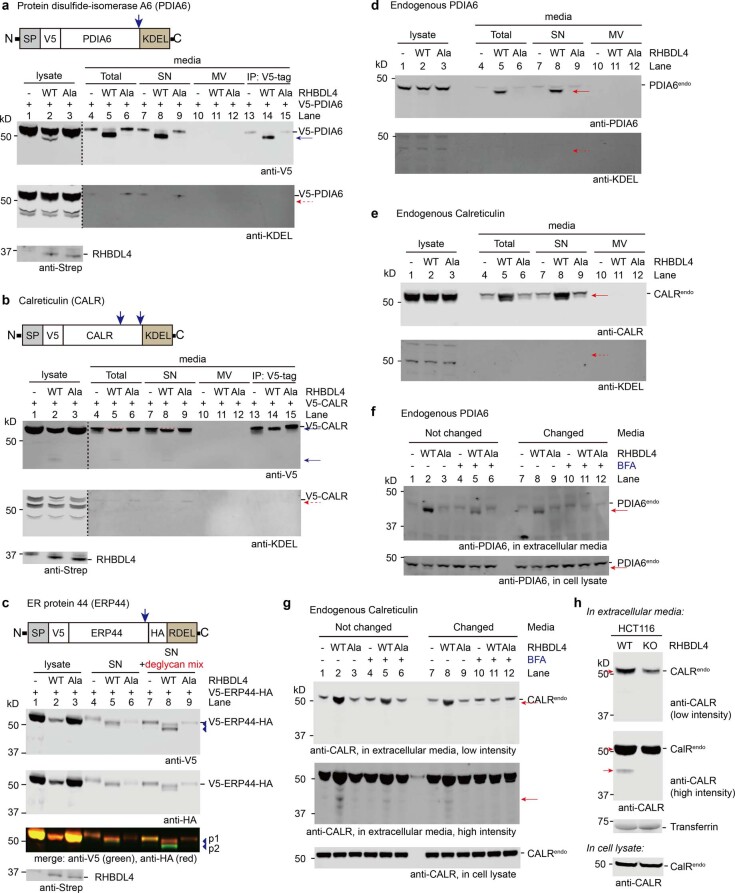
RHBDL4 cleaves other ER-resident proteins before ER retention motifs and facilitates the secretion of N-terminal proteolytic fragments. RHBDL4 cleavage assays for V5-tagged (**a**) PDIA6, (**b**) Calreticulin and (**c**) ERP44. The putative cleavage sites are indicated by blue arrows in the schematic representations. (**a**) WT RHBDL4 cleaved PDIA6 at the C-terminus, resulting in secretion of the N-terminal fragment (blue arrow) into the medium. (**b**) WT RHBDL4 cleaved Calreticulin at the internal region (minor cleavage) and the C-terminus (major cleavage), resulting in secretion of the N-terminal fragments (blue arrows) into the SN. (**c**) WT RHBDL4 cleaved ERP44 before RDEL sequence. The secreted proteins were deglycosylation mix II sensitive. Because we could not source an antibody to specifically detect the RDEL sequence, we inserted an HA-tag four amino acids before the RDEL sequence for detection. Anti-V5 detected proteolytic fragments 1 and 2 (p1 and p2), and p1 was also detected by the anti-HA antibody, indicating that one cleavage might happen after the HA-tag. The expression of RHBDL4 was detected by an anti-Strep antibody. RHBDL4 cleavage assays for (**d**) endogenous PDIA6 and (**e**) endogenous Calreticulin. (**d**) WT RHBDL4 cleaved endogenous PDIA6. The proteolytic fragment of PDIA6 (red arrow) without the KDEL sequence was secreted into the SN. (**e**) WT RHBDL4 cleaved endogenous Calreticulin. The proteolytic fragment of Calreticulin (red arrow) without the KDEL sequence was secreted into the SN. Expression of RHBDL4 did not increase the endogenous level of PDIA6 and Calreticulin (lanes 2 and 3 vs 1). The expression of RHBDL4 is shown in Fig. 3g. BFA inhibitory assays for (**f**) PDIA6 and (**g**) Calreticulin. The secretion of proteolytic fragments of PDIA6 and Calreticulin was inhibited compared to cells treated with DMSO (lanes 5 vs 2, 11 vs 8). Endogenous PDIA6 and Calreticulin were detected by an anti-PDIA6 and an anti-CALR antibody, respectively. The expression of RHBDL4 is shown in Extended Data Fig. 7a. (**h**) Endogenous RHBDL4 cleaves endogenous Calreticulin. WT RHBDL4-dependent Calreticulin proteolytic fragments in the extracellular media were detected by an anti-CALR antibody in WT HCT116 cells (red arrows), but not in RHBDL4 KO HCT116 cells. The secretion of the major proteolytic fragment (near full-length Calreticulin, low intensity) and the minor proteolytic fragment (high intensity) were both detected. Transferrin: loading control. The endogenous RHBDL4 is shown in Extended Data Fig. 7b. (**a**-**h**) were repeated in two biological replicates with similar results. For gel source data, see Supplementary Fig. 1.

RHBDL4-mediated removal of the C-terminal ER retention motif from soluble ER-resident protein candidates was also validated for protein disulfide-isomerases (protein disulfide-isomerase A6 (gene name: *PDIA6)* and ER protein 44 (gene name: *ERP44*) and calcium-binding chaperone Calreticulin (gene name: *CALR*), which were identified as potential substrates through our approach (Extended Data Fig. 8a–e). The secretion of proteolytic fragments of endogenous PDIA6 and Calreticulin was BFA sensitive (Extended Data Fig. 8f, g). The cleavage of a fraction of endogenous Calreticulin by endogenous RHBDL4 was detected in extracellular medium by an anti-Calreticulin antibody in wild-type but not in RHBDL4 knockout cells (Extended Data Fig. 8h).

Additional tests confirmed that RHBDL4 also cleaved other ER-resident soluble chaperones which were identified through our approach, including Calumenin, peptidyl-prolyl cis-trans isomerase FKBP9, Glucosidase 2 subunit α and β; in each case the cleavage led to secretion of the resulting N-terminal fragments into extracellular media (Supplementary Fig. 14). However, RHBDL4 did not cleave Calnexin, an abundant ER-resident chaperone, which was not identified as a potential substrate by our approach (Supplementary Figs. 12 and 14); this observation is consistent with our approach specifically capturing RHBDL4 substrates.

Collectively, our results demonstrate that we can capture and identify substrates for an intramembrane protease. Moreover, we have discovered that RHBDL4 can act as a non-canonical secretase. Unlike conventional secretases—which cleave transmembrane substrates in transmembrane domains or juxtamembrane domains to release ectodomains^16,18^—cleavage by RHBDL4 has the effect of removing the C-terminal ER-retention motif from a proportion of physiological substrate molecules, allowing the release of their N-terminal proteolytic fragments into the extracellular space.

## Determining enzymatic function of RBBP9

Many serine hydrolases have been defined on the basis of their reactivity with activity-based probes, but their enzymatic function remains unknown^39^. Defining the activity of these orphan hydrolases remains an outstanding challenge. We set out to address this challenge using Dap-mediated substrate trapping. We focussed on RBBP9, a tumour-associated putative serine hydrolase^5^. RBBP9 possesses a classical α/β hydrolase superfamily fold^40^, and its hydrolase activity promoted tumour cell proliferation during pancreatic neoplasia^41^. Despite multiple efforts to characterize the cellular activity of RBBP9, its enzymatic function in cells has remained enigmatic.

**Fig. 4:**
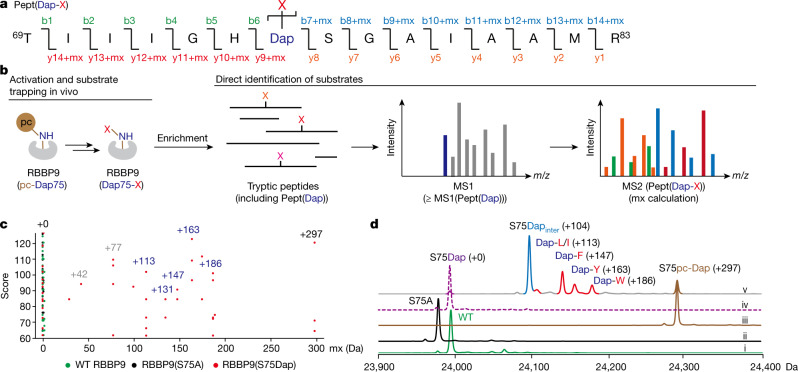
Identifying RBBP9(Dap75) conjugates. **a**, The Dap-containing tryptic peptide sequence (Pept(Dap-X)) of RBBP9 with certain b and y ion masses of Pept(Dap) modified by mx. **b**, Pipeline to identify mx in live cells. RBBP9(Dap75-X) conjugates were affinity purified and trypsinized. The resulting peptide pool was analysed by LC–MS/MS and peptides with molecular mass no less than MS(Pept(Dap)) were individually selected for mx calculation (for example, the blue peak represents Pept(Dap), mx = 0). The experimental MS2 peaks were compared to theoretical MS2 peaks for scoring as described in Methods. In the example shown, the peaks are colour coded as b, y, b+mx or y+mx ions, using the colour scheme in **a**. **c**, mx obtained from the top-scoring spectra were plotted. Each dot represents the mass shift of the observed peptide relative to the parental peptide. +113 (Leu/Ile), +131 (Met), +147 (Phe), +163 (Tyr), +186 (Trp), +297 (pc-Dap), +42 and +77 (consistent with near cognate suppression of this amber codon in mammalian cells with Gln and Tyr, respectively). **d**, The entire mass of RBBP9 variants and Dap conjugates purified from human cells. Green trace (i): wild-type RBBP9 ([Ac-(RBBP9-Met)]: expected 23,995 Da, observed 23,994.5 Da); black trace (ii): RBBP9(S75A) ([Ac-(RBBP9(S75A)-Met)]: expected 23,979 Da, observed 23,978.5 Da); brown trace (iii): RBBP9(S75pc-Dap) before illumination ([Ac-(RBBP9(S75pc-Dap)-Met)]: expected 24,291 Da, observed 24,290.5 Da); purple dashed trace (iv): RBBP9(S75Dap) deprotected from RBBP9(S75pc-Dap) in vitro ([Ac-(RBBP9(S75Dap)-Met)]: expected 23,994 Da, observed 23,993.5 Da); multicolour trace (v): RBBP9(S75pc-Dap) purified from cells after illumination and substrate trapping for 3 h ([Ac-(RBBP9(S75Dap_inter_)-Met)]: expected 24,098 Da, observed 24,097 Da; [Ac-(RBBP9(Dap-L/I)-Met)]: expected 24,107 Da, observed 24,108 Da; [Ac-(RBBP9(Dap-F)-Met)]: expected 24,141 Da, observed 24,140.5 Da; [Ac-(RBBP9(Dap-Y)-Met)]: expected 24,157 Da, observed 24,156 Da; [Ac-(RBBP9(Dap-W)-Met)]: expected 24,180 Da, observed 24,179 Da). The experiment in **c** was performed in biological triplicate. In **d**, the entire mass acquisition of trace (v) was performed in two biological replicates with similar results, the other traces were acquired once.

Initial experiments suggested that RBBP9 is unlikely to function as an endopeptidase (Supplementary Fig. 15). We developed an approach to directly identify any entities (which we designated ‘X’), conjugated to RBBP9(S75Dap), in which the catalytic serine of RBBP9 has been replaced by Dap (Fig. 4a). We first expressed RBBP9(S75Dap) in human cells and enriched any conjugates that it formed (RBBP9(Dap75-X)) in the cells, digested with trypsin, then performed LC–MS/MS on the resulting peptide pool. We then developed a computational pipeline to search for the tryptic peptides of RBBP9 containing Dap and conjugated to X (Pept(Dap-X)), based on the prediction that they should have MS2 spectra related to the corresponding non-conjugated peptide (Pept(Dap)), in which certain fragmentation peaks are shifted by the mass of X (or the mass of a tryptic fragment of X) (mx) (Fig. 4a,b).

Our analysis revealed mass shifts of +113 Da, +131 Da, +147 Da, +163 Da and +186 Da, with respect to the mass of Pept(Dap) (Fig. 4c, Supplementary Fig. 16). These masses correspond to the amino acids leucine/isoleucine (Leu/Ile), methionine (Met), phenylalanine (Phe), tyrosine (Tyr) or tryptophan (Trp), respectively, after condensation on Dap (Supplementary Fig. 17). Additionally, we characterized the entire mass of RBBP9(Dap75-X) and further confirmed the conjugation of Leu/Ile, Phe, Tyr or Trp to RBBP9(S75Dap) (Fig. 4d).

Collectively, we demonstrated that RBBP9(S75Dap) preferentially forms conjugates with individual hydrophobic amino acids. We conclude that RBBP9 is likely to cleave amide (or ester or thioester) bonds to these hydrophobic amino acids in human cells. Amino acid sequence analysis suggested that RBBP9 belongs to DUF1234 hydrolase superfamily^40^, which includes the acylpeptide hydrolase-like protein^42^ from *Arabidopsis thaliana* (AHLP; Uniprot ID: Q9FG66); this protein possesses a similar α/β hydrolase fold to RBBP9 and removes N-terminal hydrophobic residues—especially aromatic amino acids—from peptides. We therefore postulated that RBBP9 may have aminopeptidase activity in human cells.

## RBBP9 is an aromatic aminopeptidase

**Fig. 5:**
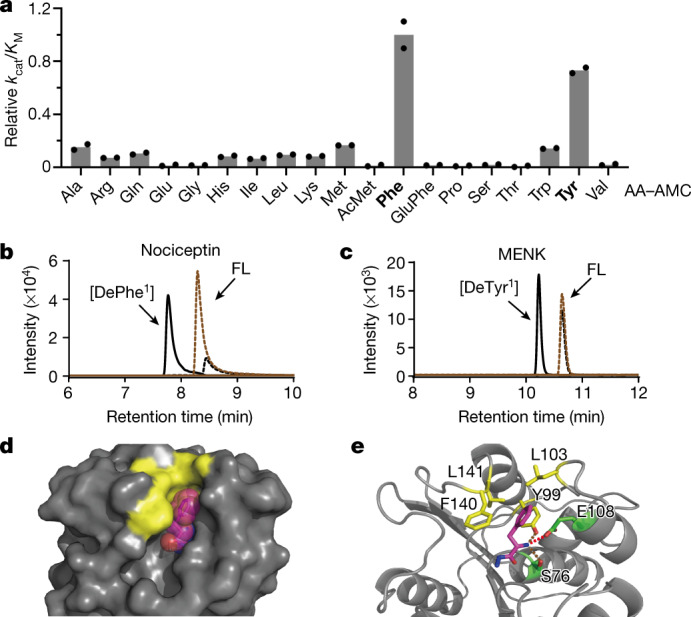
Characterization of RBBP9 aminopeptidase activity. **a**, RBBP9 is specific for aromatic amino acids. The graph shows the catalytic efficiency of RBBP9 on 19 AA–AMCs relative to its catalytic efficiency on Phe–AMC. The bar graph represents the mean of *n* = 2 independent measurements. **b**, **c**, RBBP9 cleaves aromatic amino acids from the N terminus of peptide hormones (nociceptin or MENK). The full-length (FL) peptides and the products (DePhe^1^ or DeTyr^1^) after incubating with wild-type RBBP9 or RBBP9(S75A) were determined by mass spectrometry. Black solid line shows detection of product after incubating with WT RBBP9; brown solid line shows detection of product after incubating with RBBP9(S75A); black dashed line shows detection of full-length peptide after incubating with wild-type RBBP9; brown dashed line shows detection of full-length peptide after incubating with RBBP9(S75A). **d**, **e**, The crystal structure of RBBP9 in complex with Phe. **d**, surface view of RBBP9 and sphere representation of Phe (purple). The surface of Tyr99, Leu103, Phe140 and Leu141 is shown in yellow. **e**, Ribbon diagram of RBBP9 with key residues shown as sticks. Side chains of Tyr99, Leu103, Phe140 and Leu141 are shown in yellow; side chains of Glu108 and Ser76 are shown in green. The hydrogen bond between the α-amine group of Phe and the side chain of Glu108 (2.7 Å) is represented by a red dashed line. The side chain of Ser76 or Tyr99 may also form hydrogen bonds with the α-amino group of Phe (dashed brown lines, bond distances of 2.8 Å or 2.9 Å, respectively). Source data

To investigate the aminopeptidase activity of RBBP9, we performed a fluorescence-based hydrolysis assay for 19 amino acid 7-amino-4-methylcoumarin (AA–AMC) compounds (Fig. 5a, Supplementary Fig. 18). RBBP9 showed a clear preference for hydrolysing aromatic residues, especially Phe and Tyr; this is generally consistent with the mass adducts identified on Dap from live cells. Moreover, RBBP9-mediated hydrolysis required a free α-amine, as acetylated Met–AMC (AcMet–AMC) and the dipeptide Glu-Phe–AMC were not hydrolysed; this explains why the aminopeptidase activity was not detected even though a subset of protease substrates (for example, Pro–pNA and Suc-Phe–pNA) were screened in previous studies^40^.

The aminopeptidase activity and selectivity of RBBP9 was further validated on peptides. As expected, wild-type RBBP9 but not the catalytically inactive S75A mutant removed the first Phe (Phe^1^) from neuropeptide nociceptin (Fig. 5b), the first Tyr (Tyr^1^) from enkephalin (MENK) (Fig. 5c), and the first Trp (Trp^1^) from fibronectin adhesion-promoting peptide, but not the first glycine (Gly^1^) from a tetrapeptide (Supplementary Fig. 19, Supplementary Table 5).

To understand the preference shown by RBBP9 towards aromatic residues, we solved the crystal structure of RBBP9 in complex with Phe (Extended Data Table 1). The overall structure of this complex is almost identical (root mean squared deviation = 0.17 Å) to that of RBBP9 alone^40^. Phe sits in the RBBP9 catalytic pocket (Fig. 5d). Tyr99, Leu103, Phe140 and Leu141 form a hydrophobic cage that holds the bulky aromatic ring of Phe in position (Fig. 5d). In addition, the hydrogen bond formed between the α-amine of Phe and the side chain of Glu108 (or Ser76 or Tyr99) anchors the amino group at this position (Fig. 5e); this explains why a free α-amine is required on the N terminus of RBBP9 substrates. Overall, our results demonstrate that RBBP9 is an aminopeptidase with a preference for removing aromatic residues from the N terminus in human cells. To our knowledge, this is the first reported aminopeptidase in mammals that uses a catalytic serine nucleophile to remove the N-terminal amino acid from polypeptides. Future work should aim to identify the C-terminal portion of RBBP9 substrates and understand how the hydrolase activity we have discovered relates to tumour cell proliferation.

## Discussion

We have demonstrated that adding a Dap-containing protease to a complex mixture facilitates discovery of protease substrates. Extensions of this approach should facilitate substrate discovery in systems where genetic manipulation is challenging, including primary tissue samples. We have demonstrated that we can directly express and rapidly optically activate hydrolase substrate traps in live mammalian cells. As the genetic code expansion methods used to express caged hydrolase substrate traps have now been developed in several model organisms^43,44^, including mice, future work may extend our approach to diverse physiological settings.

**Extended Data Fig. 9:**
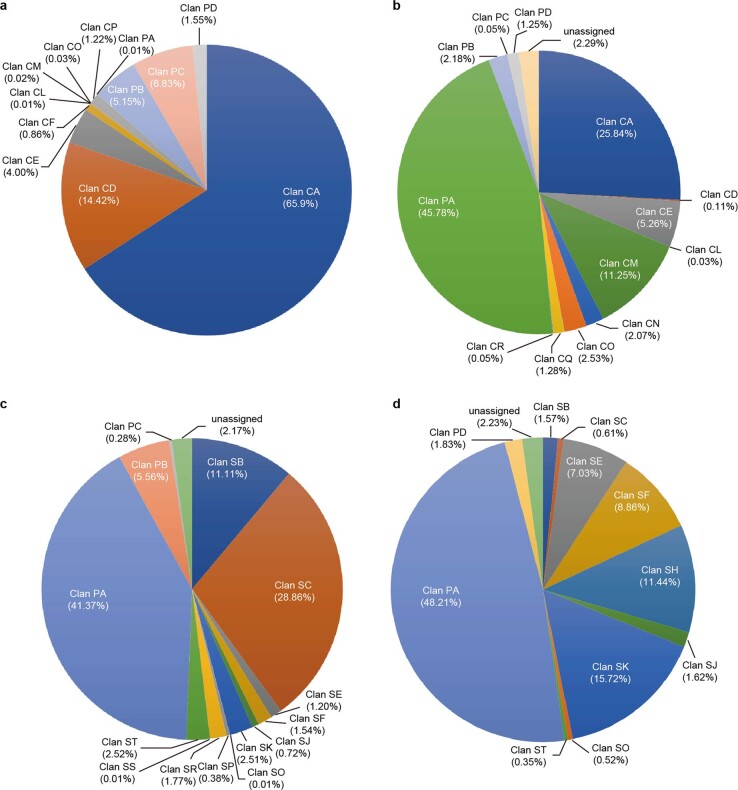
The distribution of cysteine and serine protease clans. The clan distribution of cysteine proteases from (**a**) animals and (**b**) viruses. The clan distribution of serine proteases from (**c**) animals and (**d**) viruses. We have demonstrated the Dap-mediated protease substrate capture method for multiple cysteine proteases (TEV (Clan PA), UL36^USP^ (Clan CA) and SCoV2-PLpro (Clan CA)) and serine proteases (HtrA2 (Clan PA), RHBDL4 (Clan ST) and RBBP9 (Note that RBBP9 has not been included in the MEROPS database by September 30, 2021. RBBP9 possesses the “alpha-beta hydrolase” fold^40^, suggesting that it belongs to Clan SC). The data used to generate this figure were downloaded from the MEROPS database^47^. Source data

Hydrolases have arisen independently multiple times in evolution^45,46^. Proteases that proceed through an acyl-enzyme intermediate naturally divide into two classes on the basis of the stereochemistry of nucleophilic attack^46^. We have exemplified our approach for serine and cysteine proteases from both mechanistic classes. Proteases have also been classified into clans that have a common ancestry, as identified by structural homology^47^. We have exemplified our approach for cysteine proteases from the major clan within animals (Clan PA, 66%) and viruses (Clan CA and PA, 72%), and for the major serine proteases and hydrolase clans within animals (Clans PA and SC, 70%) and viruses (Clan PA, 48%)^47^ (Extended Data Fig. 9); we have also exemplified our approach for both soluble and intramembrane proteases, and provided biological insights (Supplementary Note 2). Thus, our results cover many classes of protease reaction that proceed through an acyl-enzyme intermediate and the majority of protease structural classes; this suggests that our approach will be broadly applicable.

Finally, we have demonstrated the utility of combining hydrolase substrate traps with the direct identification of Dap conjugates to define the molecular function of an orphan hydrolase. We anticipate that future work will extend the approaches we have developed to identify the activities and substrates of many other hydrolases.

## Methods

### Plasmid construction

Standard molecular biology techniques, including PCR, restriction cloning, Gibson assembly, Golden gate assembly, and quik-change mutagenesis were applied to assemble plasmids. To generate plasmids for protein expression in *E. coli*, the DNA fragment encoding human HtrA2(134–458), RBBP9 or SCoV2-PLpro was synthesized as a double stranded DNA (Integrated DNA Technologies (IDT)), and UL36^USP^ (UL36(39–285)) and UL36^USP^(C65S) were amplified from UL36^USP^ containing plasmids^48^. The encoding sequence was cloned into pNHD vector^2^ with a C-terminal HA–Strep-tag for HtrA2 and RBBP9, and a C-terminal twin-Strep-tag for UL36^USP^ and SCoV2-PLpro. To convert catalytic serine/cysteine to alanine or amber stop codon, site directed mutagenesis was completed using quik-change primers (Agilent primer design). To generate vectors for protein expression in mammalian cells, DapRST^2^, TEV^2^, human RHBDL4 (ref. ^49^) or RBBP9 encoding sequence was amplified and introduced into the previously reported pcDNA3.1 plasmid backbone^50^ with 4 × [U6-^Pyl^tRNA_CUA_] . A C-terminal HA–Strep-tag was introduced to TEV, and a twin-Strep-tag was introduced at the C-terminus of RHBDL4, RBBP9, UL36^USP^ or SCoV2-PLpro. The DNA fragments encoding ER-resident proteins—human CCDC47, PDIA6, CALR, GANAB, ERP44, CALU, PRKCSH, FKBP9, DNAJC3 and CANX—were amplified from HEK293T cDNA and cloned into a previously reported pcDNA3.1-based BiP expressing plasmid^51^, in which an ER leader peptide was followed by a V5-tag and BiP encoding sequence. An additional HA-tag was introduced at the C-terminus of CCDC47, or before the ER retention motif sequence of ERP44. The DNA fragments encoding MFN2, LEMD2, EMD, HNRNPH1 and HNRNPM were amplified from HEK293T cDNA and cloned into a pcDNA3.1 plasmid. An HA-tag was placed at the C-terminus of MFN2, LEMD2 or HNRNPM, while a GFP-tag was introduced at the C-terminus of Emerin or HNRNPH1 for better detection of the proteolytic fragments. The guide RNA (gRNA) for RHBDL4 knockout was introduced into pX330-puro plasmid with the optimized scaffold^52^ via Golden gate assembly.

### Western blot

Samples were separated by SDS–PAGE (note that NuPAGE 4–12%, 10% Bis-Tris or 3–8% Tris-Acetate gels running in MES or MOPS buffer were applied to achieve the optimal separation of proteins (protein fragments) of interest) and transferred to polyvinylidene difluoride (PVDF) membrane by iBlot 2 dry blotting system (Thermo Fisher Scientific). Membrane was blocked by Odyssey blocking buffer in PBS (catalogue (cat.) no. 927-40000, Li-Cor) at room temperature for 30 min. Membrane was incubated in primary antibody solution (dilution according to manufacturer’s instructions in Odyssey T20 (PBS) antibody diluent (927-75001, Li-Cor)) at 4 °C overnight. All incubations were carried out on a platform shaker. The membrane was washed three time with PBST (PBS supplemented with 0.1% Tween-20 (v/v)), and incubated with the secondary antibody solution (1: 15,000 (v/v) in PBS blocking buffer supplemented with 0.2% Tween-20 (v/v), and 0.01% SDS) at room temperature for 1 h. After washing 3 times with PBST and once with PBS, the immunoreactive proteins were visualized by the Odyssey CLx imaging system (Li-Cor) by scanning at 700 nm and/or 800 nm channels. Revert 700 Total Protein Stain (926–11015, Li-Cor) was used for total protein staining. The data were analysed by Image Studio Lite (version 5.2.5). For primary and secondary antibodies used in this study, see ‘Antibodies’ in Supplementary Methods.

### Deprotection of pc-Dap containing proteins in buffer

To activate protease(pc-Dap), proteins were illuminated (365 nm, 4 mW cm^−2^) for 1 min in Tris buffer (5 mM DTT, pH 8.0) and incubated at 4 °C or 37 °C overnight to generate protease(Dap). MIC-LED-365 (500 mA, Prizmatix collimated modular Mic-LED light source, Supplementary Fig. 2) was used for illuminating proteins in solution. This apparatus was also used for illuminating suspension cells (Expi293 cells) in tissue culture hood.

### HtrA2 substrate trapping in cell lysate

Thirty micrograms HtrA2–HA–Strep variant (wild type, Ala or Dap) was added to 1 ml of Expi293 cell lysate (3 μg μl^−1^) and incubated at 30 °C for 3 h. The reaction was shaken 10 s every 10 min. Fifty microlitres of anti-HA agarose slurry (A2095, Merck) was added to the reaction and mixed at 4 °C on an end-over-end rocker for 2 h. The mixture was transferred to a Bio-spin column. The resin was washed with RIPA buffer 3 times and PBST buffer 3 times using a vacuum pump, followed by centrifugation at 5,000*g* for 1 min to remove the residual buffer. Then, beads were incubated in 100 μl 1× LDS loading buffer and boiled at 95 °C for 5 min. Twenty microlitres of the eluate was analysed by SDS–PAGE or western blot. Twenty microlitres of eluate was separated in a Bolt 10% Bis-Tris Plus gel for 3 min at 200 V. Gel slices containing all proteins were cut and analysed by LC–MS/MS as described in ‘Electrospray ionization tandem mass spectrometry’.

### Validation of HtrA2 substrates in cell lysate

Wild-type HtrA2 or HtrA2(S306A) (1 μM) was added to 1.2 ml Expi293 cell lysate. At indicated time points, 300 μl of reaction was quenched by mixing with 100 μl 4 × LDS loading buffer and boiled at 95 °C for 5 min. The samples were analysed by western blot with primary antibodies listed in Supplementary Tables 2, 3. GAPDH was used as a loading control.

### Incorporation of pc-Dap in HEK293T cells

HEK293T cells were purchased from European Collection of Cell Cultures (authenticated by STR DNA profiling) and were tested negative for *Mycoplasma* contamination.

HEK293T cells were cultured in Dulbecco’s Modified Eagle Medium (DMEM) (Gibco) supplemented with 10% fetal bovine serum (FBS) (Gibco) and Penicillin-Streptomycin (Pen/Strep, 100 IU ml^−1^ penicillin and 100 μg ml^−1^ streptomycin) at 37 °C in a humidified incubator supplied with 5% CO_2_. Cells were passaged every 2–3 days by detaching with trypsin–EDTA solution, resuspended in DMEM with 10% FBS, and seeded into cell culture flasks.

For transfection in a 24-well plate: 0.75 μl of Lipofectamine 3000 (Thermo Fisher) was diluted in 25 μl Opti-MEM (Gibco) and vortexed briefly. DNA solution was prepared by mixing 500 ng DNA mixture (substrate:DapRST:protease, 1:1:3 or empty vector:DapRST:Protease, 1:1:3) in 25 μl Opti-MEM, followed by adding 1 μl of P3000 reagent. Then, diluted Lipofectamine was added to DNA solution (1:1 v/v). The mixture was incubated at room temperature for 10 min and the DNA–lipid complexes were added to cells. Indicated concentrations in figure legends (or 0.5 mM) of pc-Dap was added to the culture medium 30 min after transfection to achieve pc-Dap incorporation. Cells were incubated at 37 °C for 40–48 h before further analysis.

### Incorporation of pc-Dap in Expi293 cells

Expi293 cells were purchased from Thermo Fisher (authenticated by STR DNA profiling) and were tested negative for *Mycoplasma* contamination.

Expi293 cells were cultured in Expi293 media (Gibco) and shaken at 125 rpm in incubator at 37 °C with 8% CO_2_. Cells were passaged every 2–3 days, starting with the cell density around 0.5 × 10^6^ cells per ml. Transfection was performed at cell density around 2.5 × 10^6^ cells per ml.

Transfection of 100 ml Expi293 cells: 300 μl of polyethyleneimine molecular mass 40,000 (PEI, 1 mg ml^−1^, Polysciences) was diluted in 3.3 ml Expi293 medium. 100 μg DNA mixture (substrate:DapRST:protease, 1:1:3 or empty vector:DapRST:protease, 1:1:3) was diluted in 3.3 mL Expi293 media. Diluted DNA and PEI solution were mixed and incubated at room temperature for 15 min before adding to the cell culture. 0.5 mM (or indicated concentrations in figure legends) of pc-Dap was added 30 min after transfection for pc-Dap incorporation. Forty to forty-eight hours after transfection, the cells were collected and photoactivated for further analysis.

### Photoactivation of protease(pc-Dap) and substrate trapping in mammalian cells

Forty to forty-eight hours after transfection, cell culture medium was replaced with fresh medium, and cells were illuminated for 2 min. The apparatus for illuminating adherent mammalian cells was built in-house (Supplementary Fig. 7). LuxiGen 365 nm UV LED Emitter (LZ4-04UV0R-0000, Mouser Electronics) was used for illumination. The UV intensity at the well plate was set at 4 mW cm^−2^. After illumination, cells were incubated at 37 °C for indicated period of time (Proteasome inhibitor MG132 (2 μM) was added if needed). For adherent cells, at each time point, cells in a 6-well plate were washed with PBS and lysed in 400 μl RIPA lysis buffer (89900, Thermo) supplemented with Halt Protease Inhibitor Cocktail (78429, Thermo Fisher) and the Universal Nuclease (88702, Thermo Fisher). The lysate was cleared by centrifuging at 21,000*g* for 5 min and the supernatant was flash frozen and stored at −80 °C. For suspension cells, at each indicated time point, 5 ml cell culture was centrifuged at 650*g* for 5 min and the cell pellet was flash frozen and kept at −80 °C. Then, cell pellets were lysed in 1 ml RIPA lysis buffer supplemented with protease inhibitors and the Universal Nuclease at 4 °C. Cell lysates were centrifuged at 21,000*g* for 5 min. The cleared lysates were used for SDS–PAGE and western blot analysis or affinity enrichment by MagStrep type3 XT beads (2-4090-002, IBA).

### Trapping endogenous substrates to RHBDL4(S144Dap)

RHBDL4 variants were expressed in Expi293 cells as described in ‘Incorporation of pc-Dap in Expi293 cells’. Forty hours after transfection, 50 ml cell culture was resuspended in fresh Expi293 media and illuminated (365 nm, 4 mW cm^−2^) for 2 min. Cells were incubated at 37 °C for 4 h in the presence of 2 μM MG132 before collection. After pelleting, cells were resuspended in HEPES buffer (50 mM HEPES, pH 7.4, 150 mM NaCl, 1 mM MgCl_2_, 5% glycerol, 1 mM DTT) supplemented with protease inhibitors and the Universal Nuclease. The suspension was lysed by passing twice through an Avestin Emulsiflex C3 homogenizer (ATA Scientific) at 3,000–5,000 psi. The lysate was centrifuged at 1,000*g* for 5 min twice and the supernatant was further centrifuged at 100,000*g* for 1 h. The pellet was washed with Na_2_CO_3_ (100 mM, pH 11.3) at 4 °C for 20 min and then centrifuged at 140,000*g* for 1 h. The membrane fraction was dissolved in 2% SDS buffer (50 mM Tris, pH 8, 150 mM NaCl, 1 mM DTT and protease inhibitors). The solution was diluted by 10% NP40 to generate a final concentration of 0.1% SDS and 1% NP40. One-hundred microlitres MagStrep type3 XT beads were added to the solution and incubated at room temperature for 1 h. The beads were washed with RIPA and PBST three times each. Proteins attached to the beads were eluted in 1× LDS loading buffer by heating at 65 °C for 15 min. The eluates were separated in a Bolt 10% Bis-Tris Plus gel for 3 min. Gel slices containing proteins were cut and analysed by LC–MS/MS.

### RHBDL4 cleavage assay

Empty vector, wild-type (WT) RHBDL4 or RHBDL4(S144A) plasmid was co-transfected with candidate substrate containing plasmid or empty vector (for endogenous substrates) into HEK293T or Expi293 cells. The amount of candidate substrate-containing plasmid was optimized for expression level. Forty to forty-eight hours after transfection, cells were collected and lysed in RIPA buffer supplemented with protease inhibitors and the Universal Nuclease. The lysate was cleared and analysed by western blot.

To analyse proteins in the extracellular medium, FBS-containing medium for HEK293T cells was replaced with hybridoma serum free medium (12045076, Thermo Fisher) 24 h before collection. Expi293 medium, which is serum-free and protein-free, can be directly collected for further analysis. The medium was collected and filtered through a 0.22 μm polyethersulfone membrane. To obtain total proteins in the medium, 1/10 volume of 100% ice-cold TCA solution (T0699, Sigma) was added at 4 °C for protein precipitation. To obtain proteins in the supernatant, the medium was centrifuged at 200,000*g* for 1 h to separate supernatant from microvesicles. The SN was collected and added 1/10 volume of ice-cold TCA to precipitate proteins. After centrifuging at 21,000*g* for 10 min, the precipitate was washed once with acetone, and dissolved in 1× LDS loading buffer. The microvesicles pellet after ultra-centrifugation was washed with PBS once and dissolved in equal volume of 1× LDS loading buffer.

Deglycosylation was performed by adding 1/10 volume of 10% NP40 and PNGase (P0704S, NEB) or DeGlycosylation mix II (P6044S, NEB) to proteins dissolved in the 1× LDS loading buffer. The reaction was incubated at 37 °C for 1 h before analysis.

### Brefeldin A inhibitory assay

Twenty-four hours after transfection, Expi293 cells were split into two halves treated separately with DMSO or BFA (5 μg ml^−1^). The BFA treatment was performed in two ways: (1) BFA was directly added into medium culture; (2) the medium culture was replaced with fresh medium supplemented with BFA. Sixteen hours after BFA treatment, the cells and extracellular medium were collected and analysed as described in ‘RHBDL4 cleavage assay’.

### Knockout of RHBDL4

HCT116 cells were purchased from American Type Culture Collection (authenticated by STR DNA profiling) and were tested negative for *Mycoplasma* contamination.

HCT116 cells were cultured in McCoy’s 5A (modified) Media (Gibco) supplemented with 10% fetal bovine serum (FBS) (Gibco) and Pen/Strep at 37 °C in humidified incubator with 5% CO_2_. Cells were passaged every 2–3 days.

HCT116 cells in 6-well plates were transfected by Lipofectamine LTX (15338100, Thermo Fisher) with 2.5 μg of pX330-puro plasmid containing gRNA (5′-TCCAGTAAGTACAGAAAATG-3′) and Cas9 for RHBDL4 knockout. Twenty-four hours after transfection, cells were trypsinized and plated in a 10 cm petri dish. After 24 h, the cells were treated with puromycin (1 μg ml^−1^). The puromycin selection stopped after 48 h. Cells were trypsinized and limited dilution was performed to generate single clones, which were expanded and analysed by western blot (anti-RHBDL4) and genotyped by sequencing the genomic DNA region targeted by the gRNA.

To detect the proteolytic fragments from endogenous substrates generated by endogenous RHBDL4, 10 million wild-type or RHBDL4 knockout HCT116 cells were cultured in hybridoma serum free medium for 40 h. The medium was collected, filtered and concentrated by a 30 kDa cut-off concentrator. Proteins were precipitated by TCA and dissolved in 1× LDS loading buffer for immunoblotting analysis.

### Trapping endogenous substrates to RBBP9(S75Dap)

To identify X attached to Pept(Dap), RBBP9 variants were expressed in HEK293T cells as described in ‘Incorporation of pc-Dap in HEK293T cells’. pc-Dap (0.1 mM) was added to cells to produce RBBP9(S75pc-Dap). To characterize the entire masses of RBBP9 variants, RBBP9 variants were produced in 100 ml Expi293 cells as described in ‘Incorporation of pc-Dap in Expi293 cells’. RBBP9(S75pc-Dap) was expressed in the presence of 0.5 mM pc-Dap. 40 h after transfection, cells were illuminated (365 nm, 4 mW cm^−2^) for 2 min, and incubated at 37 °C for 3 h. Cells were then collected and lysed in Tris buffer (50 mM Tris, pH 8.0, 150 mM NaCl, 1 mM EDTA and Universal Nuclease) by sonication. Note that protease inhibitors were not added in lysis buffer. The lysate was cleared by centrifuging at 21,000*g* for 20 min. RBBP9 species in the supernatant were enriched using MagStrep type3 XT beads. Proteins attached to beads were eluted in 50 mM Biotin in Tris buffer for mass characterization.

### Aminopeptidase assay of RBBP9

#### Fluorescence-based assay

Two micromolar RBBP9 was incubated with each AA–AMC over a range of different substrate concentrations in Tris buffer (100 mM Tris, 150 mM NaCl, pH 7.3). Fluorescence intensity (due to the release of the AMC fluorophore by hydrolysis of AA–AMC by RBBP9) was measured every 20 s over a 10-min period (MARS Data Analysis Software (version 3.20 R2)). For each substrate, the rate of fluorescence increase was converted to rate of product formation using standard curves. At substrate concentrations of greater than 10 μM, intermolecular quenching of AMC fluorescence by AA–AMC was found to be significant. Therefore, for all substrates other than Phe-AMC and Tyr-AMC, AA-AMC concentrations between 0 and 4 μM were used, and pseudo-first order kinetics were employed to calculate specificity constants. For Phe-AMC and Tyr-AMC, which showed significantly faster rates of hydrolysis when compared to the other substrates, a concentration range of 0 to 160 μM was used and converted rates were fitted to Michaelis–Menten kinetics in order to obtain specificity constants.

#### Peptide-based assay

Peptides (100 μM) were dissolved in Tris buffer (100 mM Tris pH 7.4, 150 mM NaCl, 1 mM EDTA). Two micromolar wild-type RBBP9 or RBBP9(S75A) was added to start the hydrolysis reaction. The reaction was stopped by quenching with acetic acid and monitored by mass spectrometry. Selected ion mass (SIM) mode was applied for detection of peptide substrates and the desired products.

### Protein crystallization and data collection

Human RBBP9 with a C-terminal His-tag^40^ (LEHHHHHH) was expressed in BL21(DE3) cells and purified by a two-step protocol consisting of HisTrap enrichment and gel filtration (Superdex 75) chromatography. Pure fractions of RBBP9 (> 98% purity determined by SDS–PAGE) were concentrated with a 10 kD MWCO Vivaspin 20 concentrator (Sartorius) to 10 mg ml^−1^ in buffer containing 10 mM Tris (pH 7.5), 100 mM NaCl, 5 mM DTT and 5 mM Phe. Prior to crystallization, samples were cleared by centrifugation for 15 min at 10,000*g*. Crystallization trials with multiple commercial crystallization kits were performed in 96-well sitting-drop vapor diffusion plates (Molecular Dimensions) at 18 °C and set up with a mosquito HTS robot (TTP Labtech). Drop ratios of 0.2 μl protein solution plus 0.2 μl reservoir solution were used for coarse and fine screening. Initial hits were obtained under multiple conditions and required no further optimization. Data was collected from crystals collected from following conditions: 30% w/v PEG 4K, 0.1 M MES sodium salt, pH 6.5.

To ensure cryo-protection, crystal-containing drops were mixed with 25% glycerol in reservoir solution prior to picking and flash frozen in liquid nitrogen. Diffraction data was collected at the Diamond Light Source (DLS, UK) on beamline I04. Datasets were auto-processed with XIA2 DIALS (version 0.7.90), scaled using Aimless and Refmac5 (version 5.8.0258) in the CCP4 suite (version 7.0.078) of programs. Structure refinement and manual model building were performed with Refmac5 and COOT (version 0.8.9.2). Colour figures were prepared with PyMol (version 2.5).

### Mass characterization

#### Electrospray ionization mass spectrometry

Mass spectra of all protein samples were acquired on an Agilent 1200 LC-MS system equipped with a 6130 Quadrupole spectrometer. A Phenomenex Jupiter C4 column (150 × 2 mm, 5 μm) was used to elute proteins. Buffer A (0.2% formic acid in H_2_O) and buffer B (0.2% formic acid in acetonitrile) was used for RP-HPLC. Mass spectra were acquired in the positive mode and analysed by the MS Chemstation software (Rev.C.01.06[61], Agilent Technologies). The deconvolution program provided in the software was used to obtain the entire mass spectra. Theoretical molecular mass of proteins with non-canonical amino acids was calculated by correcting the calculated molecular mass of wild-type protein (http://www.peptidesynthetics.co.uk/tools/) with the molecular mass of non-canonical amino acids.

#### Electrospray ionization tandem mass spectrometry

Proteins (including TEV-GFP conjugate, substrates trapped to HtrA2 or RHBDL4) in polyacrylamide gel slices (1–2 mm) were enzymatically digested in situ for LC–MS/MS analysis. In brief, the excised protein gel pieces were placed in a 96-well microtitre plate and destained with 50% v/v acetonitrile and 50 mM ammonium bicarbonate, followed by reduction with 10 mM DTT and alkylation with 55 mM iodoacetamide. RBBP9 eluates in solution were treated in two ways: (1) incubation at room temperature overnight in the presence of 10 mM DTT without alkylation; (2) reduction with 10 mM DTT for 30 min and alkylation with 55 mM iodoacetamide. Then, proteins were digested with trypsin/LysC (Promega) overnight at 37 °C. The resulting peptides were extracted in 2% v/v formic acid, 2% v/v acetonitrile and analyzed by nanoscale capillary LC-MS/MS, which uses an Ultimate U3000 HPLC (ThermoScientific Dionex) with a flow rate of 300 nl min^−1^. A C18 Acclaim PepMap100 5 μm, 100 μm × 20 mm nanoViper (ThermoScientific Dionex) was used to trap the peptides before separation on a C18 Acclaim PepMap100 3 μm, 75 μm × 150 mm nanoViper (ThermoScientific Dionex). Peptides were eluted with a gradient of acetonitrile. The eluate was directly introduced to a modified nanoflow ESI source with a hybrid dual pressure linear ion trap mass spectrometer (Orbitrap Velos, ThermoScientific). Data-dependent analysis was carried out using a resolution of 30,000 for the full MS spectrum, followed by ten MS/MS spectra in the linear ion trap. MS spectra were collected over an *m*/*z* range of 100–2,000.

#### LC–MS/MS data analysis by Venn diagram

LC–MS/MS data were searched against an in-house protein sequence database containing Swiss-Prot and the protein constructs specific to the experiment, using the Mascot search engine program (Matrix Science, version 2.4). Database search parameters were set with a precursor tolerance of 5 p.p.m. and a fragment ion mass tolerance of 0.8 Da. Variable modifications for oxidized methionine, carbamidomethyl cysteine, pyroglutamic acid, and deamination of glutamine/asparagine were included. MS/MS data were validated using the Scaffold program (version 5, Proteome Software Inc.).

#### LC-MS/MS data analysis by volcano plot

For quantitative analysis, MS raw files were processed by MaxQuant software (version 1.6.3.4) and searched with the embedded Andromeda search engine against the corresponding database (Uniprot). The required FDR was set to 1% or 5% at peptide and protein levels. The maximum number of allowed missed cleavages was set to two. Protein quantification was done by LFQ with default settings. The MaxQuant ProteinGroups output file was further processed with Perseus (version 1.6.2.3)^53^. Contaminations and reverse hits were removed by filtering. The remaining protein quantifications were log_2_-transformed.

### Determination of X attached to Dap in RBBP9

LC–MS/MS files (in RAW format) were first converted to mzML format^54^ using ProteoWizard (version 3.0.11252)^55^. Data preparation and processing were then performed using custom Python (version 3.8.1) scripts written with the pyOpenMS package (version 2.4.0)^56^. In brief, collected spectra were centroided and all MS2 spectra with a precursor mass lower than that of the unconjugated Dap-containing tryptic peptide from RBBP9 (Pept(Dap)) were filtered out. For each filtered MS2 spectrum, the ten most abundant peaks in each 100 Th mass interval were extracted.

Based on the peptide sequence of Pept(Dap) and the precursor mass for each MS2 spectrum, a list of theoretical ion masses was calculated; these corresponded to the MS2 fragmentation of a substrate-conjugated Pept(Dap), (Pept(Dap-X)). This list contained the monocationic b- and y-ions, the dicatonic b- and y- ions, and ions corresponding to water or ammonium losses from the side-chains of b- or y- ions. Peaks in the MS2 spectrum were matched against this list, and a score for this matching was calculated as previously described^57^. This score was ten times the negative logarithm of the approximate probability that at least *k* out of *n* masses have been matched by chance, where *k* is the number of matches and *n* is the number of masses in the list.

To extract the top-scoring spectra, the family-wise error rate for the probability values was controlled at 0.05 using the Bonferroni correction. The mass difference between Pept(Dap) and the precursor ion for each Pept(Dap-X) spectrum was calculated to determine the molecular mass of each conjugate. For each mass shift, representative top-scoring spectra were manually interrogated to verify the assignment.

### Reporting summary

Further information on research design is available in the Nature Research Reporting Summary linked to this paper.

## Online content

Any methods, additional references, Nature Research reporting summaries, source data, extended data, supplementary information, acknowledgements, peer review information; details of author contributions and competing interests; and statements of data and code availability are available at 10.1038/s41586-022-04414-9.

### Supplementary information

Supplementary Information The Supplementary Information contains Supplementary Notes 1 and 2, Supplementary Figures 1-19 (in which the Supplementary Fig. 1 contains all uncropped SDS-PAGE and western blots), Supplementary Tables 1-5, Supplementary Methods and Supplementary Data Files 1 and 2.

Reporting Summary

### Source data

Source Data Fig. 5

Source Data Extended Data Fig. 3

Source Data Extended Data Fig. 9

## Figures and Tables

**Extended Data Table 1:**
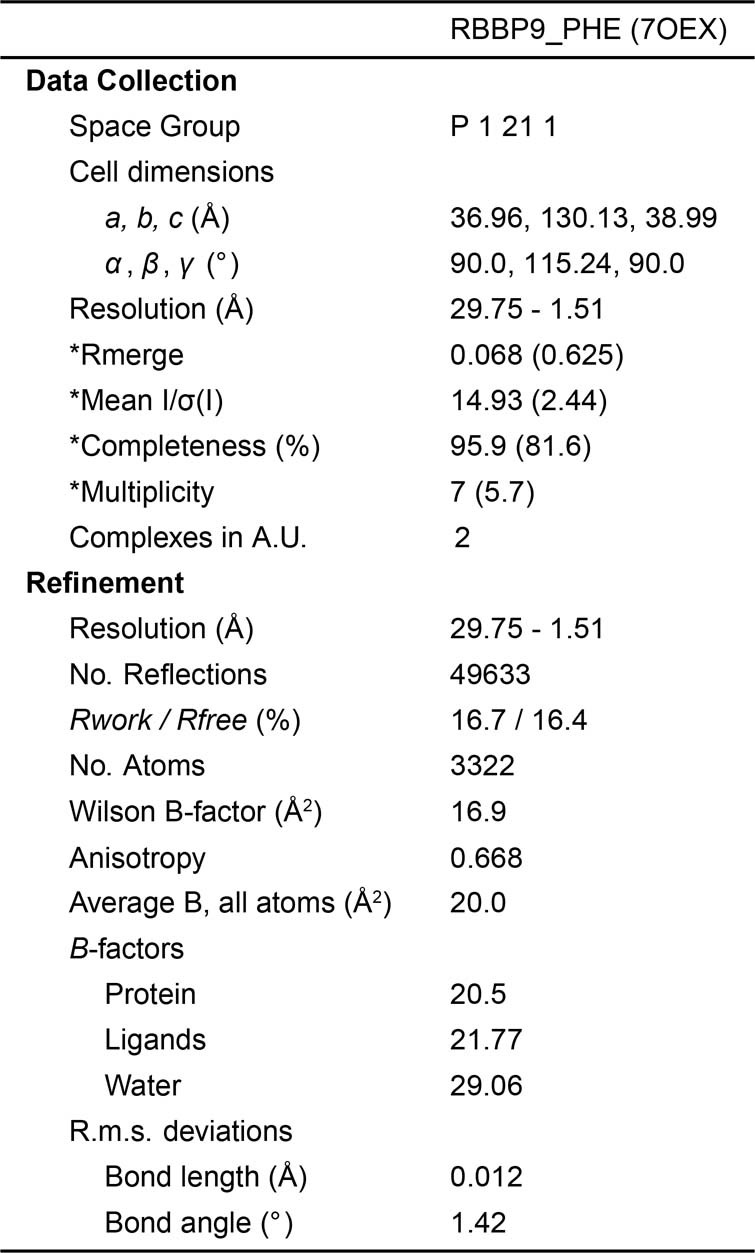
Summary of crystallographic information

* Highest resolution shell (in Å) shown in parenthesis.

## Data Availability

The structure of RBBP9 in complex with Phe is available in the Protein Data Bank under accession code 7OEX. The mass spectrometry proteomics data have been deposited to the ProteomeXchange Consortium via the PRIDE partner repository with the accession number PDX030381. All other datasets and materials generated or analysed in this study are available from the corresponding authors upon reasonable request. The data used to analyse serine and cysteine proteases clans were downloaded from the MEROPS database (https://www.ebi.ac.uk/merops/). Source data are provided with this paper.

## Code availability

The code used for RBBP9 substrate identification by proteomic analysis is available at 10.5281/zenodo.5768340.
