# Supplementary material for: Mechanism-based traps enable protease and hydrolase substrate discovery
Source: Nature. 2022 Feb 16;602(7898):701–7. doi: 10.1038/s41586-022-04414-9 (PMC8866121; doi:10.1038/s41586-022-04414-9)
Supplement: Supplementary file 1 — The Supplementary Information contains Supplementary Notes 1 and 2, Supplementary Figures 1-19 (in which the Supplementary Fig. 1 contains all uncropped SDS-PAGE and western blots), Supplementary Tables 1-5, Supplementary Methods and Supplementary Data Files 1 and 2. [file 41586_2022_4414_MOESM1_ESM.pdf]

nature portfolio

https://doi.org/10.1038/s41586-022-04414-9

Supplementary information

Mechanism-based traps enable protease and hydrolase substrate discovery

In the format provided by the authors and unedited

Supplementary Information

Mechanism-based traps enable protease and hydrolase substrate discovery

Shan Tang[1,*], Adam T. Beattie[1], Lucie Kafkova[2], Gianluca Petris[1], Nicolas Huguenin-Dezot[1], Marc Fiedler[1], Matthew Freeman[2], Jason W. Chin[1,*]

[1]Medical Research Council Laboratory of Molecular Biology, Francis Crick Avenue, Cambridge, UK

[2]Sir William Dunn School of Pathology, University of Oxford, Oxford, UK

[*]Correspondence: stang@mrc-lmb.cam.ac.uk; chin@mrc-lmb.cam.ac.uk

Table of Contents

Pages 3-7: Supplementary Notes

Pages 8-26: Supplementary Figure 1

Pages 27-46: Supplementary Figures 2-19

Pages 47-56: Supplementary Tables

Pages 57-61: Supplementary Methods

Pages 62-70: Supplementary Data File 1

Pages 71-73: Supplementary Data File 2

Page 74-75: Supplementary References

Supplementary Note 1

We primarily focused on characterizing RHBDL4 substrates in the ER, since prior work has shown that RHBDL4 cleaves ER-resident substrates in the ER lumen[1-3]. However, we also identified several proteins reported to reside outside the ER in the RHBDL4(S144Dap) immunoprecipitation with respect to the WT RHBDL4 or RHBDL4(S144A) controls (Supplementary Table 4). The majority of these additional proteins are nuclear.

We considered several possible explanations for the identification of non-ER resident proteins: 1) A fraction of RHBDL4 is located outside the ER and processes substrates in other compartments, 2) during cell lysis and immunoprecipitation, RHBDL4(S144Dap) forms conjugates with proteins that are localized to other compartments within cells, and 3) a fraction of RHBDL4 is inserted into the ER membrane with an inverted topology in which it is able to process substrates in the nucleus (or cytosol).

1) RHBDL4 is reported to reside primarily in the ER. Immunofluorescence (Supplementary Figure 9) demonstrates that WT RHBDL4 and RHBDL4(S144pc-Dap) have comparable localization patterns. Both proteins localize to the ER and to the nuclear membrane. While we cannot rule out that a small fraction of RHBDL4 – below the detection limit of our IF experiments – is elsewhere in the cell, our results provide evidence that the localization of RHBDL4(S144pc-Dap) mirrors that of the WT protein, and that the vast majority of both proteins are found in the ER membrane and nuclear membrane; this is consistent with most previous observations for the localization for RHBDL4[1,4]. Thus, our data do not support mis-localization of RHBDL4(S144Dap) as an explanation for the selective capture of non-ER resident proteins.

2) To investigate whether RHBDL4-mediated cleavage of nuclear-localized proteins occurs after cell lysis, rather than in intact cells, we performed post-lysis control experiments (Supplementary Fig. 11). In post-lysis controls, we mixed cells expressing WT RHBDL4 with cells expressing potential substrates and lysed the cell mixture. Post-lysis controls for HNRNPH1 and HNRNPM demonstrated that very little, if any cleavage occurred in the cell lysate, and indicated that the cleavage of these nuclear substrates – observed when they are co-expressed with WT RHBDL4 – primarily occurs in intact cells. Since conjugation of RHBDL4(S144Dap) to substrates will be slower than complete proteolysis we anticipate that it does not commonly occur post-lysis and that the capture of potential substrates occurs in intact cells.

3) Several multi-span transmembrane proteins are known to exhibit topological inversion[5,6], in which a fraction of the protein molecules is inserted into the membrane in an inverted orientation. For RHBDL4, topological inversion would lead to a different orientation of the transmembrane helices. In the classical topology the catalytic serine at position 144 of RHBDL4 faces towards the ER lumen or extracellular space, while in the inverted topology the catalytic serine faces towards the cytosol or nucleus. The classical topology is expected to cleave ER resident substrates, and would place the C-terminus of the protein in the cytosol, consistent with reports that ubiquitin-interacting motif (UIM)[1] and VCP-binding motif (VBM)[7] at the C-terminus of RHBDL4 bind to ubiquitinated proteins and p97 respectively in the cytosol. In the inverted topology the catalytic serine is predicted to face away from the ER lumen and towards the nucleus or cytosol; consistent with this topology, we observed cleavage of nuclear proteins (HNRNPH1 and HNRNPM) – which were most enriched by immunoprecipitation with RHBDL4(S144Dap) – by WT RHBDL4 (Supplementary Fig. 11). Our observations support a model in which a fraction of overexpressed RHBDL4 has its active site facing

the ER lumen and a fraction has its active site facing the nucleus. We note that the distribution of WT RHBDL4 topologies in the membrane may be a function of its expression level in the cell.

Supplementary Note 2

Intramembrane proteases, like RHBDL4, are distinct from soluble proteases[8], and their membrane embedded enzyme active site architecture makes biochemical analysis even more challenging[9]. Thus, despite intramembrane proteases being widespread and conserved across all domains of life[8,10], the identification of their substrates by classical methods has been exceptionally difficult. We have demonstrated the utility of directly expressing and optically activating protease substrate traps in human cells through the discovery of substrates for RHBDL4. These experiments revealed that RHBDL4 cleaves the ectodomain from CCDC47 (a type I transmembrane protein involved in chaperoning the folding and assembly of multi-span membrane proteins), leading to the secretion of luminal fragments. This suggests that RHBDL4 can have a sheddase-like function, consistent with reports of a similar RHBDL4 activity on APP[3]. We note that rhomboid-related protein 2 (RHBDL2), which resides in the plasma membrane, performs an analogous cleavage in the soluble region of type I transmembrane protein substrates[11].

Strikingly, we discovered that several soluble ER-resident proteins are cleaved by RHBDL4. The majority of these soluble proteins are chaperones – including BiP, PDIA6 and Calreticulin. RHBDL4 cleaves these soluble ER-resident proteins before the ER-retention motif under physiological conditions and promotes their secretion. These observations suggest a non-canonical secretase activity for RHBDL4.

The RHBDL4-mediated cleavage of specific soluble proteins is consistent with several previous observations. 1) The proteolysis of certain type I membrane proteins (RPN1[12], and CCDC47 in the present work (Extended Data Figure 6d, e)) in their luminal domains is not dependent on their transmembrane domains, and their soluble ectodomains are cleaved at the same positions as the full-length protein. 2) the flexible

loops of soluble ERAD luminal substrates can enter the active site from the ER lumen and be cleaved[12]. Thus – in contrast to the *E. coli* rhomboid protease GlpG[13,14] – the opening of the RHBDL4 active-site to facilitate substrate cleavage does not require the engagement of the destabilized transmembrane domain in substrates. It will be interesting to understand the molecular mechanisms through which RHBDL4 engages with soluble substrates, and we note that capturing the acyl-enzyme intermediate of RHBDL4(S144Dap) in complex with soluble substrates may facilitate further mechanistic insight[15].

Why does RHBDL4 proteolyze ER-resident chaperones under physiological conditions? Previous work reported that RHBDL4 proteolysis promotes the ERAD of certain substrates[1,2,12]. However, the RHBDL4 chaperone substrates we have identified are not targeted for ERAD. We suggest two limiting hypotheses for the function of RHBDL4 mediated chaperone cleavage. 1) RHBDL4-mediated proteolysis contributes to the turnover of the chaperones under physiological conditions. In some cases, RHBDL4 cleavage could remove the excess component of a complex (e.g., the Glucosidase 2 subunit α and β) to keep protein complex homeostasis. The proteolytic fragments could be further degraded through ERAD – as observed for oligosaccharyltransferase components[2] – or secreted to the extracellular space through the secretory pathway. 2) RHBDL4-mediated secretion of chaperone fragments could lead to co-secretion of unfolded or improperly folded clients; this would provide a route to remove clients that cannot be refolded by the chaperone, and limit the ER stress[16]. Consistent with this hypothesis, the RHBDL4-mediated cleavage of some chaperones (e.g., BiP, Calreticulin, PDIA6) leads to secretion of near full-length chaperones, whose functional domains remain intact, and only a fraction of the total chaperone pool is cleaved.

Supplementary Figure 1: Uncropped western blots for Figure 1

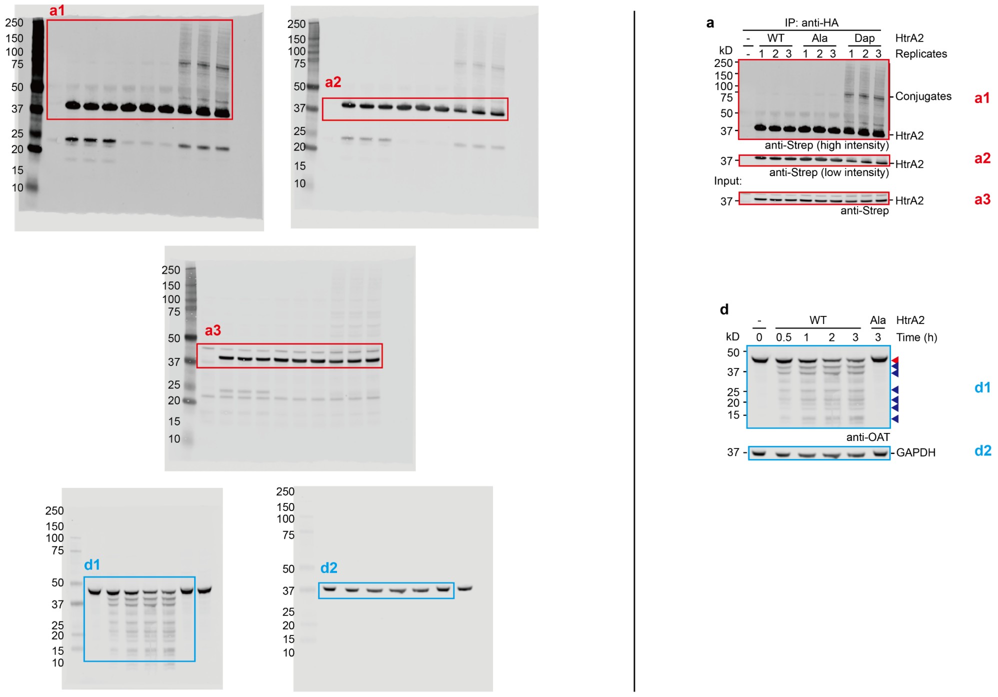

Supplementary Figure 1 (continued): Uncropped western blots for Figure 2

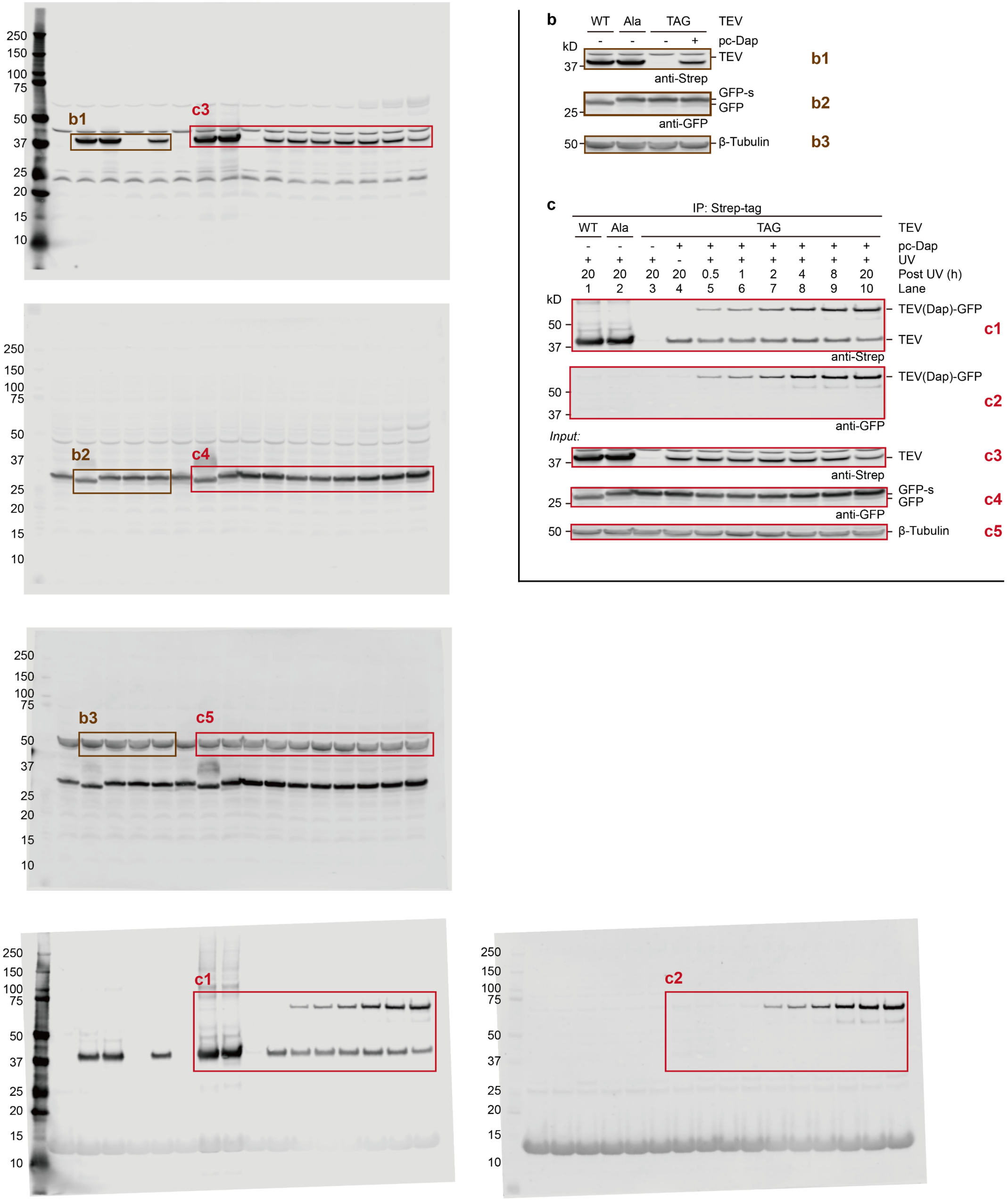

Supplementary Figure 1 (continued): Uncropped western blots for Figure 3

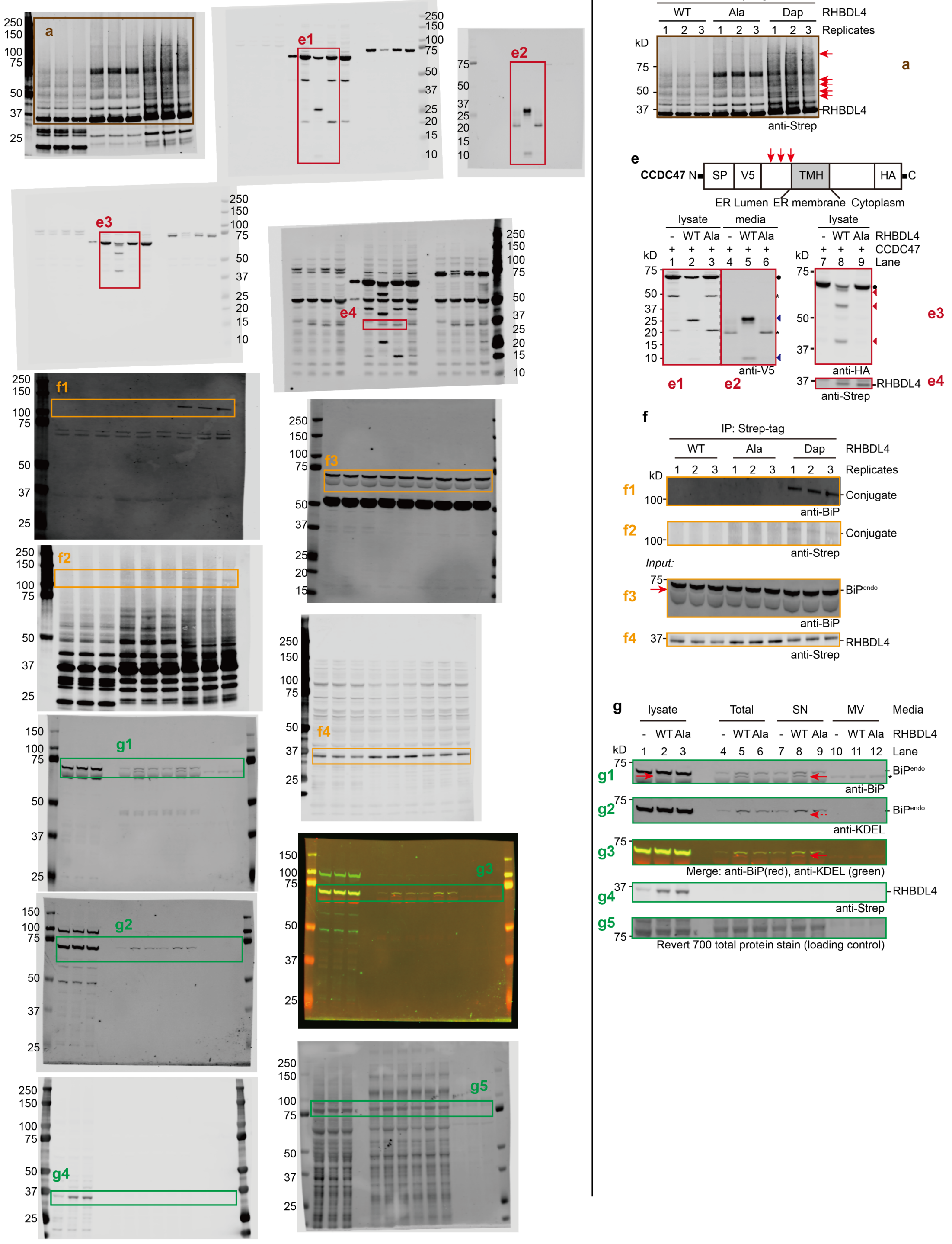

Supplementary Figure 1 (continued): Uncropped SDS-PAGE and western blots for Extended Data Figure 2

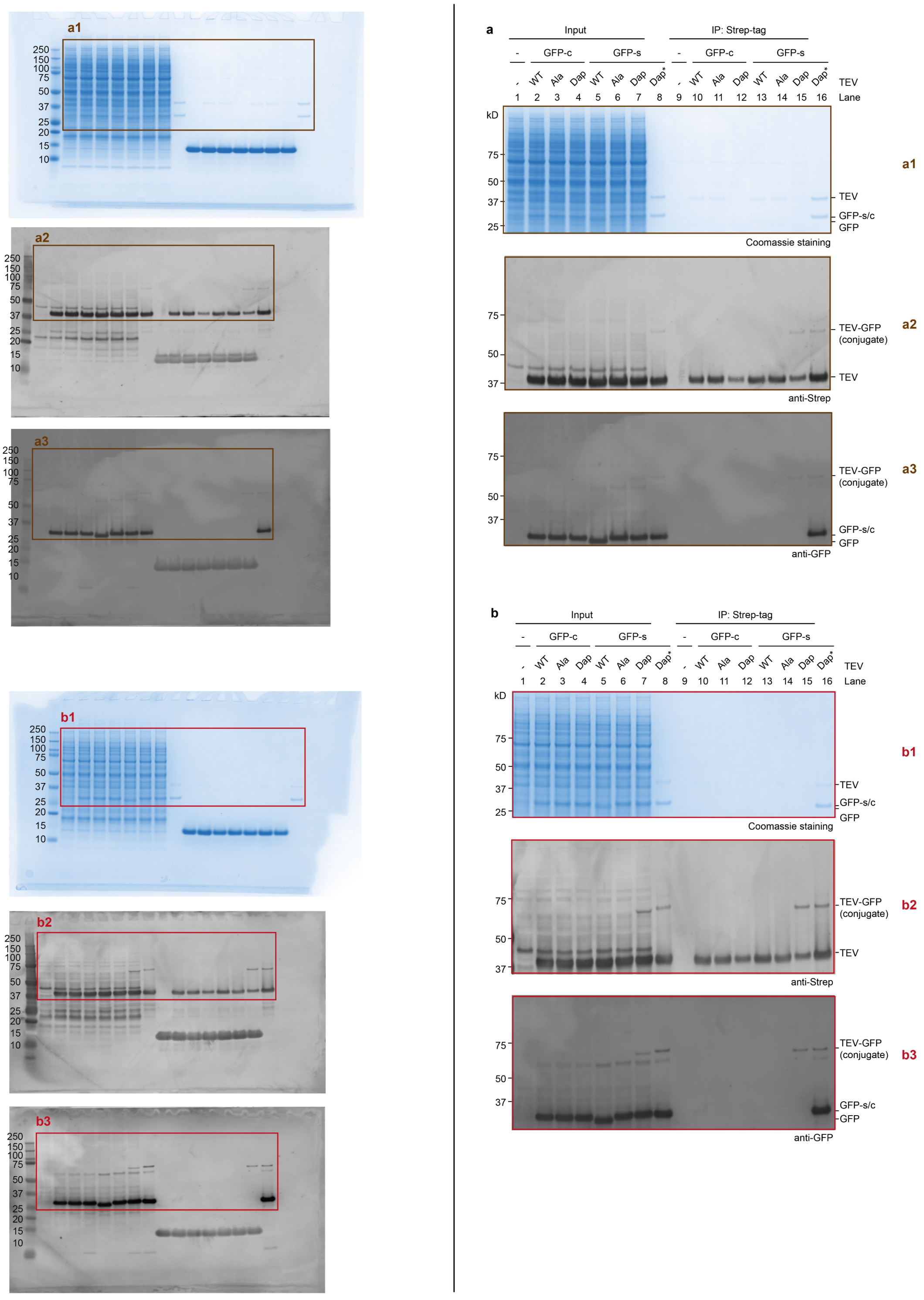

Supplementary Figure 1 (continued): Uncropped western blots for Extended Data Figure 3

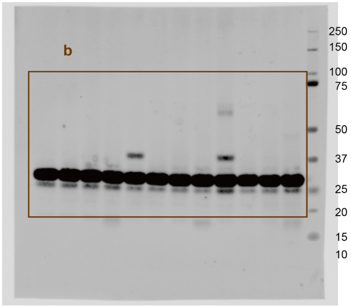

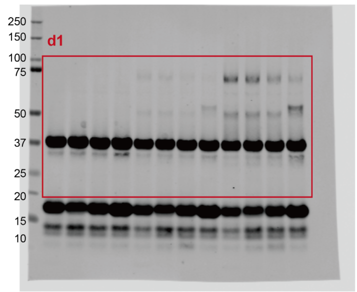

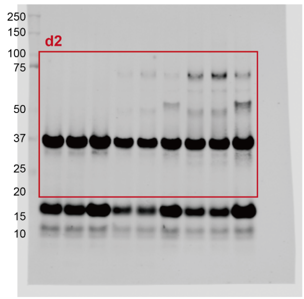

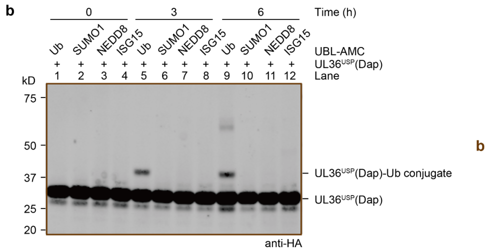

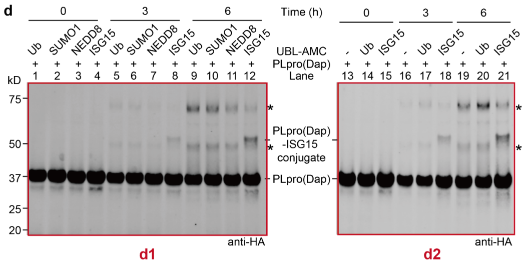

Supplementary Figure 1 (continued): Uncropped western blots for Extended Data Figure 4

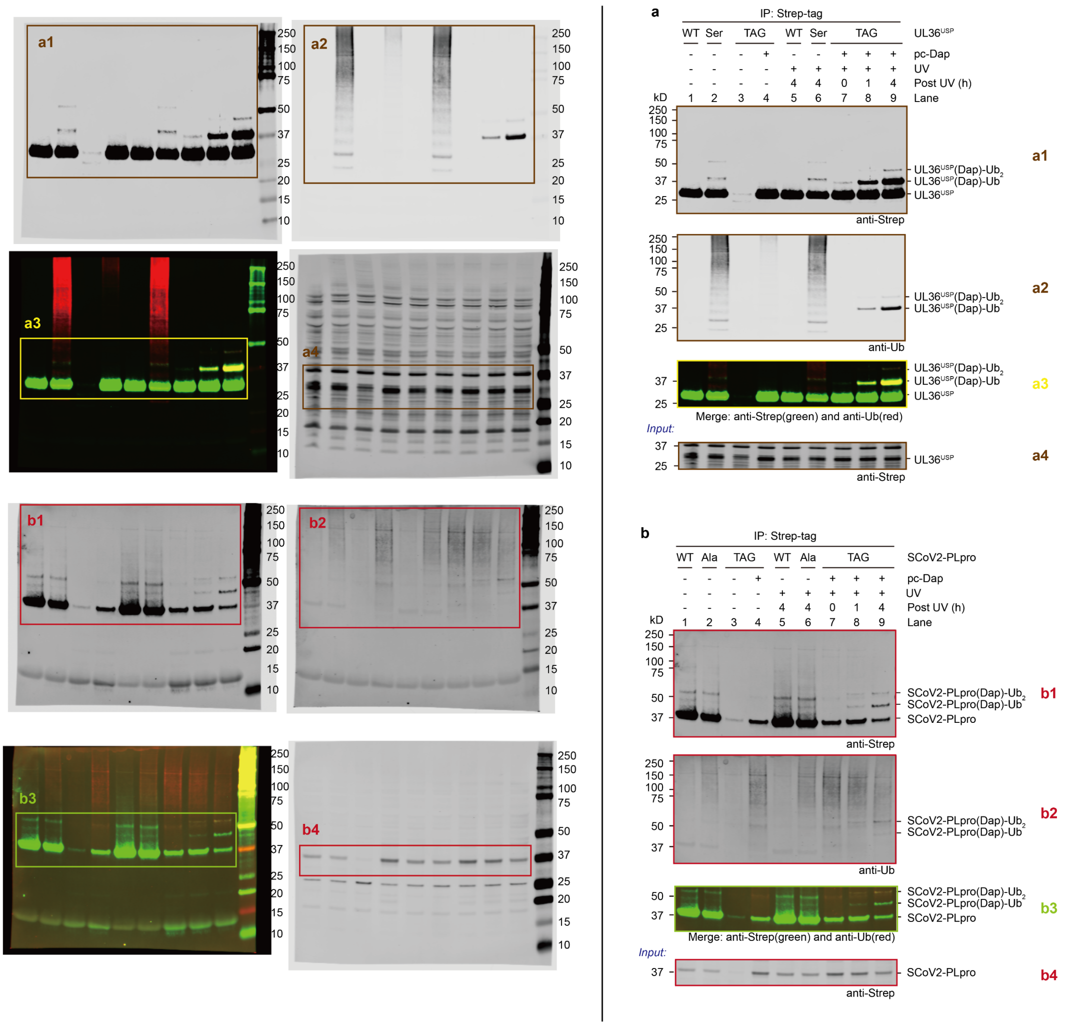

Supplementary Figure 1 (continued): Uncropped western blots for Extended Data Figure 5

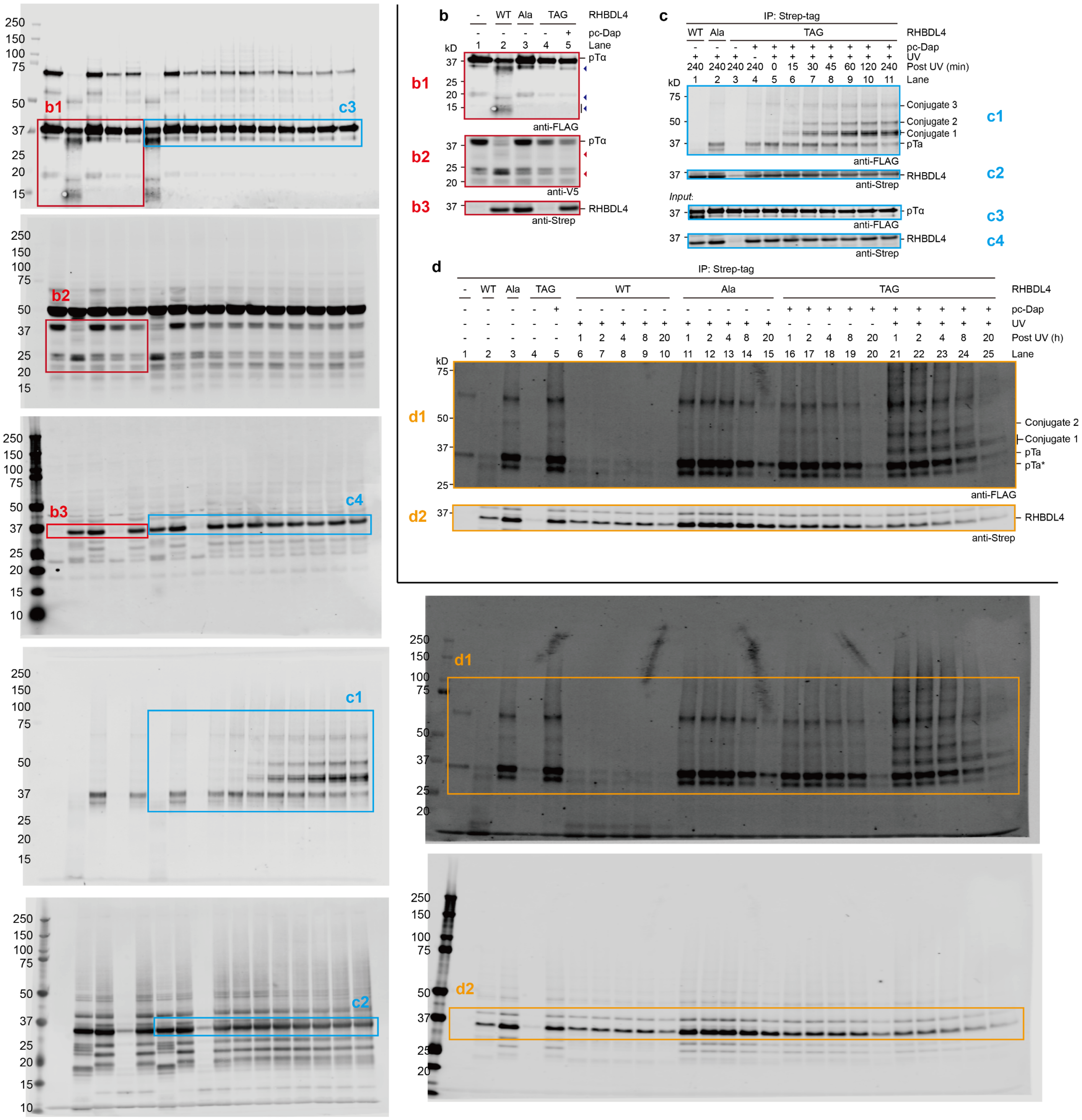

Supplementary Figure 1 (continued): Uncropped western blots for Extended Data Figure 6

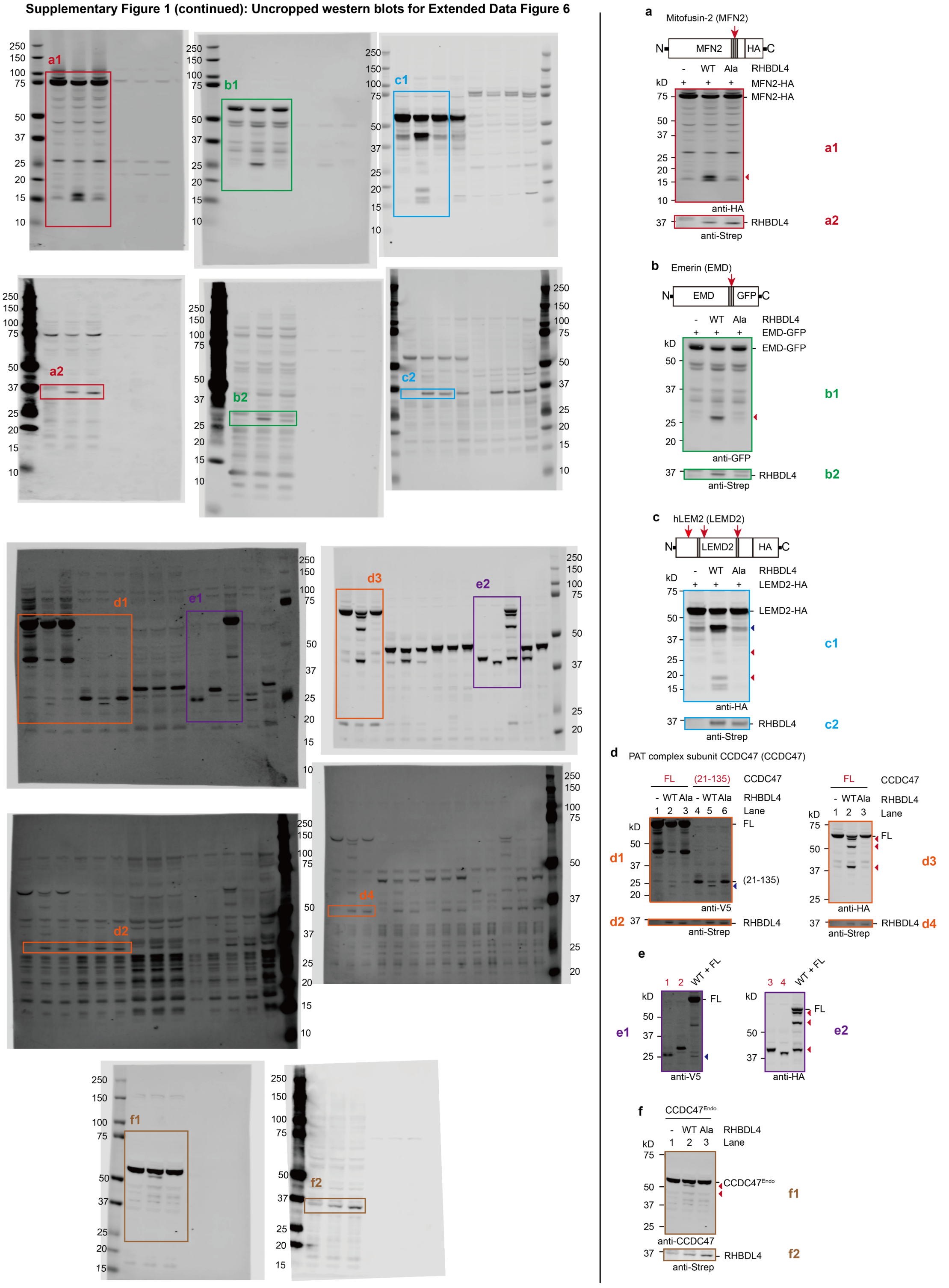

Supplementary Figure 1 (continued): Uncropped western blots for Extended Data Figure 7

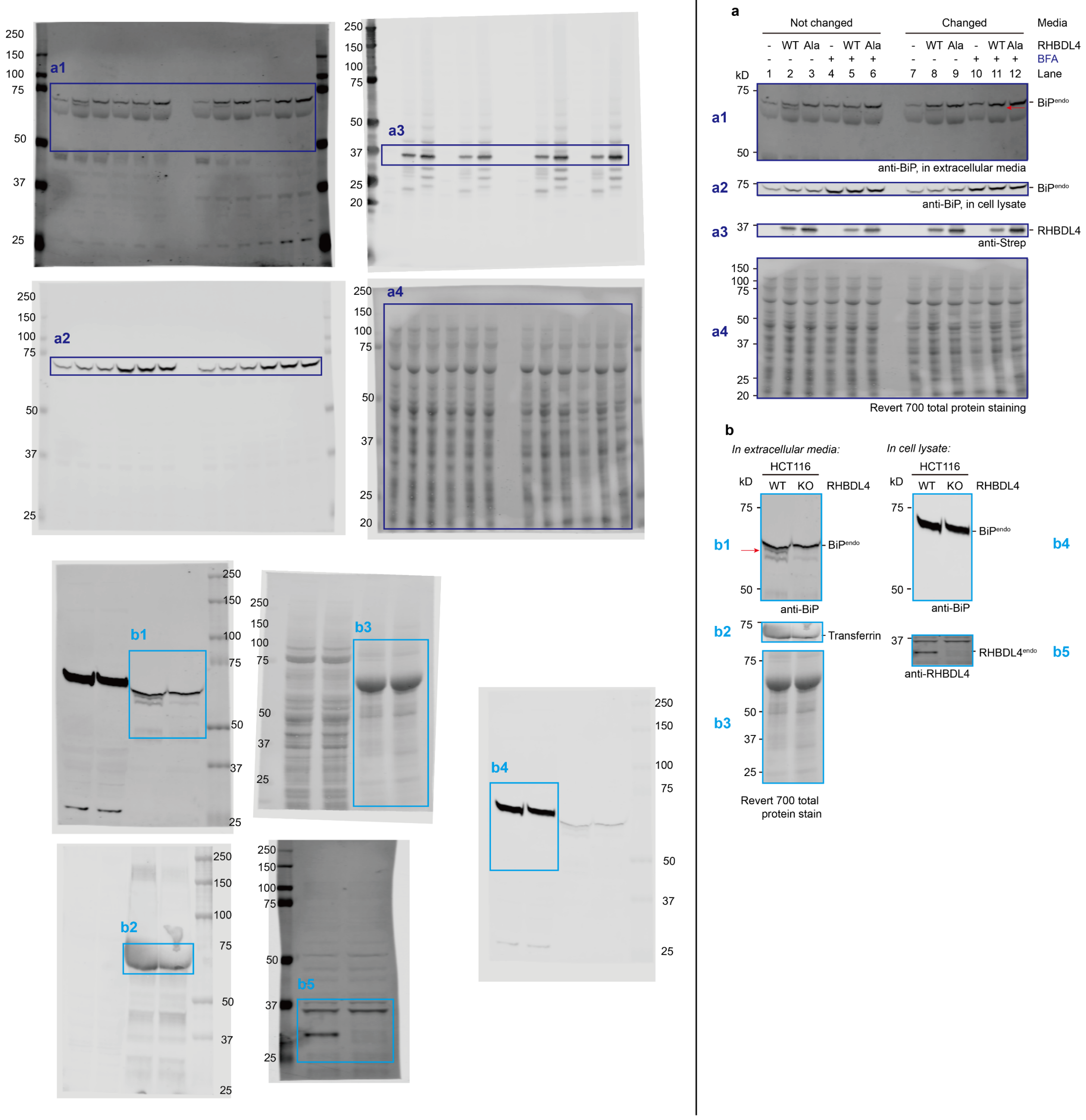

Supplementary Figure 1 (continued): Uncropped western blots for Extended Data Figure 8

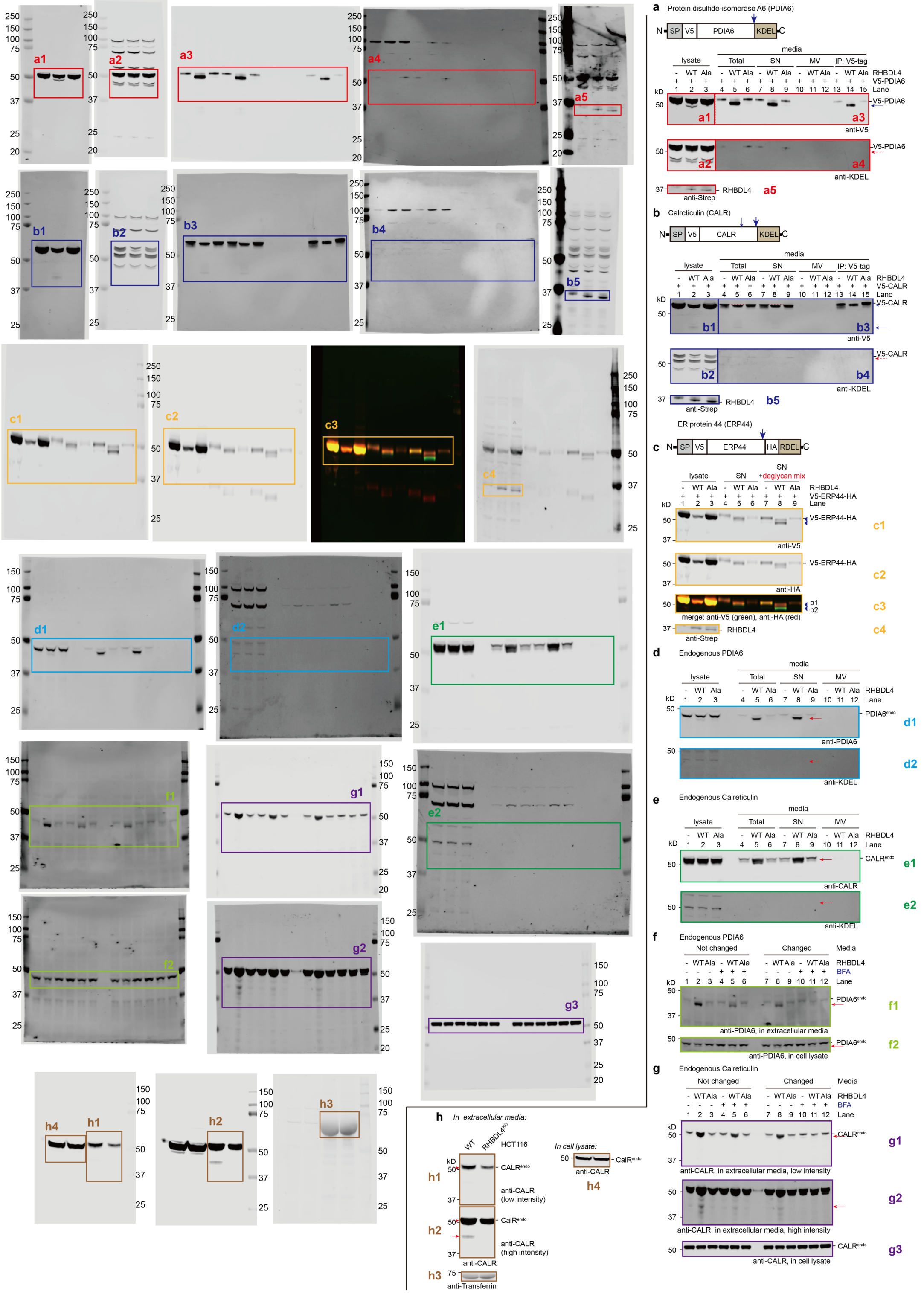

Supplementary Figure 1 (continued): Uncropped SDS-PAGE for Supplementary Figure 2

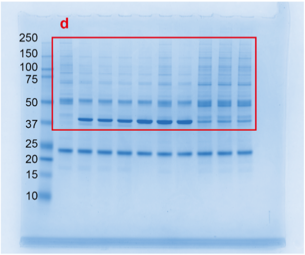

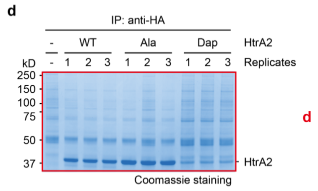

Supplementary Figure 1 (continued): Uncropped western blots for Supplementary Figure 3

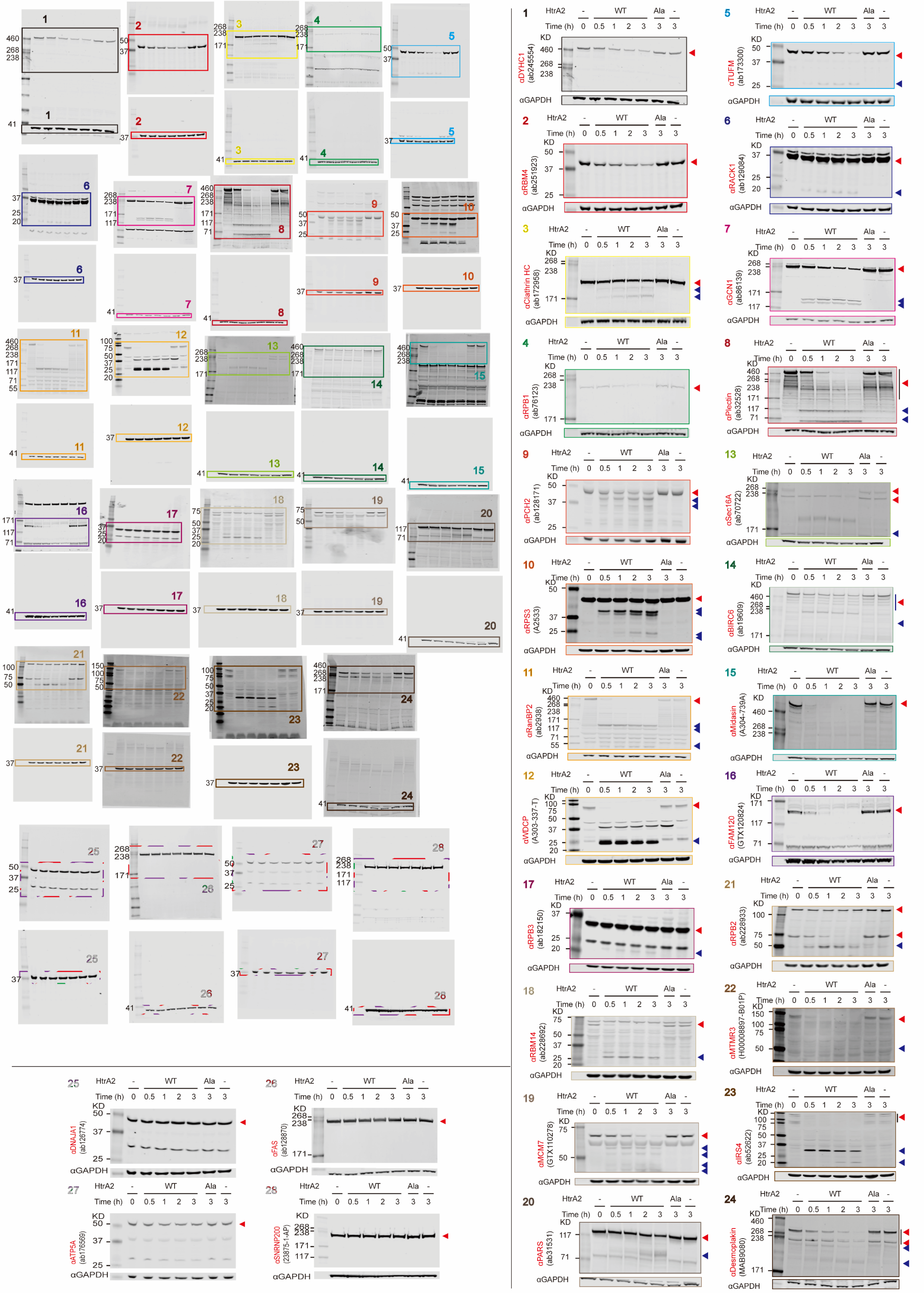

Supplementary Figure 1 (continued): Uncropped western blots for Supplementary Figure 6

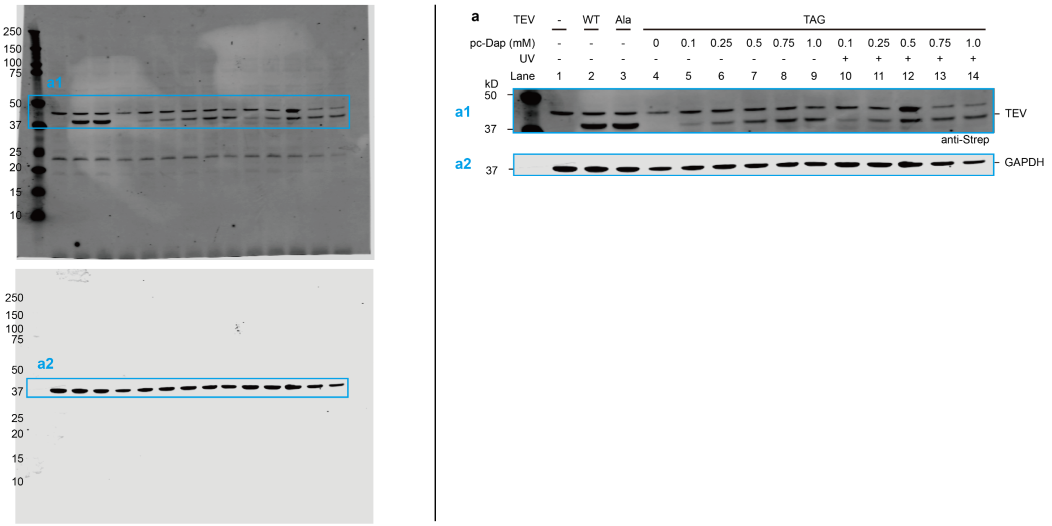

Supplementary Figure 1 (continued): Uncropped western blots for Supplementary Figure 9

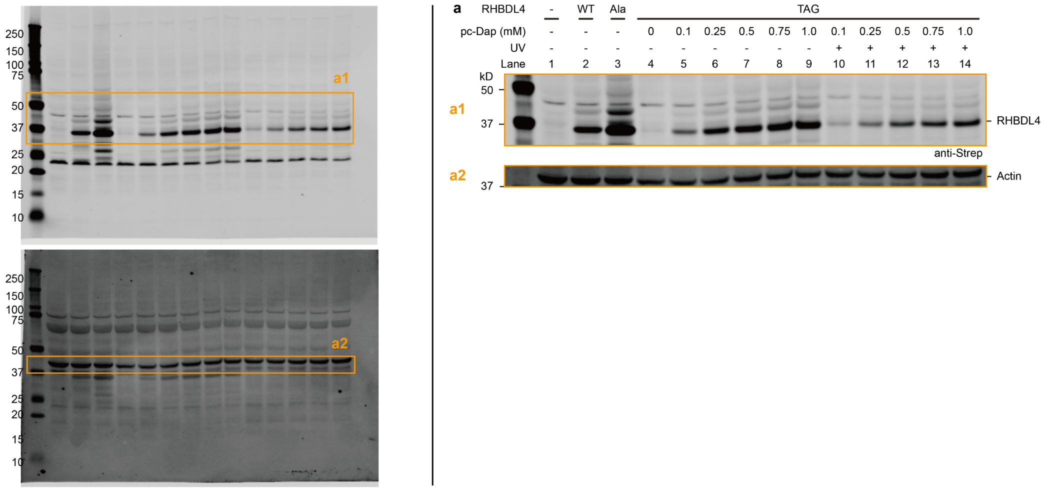

Supplementary Figure 1 (continued): Uncropped western blots for Supplementary Figure 10

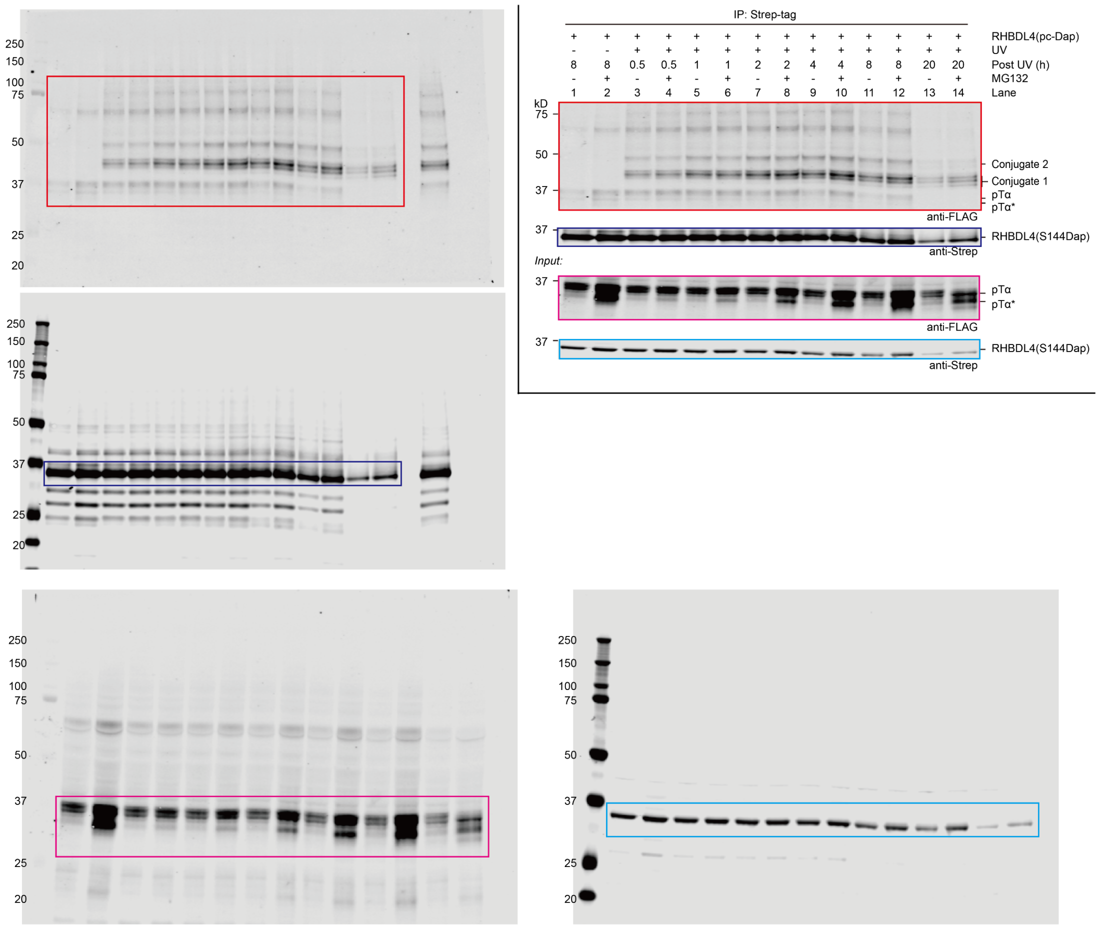

Supplementary Figure 1 (continued): Uncropped western blots for Supplementary Figure 11

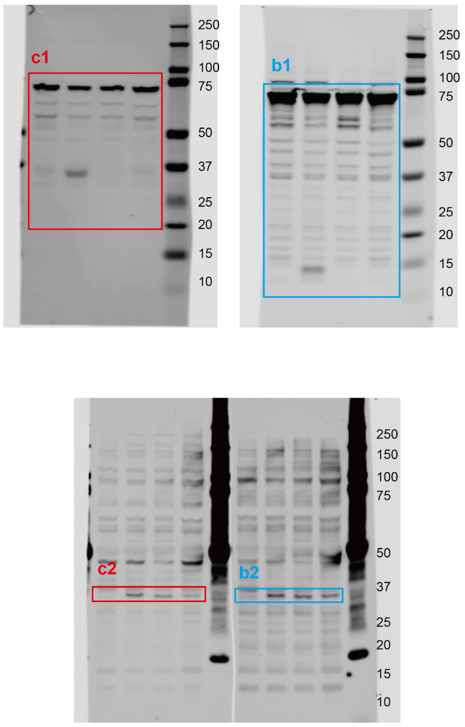

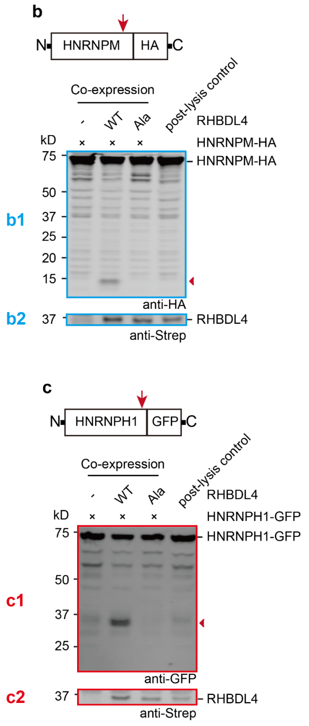

Supplementary Figure 1 (continued): Uncropped western blots for Supplementary Figure 12

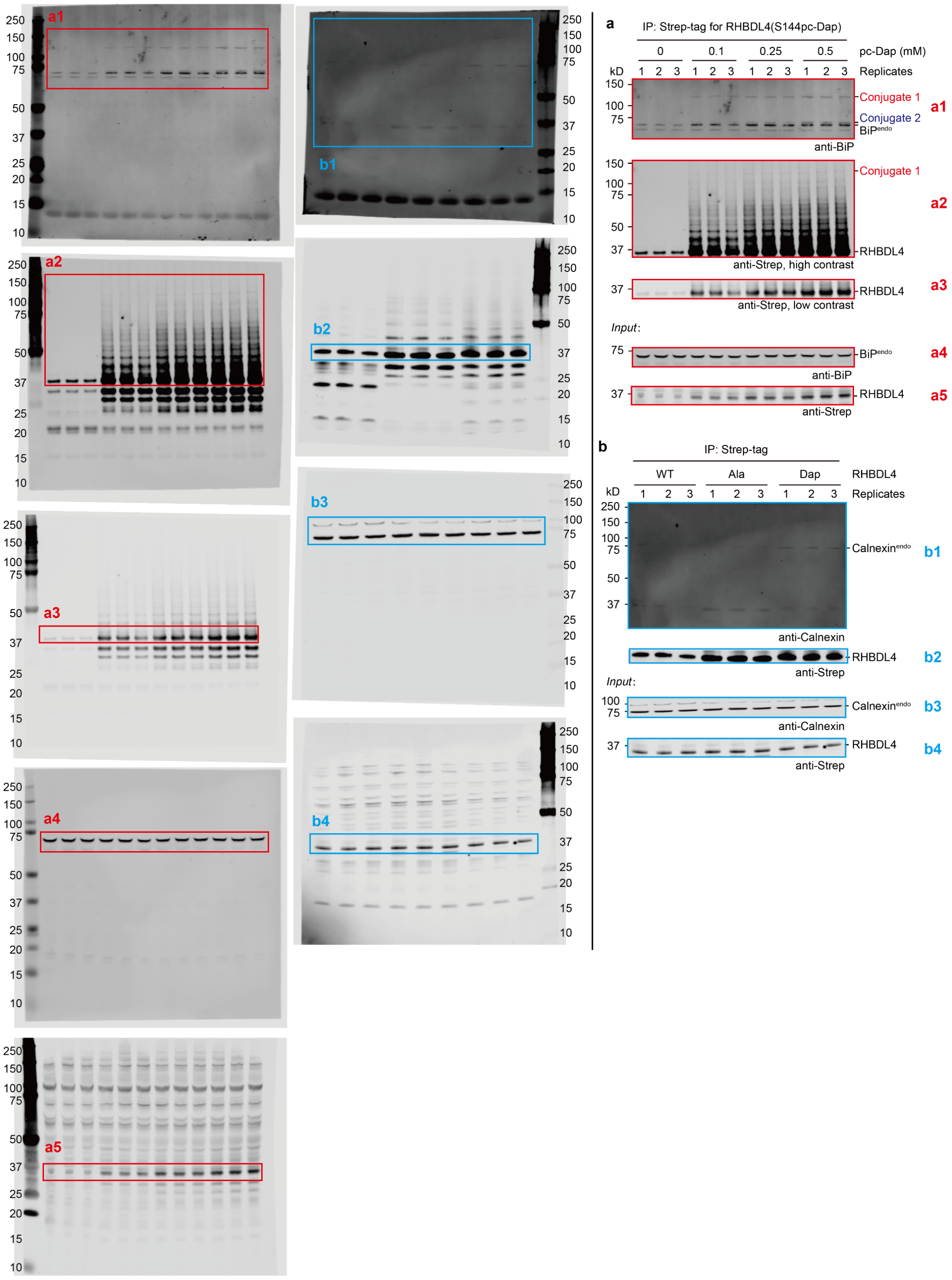

Supplementary Figure 1 (continued): Uncropped western blots for Supplementary Figure 13

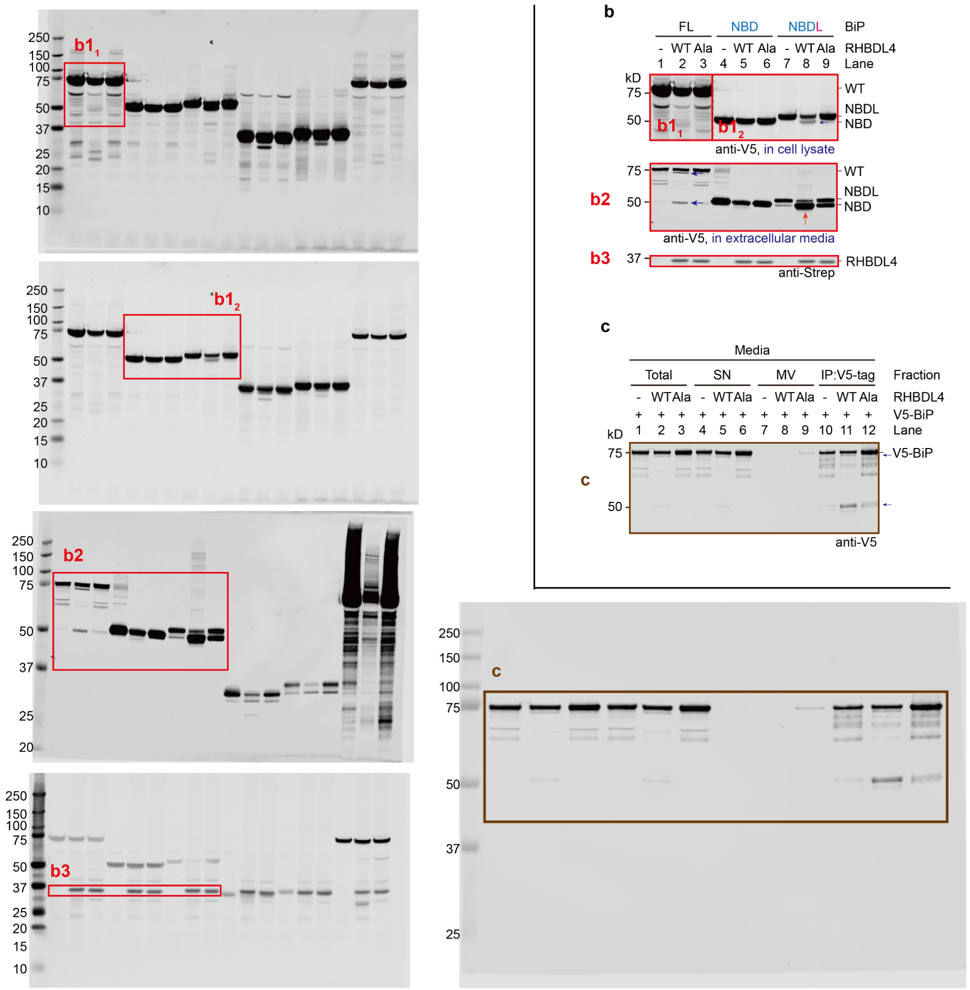

Supplementary Figure 1 (continued): Uncropped western blots for Supplementary Figure 14

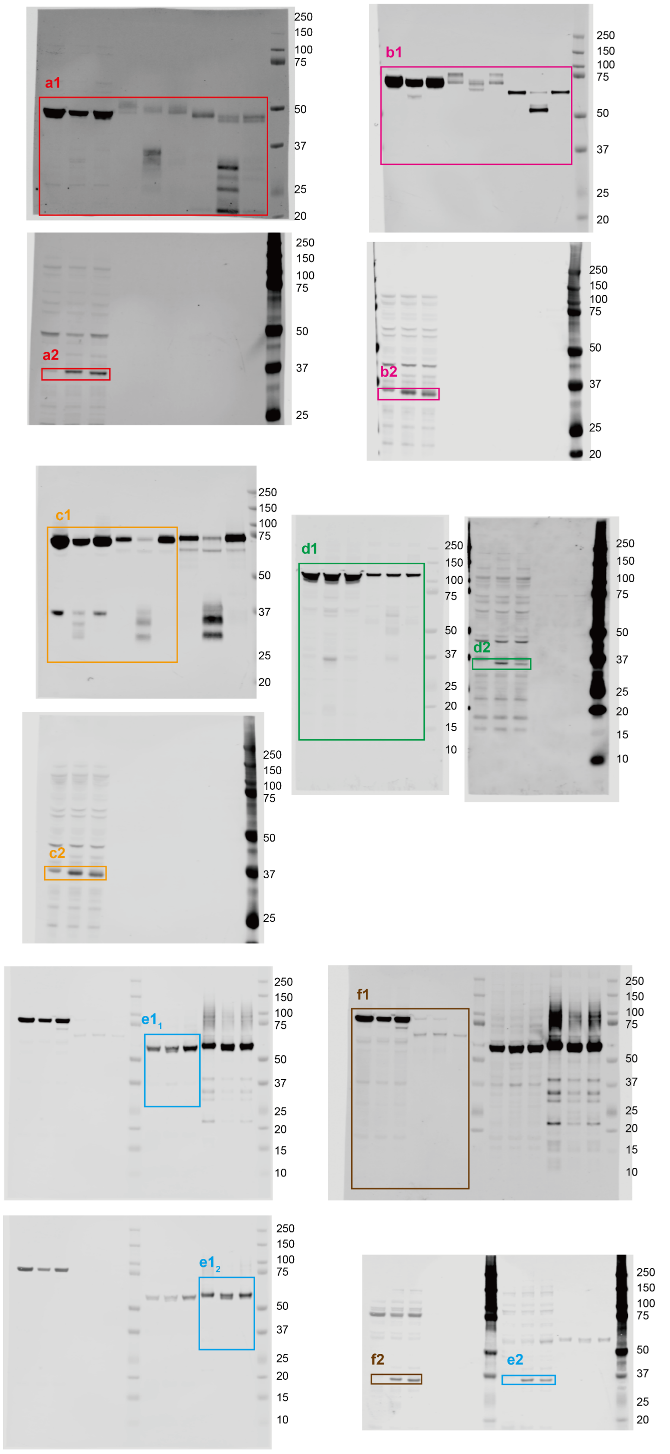

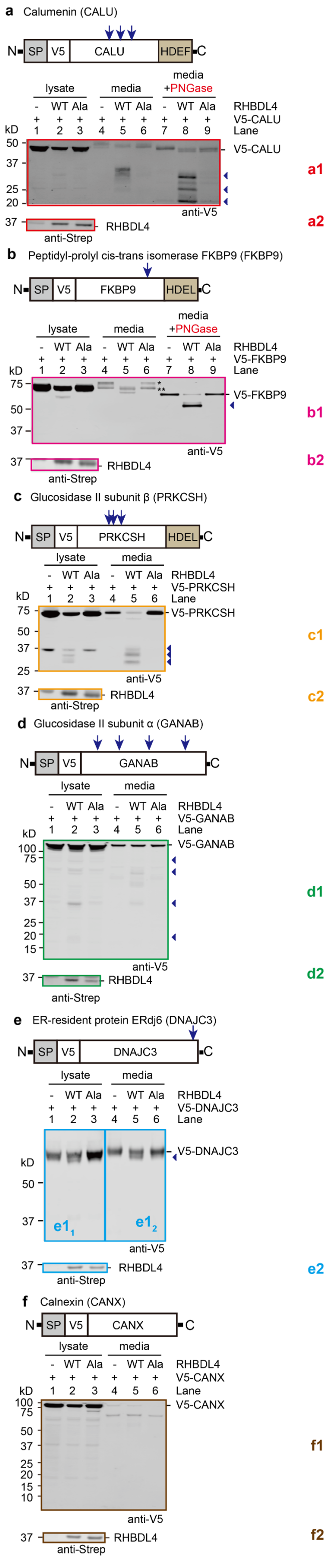

Supplementary Figure 1 (continued): Uncropped western blots for Supplementary Figure 15

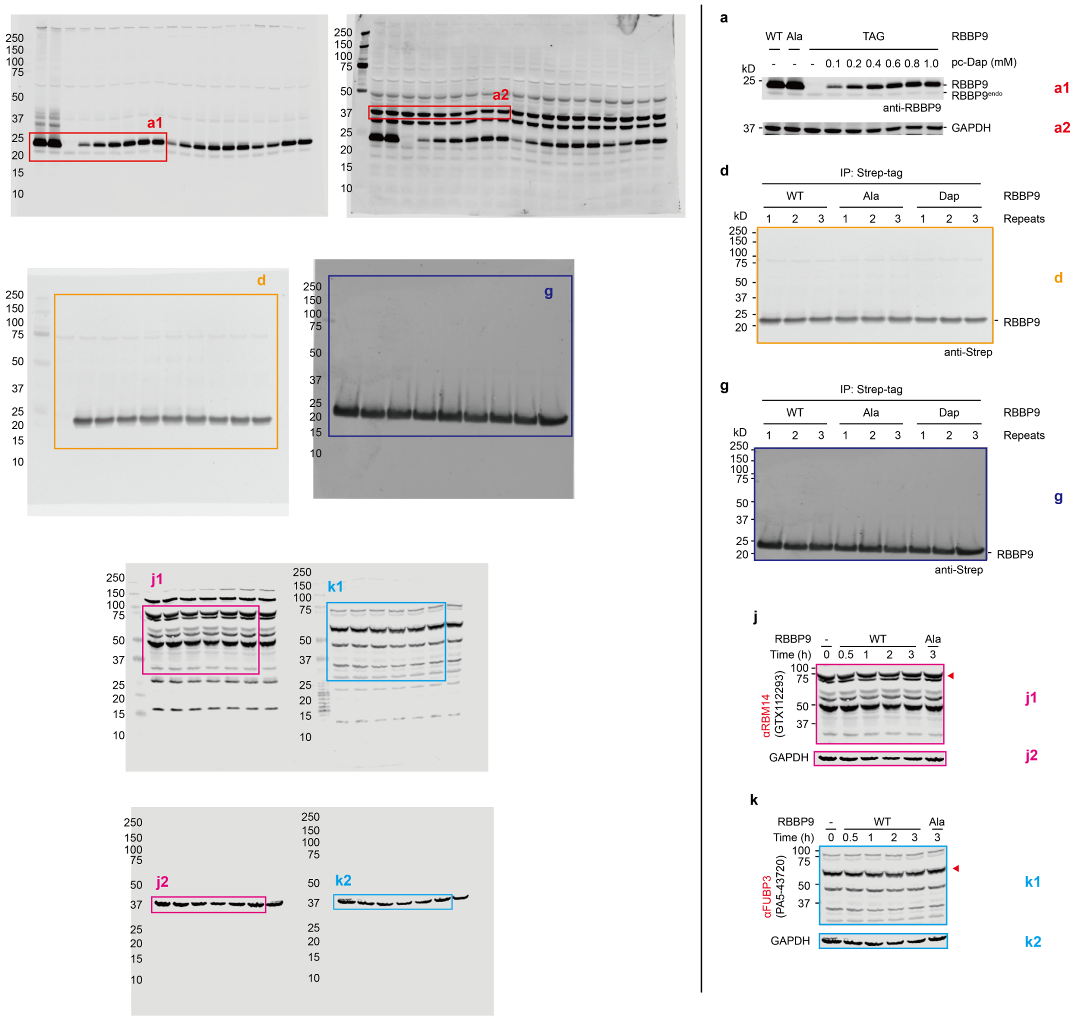

Supplementary Figure 1 (continued): Uncropped western blots for Supplementary Figure 16

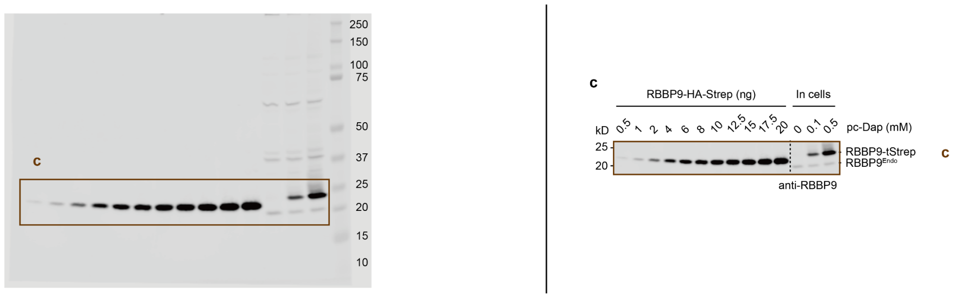

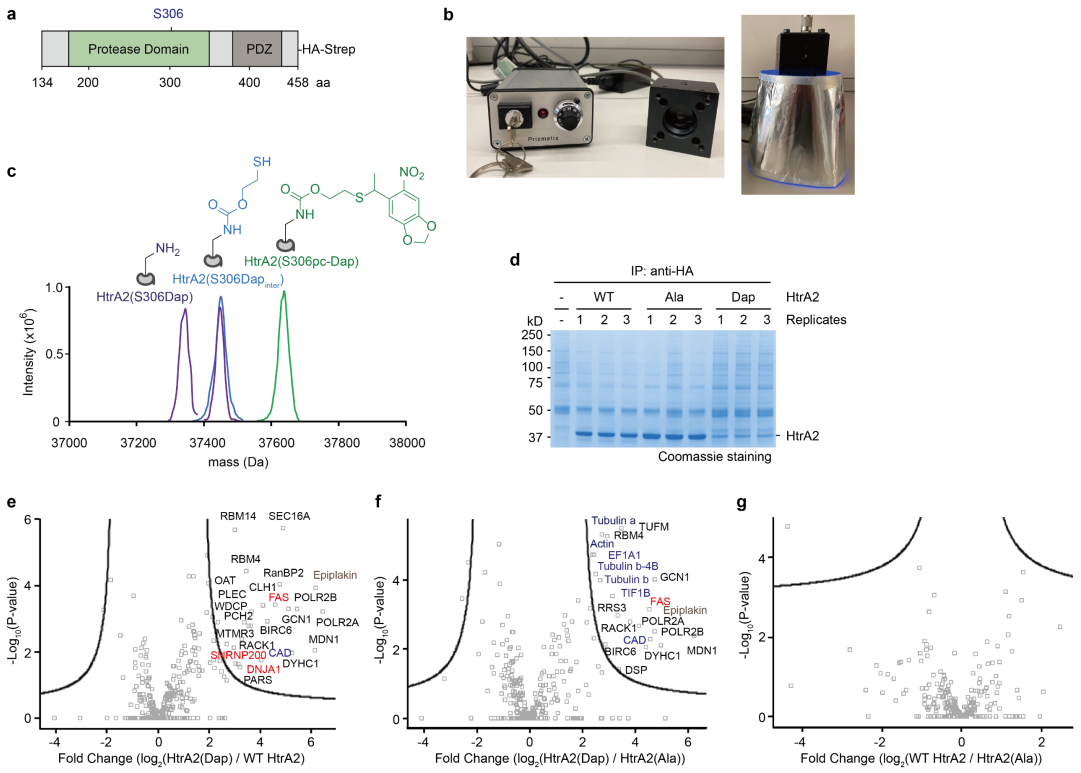

Supplementary Figure 2. Characterization of HtrA2(S306Dap) and proteomic analysis of proteins enriched in WT HtrA2, HtrA2(S306A) and HtrA2(S306Dap) samples. (a) Schematic representation of mature HtrA2 with a C-terminal HA-Strep-tag. Catalytic Ser306 (S306) is indicated. (b) The equipment used for illuminating pc-Dap containing proteins in buffer. For equipment specifications, see **Methods**. (c) Deprotection of HtrA2(S306pc-Dap) in buffer followed by ESI-MS characterization. Green trace, purified [HtrA2(S306pc-Dap)-Met]: expected 37644.5 Da, observed 37644.6 Da. Blue trace, [HtrA2(S306Dap_{inter})-Met]: expected 37450.6 Da, observed 37448.6 Da. Purple trace, 50% of the intermediate was converted to HtrA2(S306Dap) after incubation in Tris buffer (pH 8.0, 5 mM DTT) for 24 h at 37 °C, for [HtrA2(S306Dap)-Met]: expected 37346.5 Da, observed 37345.5 Da. (d) Equal amount of HtrA2 variants were incubated with Expi293 cell lysate at 30 °C for 3 h. HtrA2 variants were enriched by anti-HA beads, and proteins eluted from anti-HA beads were analysed by SDS-PAGE and visualized by Coomassie staining. The experiment was performed in biological triplicate. For the 265 proteins found in both the HtrA2(S306Dap) elution and either the WT HtrA2 or HtrA2(S306A) elution, label-free quantification (LFQ) analysis was performed to assess the relative abundance of proteins. (e) (HtrA2(Dap) / WT HtrA2), (f) (HtrA2(Dap) / HtrA2(Ala)) and (g) (WT HtrA2 / HtrA2(Ala)) represented the ratios of the LFQ value. The black line represents the cut-off curve for significance ($S_0 = 1$, FDR < 0.01). Protein identities are annotated in each graph (black: substrates, red: non-substrates, and blue: previously reported substrates). Each data point is calculated in Perseus using n = 4 for each HtrA2 variant. The substrate candidate details are summarized in Supplementary Tables 2 and 3.

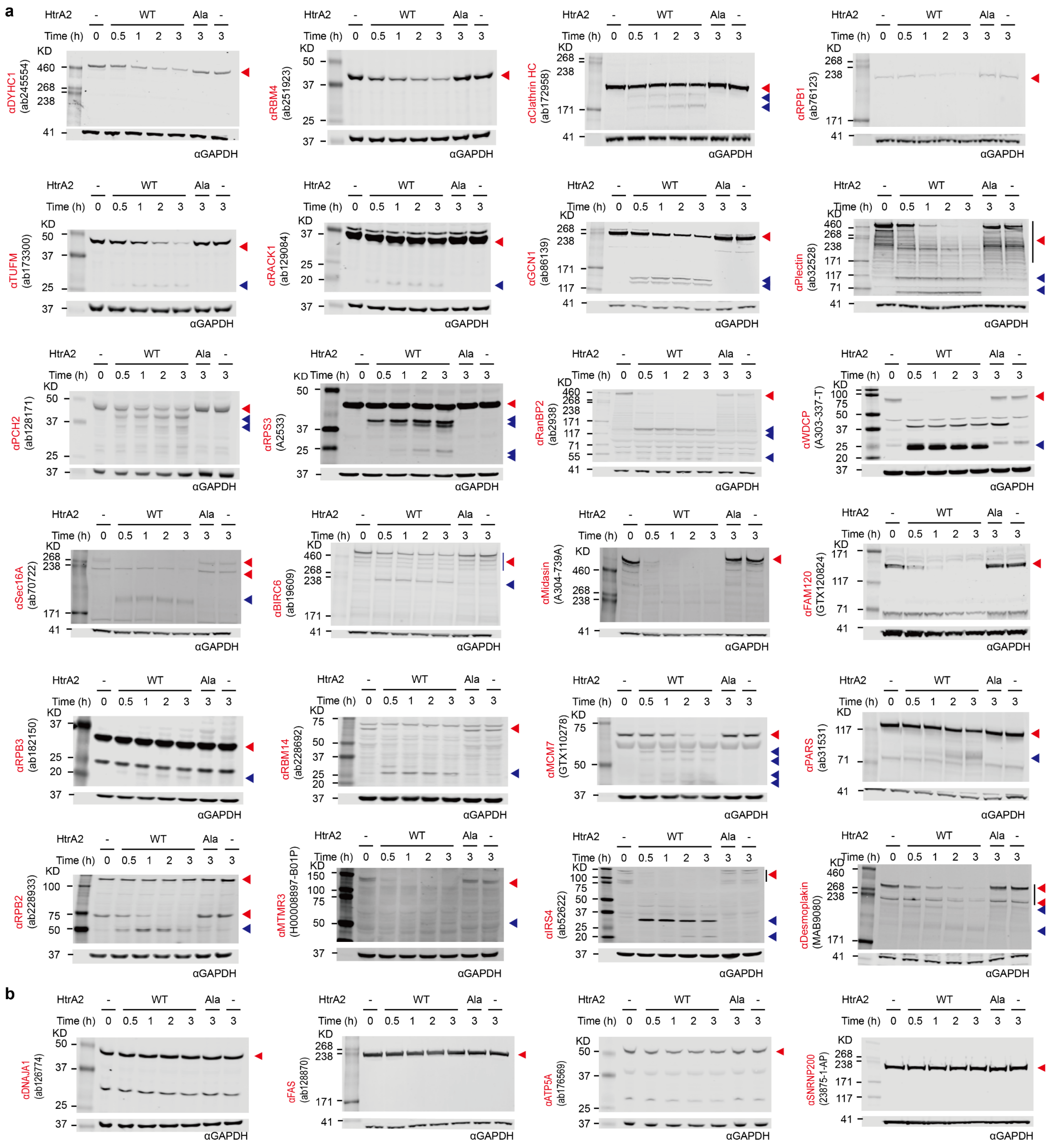

Supplementary Figure 3. Validation of potential HtrA2 substrates enriched by LFQ analysis. WB analysis of Expi293 cell lysate treated with 1 μM WT HtrA2 or HtrA2(S306A) for the indicated time points. (a) Candidates showed WT HtrA2-dependent generation of proteolytic fragments and/or decreasing intensity of target full-length protein bands. (b) Candidates did not show detectable WT HtrA2-dependent proteolysis. Red triangles denote the full-length protein, and blue triangles indicate WT HtrA2-dependent proteolytic fragments. Each candidate was validated by two biological replicates with similar results.

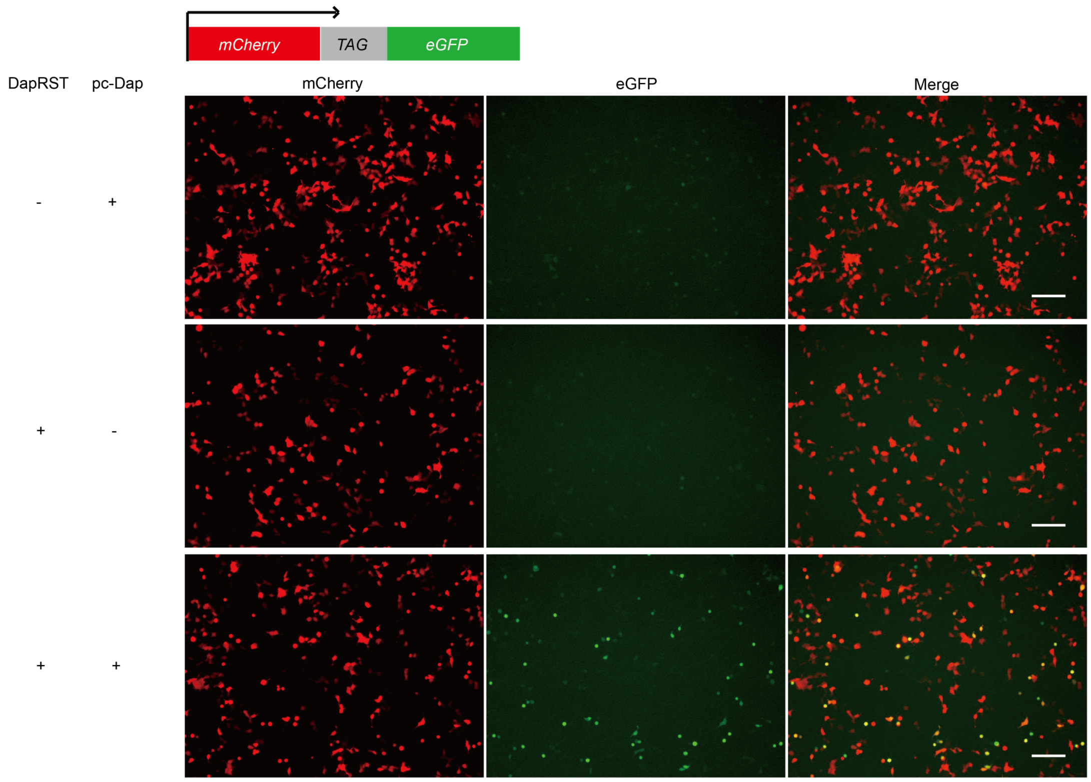

Supplementary Figure 4. Fluorescence microscopy analysis of pc-Dap incorporation in HEK293T cells. mCherry-TAG-eGFP-containing plasmid was co-transfected with an empty or DapRS/tRNA$_{CUA}$ (DapRST) pair-containing plasmid into HEK293T cells. 30 min after transfection, 1 mM pc-Dap was added (+) or not (-) as indicated into the culture. EGFP expression was dependent on the presence of both pc-Dap and the DapRS/tRNA$_{CUA}$ pair. Scale bar: 200 µm. The fluorescence microscopy analysis was performed in three biological replicates with similar results.

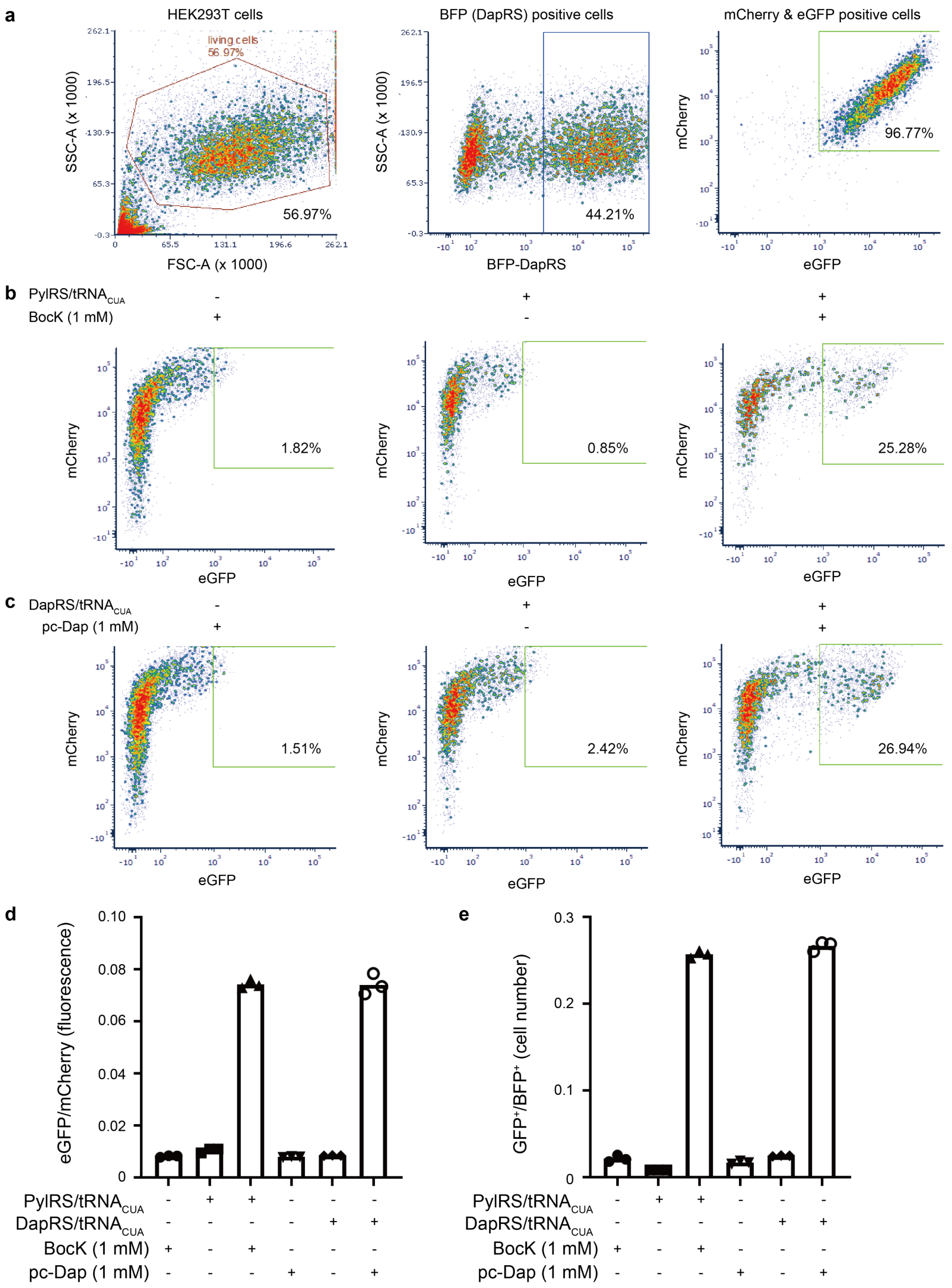

Supplementary Figure 5. Flow cytometry analysis of pc-Dap incorporation in HEK293T cells. (a) The gating for HEK293T, BFP positive (BFP is fused to the N-terminus of DapRS to indicate its expression), and mCherry and eGFP positive cells. (b) Incorporation of N$^{\varepsilon}$-*tert*-butyloxycarbonyl-L-lysine (BocK) into mCherry-TAG-eGFP was dependent on the presence of both BocK and the PylRS/tRNA$_{CUA}$ pair. (c) Incorporation of pc-Dap into mCherry-TAG-eGFP was dependent on the presence of both pc-Dap and the DapRS/tRNA$_{CUA}$ pair. Non-canonical amino acid (ncAA) incorporation efficiency was quantified by (d) eGFP / mCherry and (e) eGFP / BFP. The incorporation efficiency for pc-Dap with the DapRS/tRNA$_{CUA}$ pair was comparable to that of BocK with the PylRS/tRNA$_{CUA}$ pair, which is commonly used as a benchmark for ncAA incorporation in mammalian cells. (d) and (e) were generated using n = 3 independent measurements. The bars represent the means of three measurements.

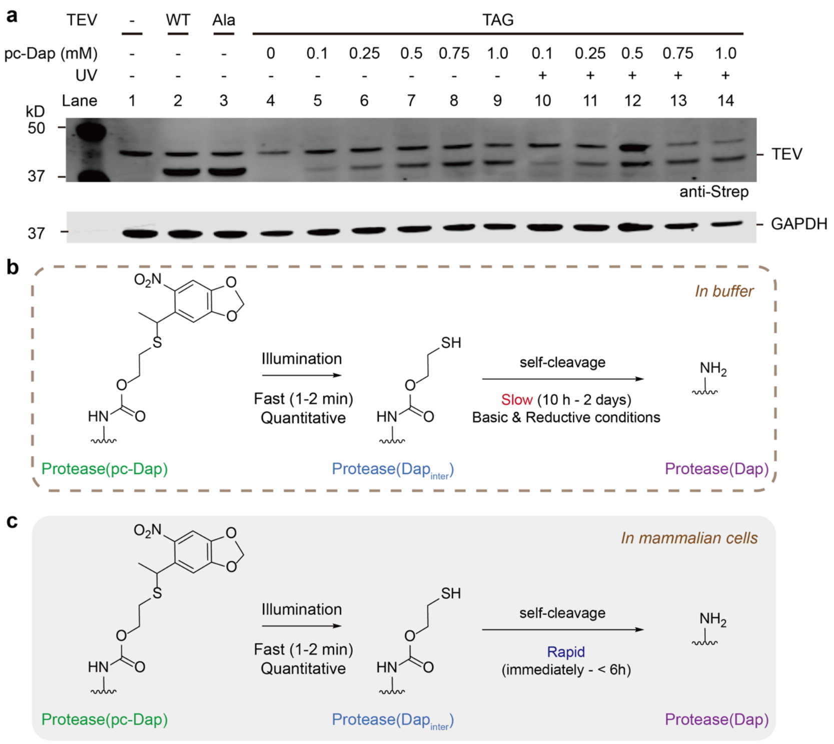

Supplementary Figure 6. Expression and conversion of TEV(C151pc-Dap) to TEV(C151Dap) in HEK293T cells. (a) DapRS/tRNA$_{CUA}$-containing plasmid was co-transfected with WT TEV, TEV(C151A), or TEV(C151TAG) plasmid in HEK293T cells. 30 min after transfection, pc-Dap was added at indicated concentrations to test the pc-Dap incorporation efficiency. Anti-Strep immunoblot showed that the expression of TEV(C151pc-Dap) increases as a function of pc-Dap concentration in the medium. TEV(C151Dap) was stable in HEK293T cells, as the intensity of anti-Strep bands did not decrease 12 h after illumination compared to samples prior to UV irradiation (lanes 5 vs 10; 6 vs 11; 7 vs 12; 8 vs 13; 9 vs 14). GAPDH: loading control. The experiments were performed in two biological replicates with similar results. (b) A two-step deprotection mechanism for conversion of Protease(pc-Dap) to Protease(Dap) in buffer. The removal of photocaged group (365 nm, 4 mW/cm^2) is complete within 1-2 min. However, the self-cleavage step requires hours to days at basic (pH 8.0) and reductive (e.g. 5 mM DTT) conditions to generate fully deprotected Protease(Dap). (c) Conversion of Protease(pc-Dap) to Protease(Dap) by directly illuminating mammalian cells. The removal of photocaged group inside mammalian cells by illumination (365 nm, 4 mW/cm^2) is efficient and quantitative. The second self-cleavage step inside cells is rapid as demonstrated by converting TEV(C151pc-Dap) to TEV(C151Dap).

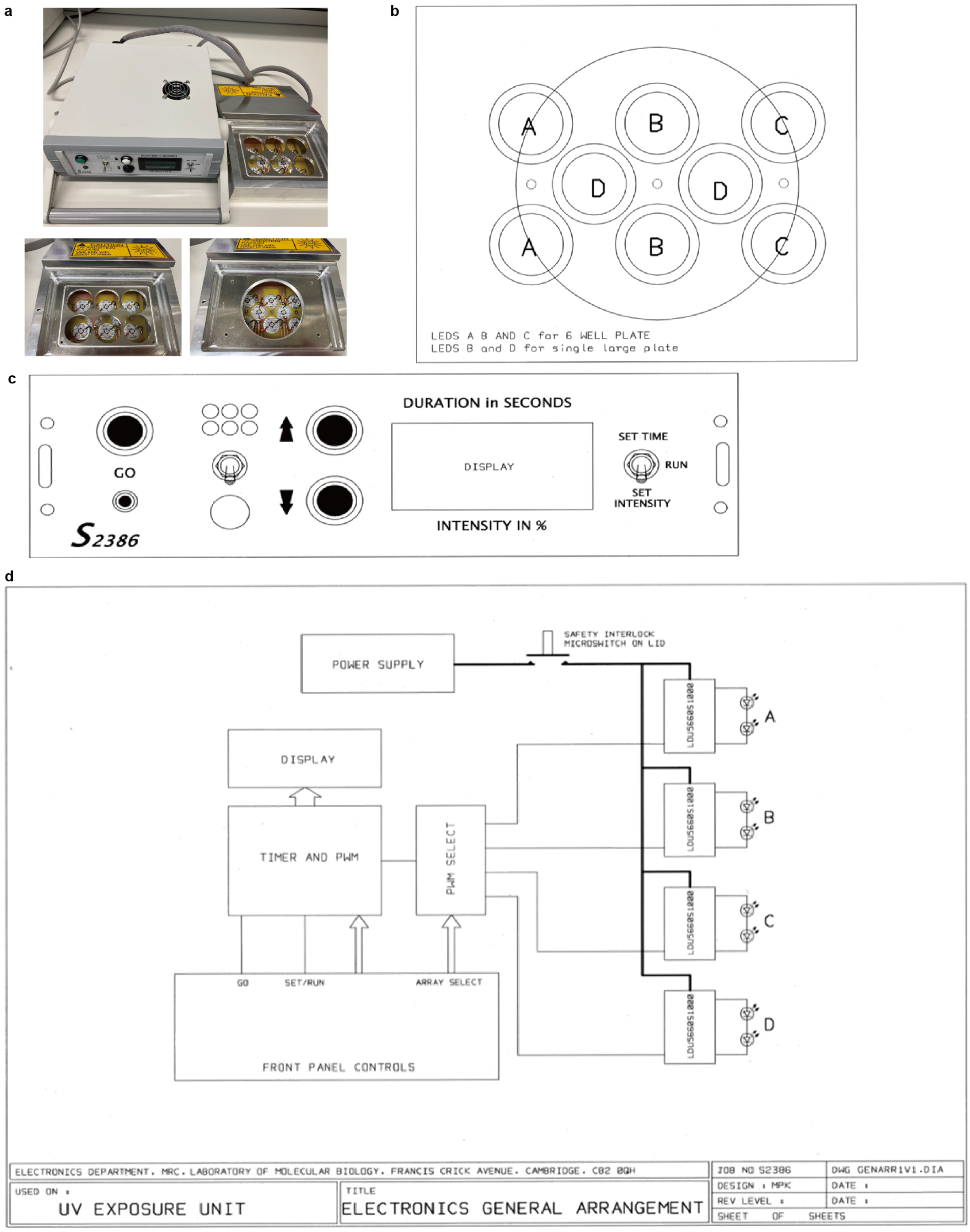

Supplementary Figure 7. The equipment for illuminating adherent mammalian cells. (a) The equipment used for illuminating adherent mammalian cells. (b) The arrangement of LED emitters for a 6 well cell culture plate (LEDs A, B and C) and a 10 cm petri dish (LEDs B and D). (c) The control panel for the time and intensity of illumination. (d) The electronics general arrangement.

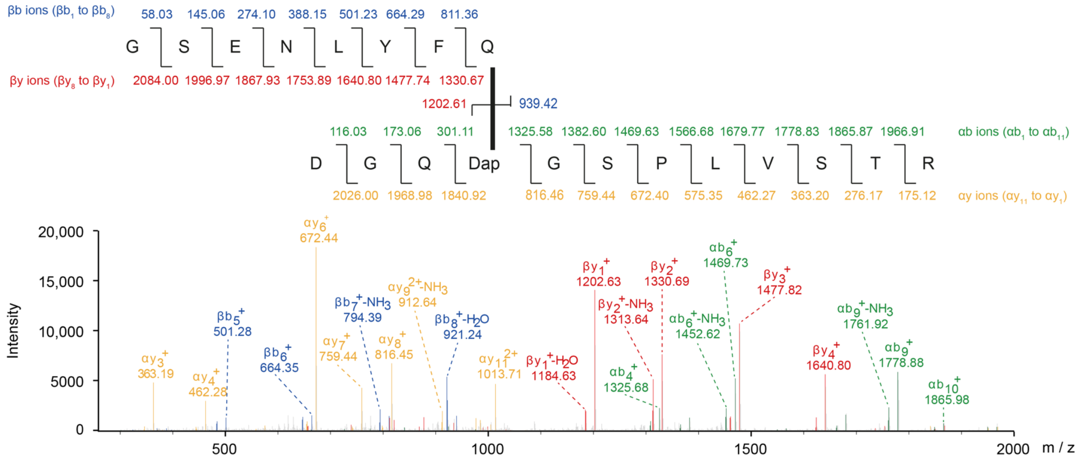

Supplementary Figure 8. LC-MS/MS characterization of TEV(Dap)-GFP conjugate formed in HEK293T cells. The gel slice of TEV(Dap)-GFP conjugate (lane 9, Fig. 2c) was trypsinized and analyzed by LC-MS/MS. The branched fragment of TEV(Dap)-GFP conjugate was confirmed at C151Dap by LC-MS/MS. Top, the sequence of the branched peptide subject to fragmentation. Fragmentation of the TEV(C151Dap)-derived chain (DGQ(Dap)GSPLVSTR) is predicted to form a series of y ions (yellow) and b ions (green); the ions from this chain are labelled as ‘α’. Fragmentation of the substrate chain (GSENLYFQ) is predicted to generate a series of y ions (red) and b ions (blue); the ions from this chain are labelled as ‘β’. Bottom, MS/MS spectra with peak assignments. Ions in the α-chain were assigned by modifying Dap with the substrate chain mass. The mass-spectrometry analysis was performed once.

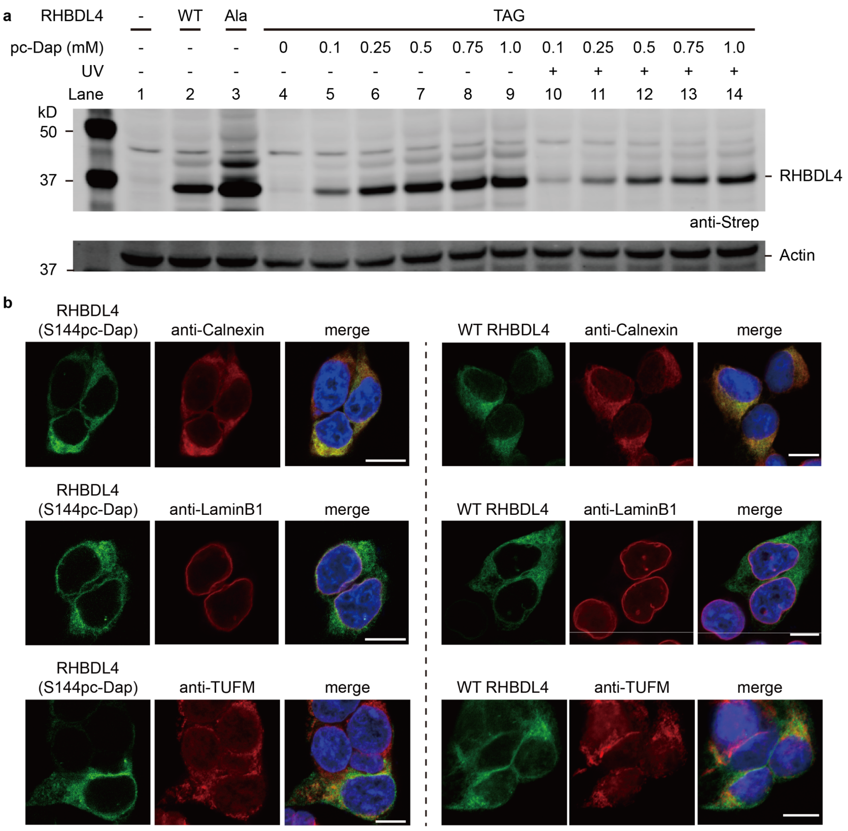

Supplementary Figure 9. Expression of RHBDL4(S144pc-Dap) in HEK293T cells. (a) DapRS/tRNA$_{CUA}$ containing plasmid and WT RHBDL4, RHBDL4(S144A) or RHBDL4(S144TAG) plasmid were co-transfected into HEK293T cells. Pc-Dap was added at indicated concentrations to test the efficiency of full-length RHBDL4(S144pc-Dap) production. The expression of RHBDL4(S144pc-Dap) increases as a function of pc-Dap concentration in the medium. The expression of RHBDL4(S144pc-Dap) in the presence of 0.5 mM pc-Dap was the same as WT RHBDL4 according to the band intensities (lanes 2 and 7), while RHBDL4(S144A) was about 3.5-fold over WT RHBDL4 (lane 3 vs 2 or 7). Thus, we reduced the amount of RHBDL4(S144A) plasmid to 30% of WT RHBDL4 or RHBDL4(S144TAG) during transfection. More than 50% of RHBDL4(S144Dap) was degraded 12 h after illumination in cells (Lane 5 vs 10; 6 vs 11; 7 vs 12; 8 vs 13; 9 vs 14). Actin: loading control. (b) Immunofluorescence (IF) analysis of RHBDL4(S144pc-Dap). C-terminal GFP-tagged WT RHBDL4 or RHBDL4(S144pc-Dap) was expressed in HEK293T cells for 40 h. 0.5 mM pc-Dap was used for producing full-length RHBDL4(S144pc-Dap)-GFP. Cells were fixed and stained with anti-Calnexin (the ER marker) or anti-LaminB1 (the nuclear membrane marker) or anti-TUFM (the mitochondrion marker). The overlap of anti-Calnexin (red) and RHBDL4(S144pc-Dap)-GFP (green) signals confirmed that RHBDL4(S144pc-Dap) localized at the ER. In addition, RHBDL4(S144pc-Dap)-GFP (green) also overlapped with anti-LaminB1 (red) signals, indicating that RHBDL4 (S144pc-Dap) could also localize at the nuclear membrane. In contrast, RHBDL4(S144pc-Dap)-GFP (green) did not overlap with anti-TUFM (red) signals, demonstrating that RHBDL4(S144pc-Dap) was not primarily expressed in mitochondria. WT RHBDL4 showed a comparable localization to RHBDL4(S144pc-Dap) in IF analysis. Scale bar: 10 μm. (a) and (b) were performed in two biological replicates with similar results.

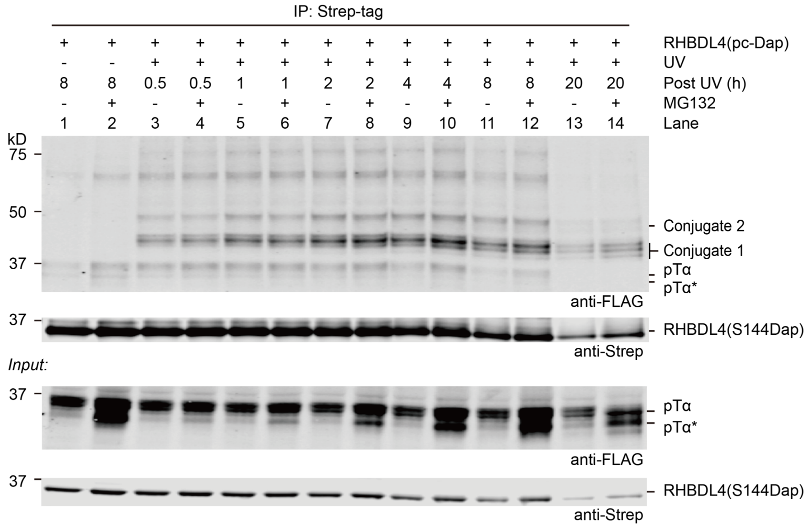

Supplementary Figure 10. Addition of a proteasome inhibitor retards the degradation of RHBDL4(Dap)-pTα conjugates. RHBDL4(S144TAG) and pTα were co-transfected in Expi293 cells in the presence of 0.5 mM pc-Dap and incubated at 37 °C for 40 h. Proteasome inhibitor MG132 was added immediately after illumination as indicated (lanes 4, 6, 8, 10, 12 and 14). Cells without illumination were collected as controls (lanes 1 and 2). The total lysate (input) and proteins eluted from beads after Strep-tag pulldown were analyzed by anti-FLAG (for pTα and conjugates) and anti-Strep (for RHBDL4) antibodies. pTα*, the deglycosylated form of pTα, was stabilized by MG132 as reported[1]. MG132 treatment slowed down the degradation of RHBDL4(S144Dap) and protected conjugates from proteasome degradation at each indicated time point. The optimal conditions for trapping RHBDL4 substrates inside human cells would be 4h after UV illumination in the presence of MG132. The experiments were performed in two biological replicates with similar results.

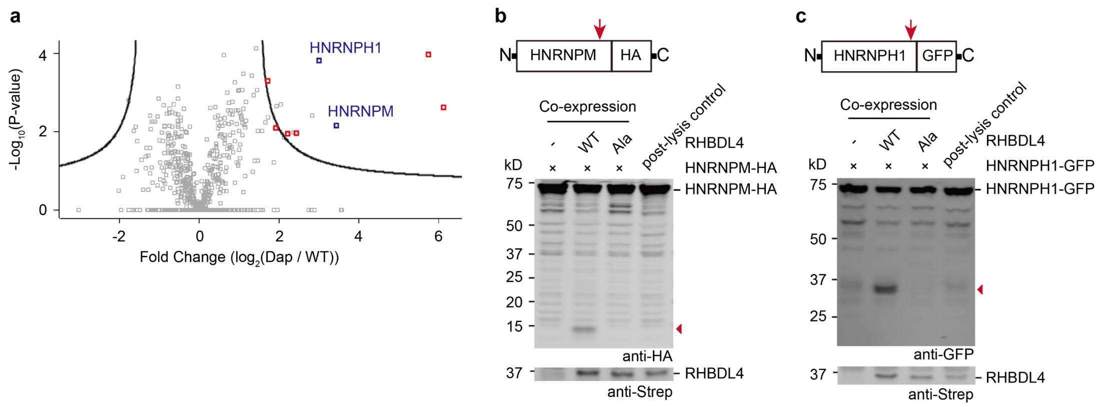

Supplementary Figure 11. Characterization of non-ER resident substrate candidates. (a) Volcano plot based on the LFQ values for the proteins identified in RHBDL4(S144Dap) and WT RHBDL4 samples. ER-resident protein candidates are labeled in red, and the two most enriched nuclear proteins (heterogeneous nuclear ribonucleoprotein M (gene name: HNRNPM) and heterogeneous nuclear ribonucleoprotein H (gene name: HNRNPH1)) are labeled in blue. RHBDL4 cleavage assays for (b) HNRNPM and (c) HNRNPH1. The putative cleavage sites are indicated by red arrows in the schematic representations. Post-lysis control was performed to test if the RHBDL4-mediated proteolysis happened during the process of cell lysis. An equal number of cells expressing only WT RHBDL4 and only the substrate candidate was mixed and lysed in the equal volume of RIPA buffer used for lysis of cells co-expressing both WT RHBDL4 and the substrate candidate. Both HNRNPM and HNRNPH1 were proteolyzed by WT RHBDL4 in their C-terminal region. Red triangles: C-terminal proteolytic fragments. A GFP-tag was used for better detection of the C-terminal proteolytic fragment of HNRNPH1. The experiments were repeated in three biological replicates with similar results.

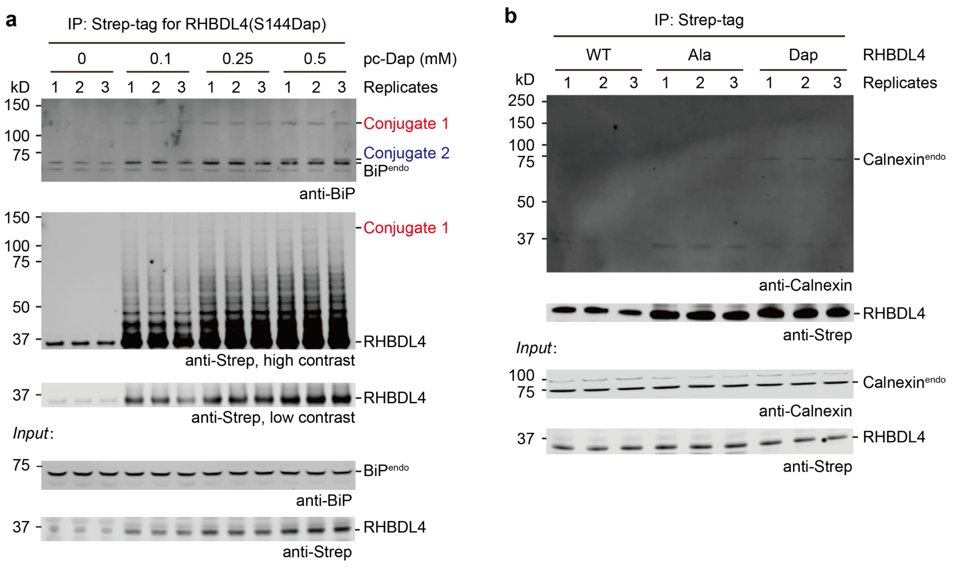

Supplementary Figure 12. RHBDL4(S144Dap) forms conjugates with endogenous BiP, but not Calnexin. (a) The level of RHBDL4(S144Dap) and extent of substrate trapping can be controlled by the concentration of pc-Dap added to cells. Immunoblotting analysis of RHBDL4(S144Dap), which was enriched from an equal number of cells expressing RHBDL4(S144pc-Dap) in the presence of 0, 0.1, 0.25 or 0.5 mM pc-Dap, after substrate trapping procedure. The level of RHBDL4(S144pc-Dap) increases as a function of pc-Dap concentration, as judged by anti-Strep blot before and after affinity enrichment. Anti-BiP and anti-Strep immunoblotting analysis demonstrates that the formation of conjugates is positively correlated to the expression level of RHBDL4(S144Dap) in cells. Input: detection of endogenous BiP (anti-BiP) and expression of RHBDL4 variants (anti-Strep) in cell lysate before IP. (b) Immunoblotting analysis of RHBDL4 variants enriched from an equal number of cells after substrate trapping procedure. RHBDL4(S144pc-Dap) was produced in the presence of 0.5 mM pc-Dap in the medium. No detectable conjugate was formed between RHBDL4(S144Dap) and endogenous Calnexin by an anti-Calnexin antibody, whose immunogen is within the N-terminal segment of Calnexin (Calnexin (1-273)). A very small amount of residual Calnexin was detected in all samples. Input: detection of endogenous Calnexin (anti-Calnexin) and the expression of RHBDL4 variants (anti-Strep) in cell lysate before IP. All experiments were performed in biological triplicate with similar results.

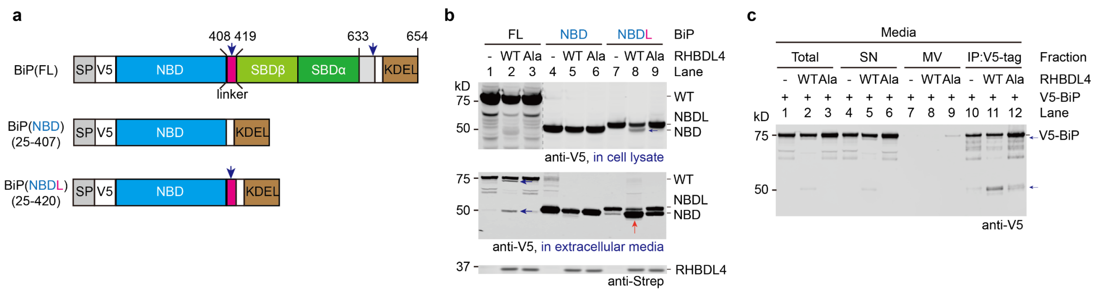

Supplementary Figure 13. Characterization of BiP cleavage by RHBDL4. (a) Schematic representations of full-length BiP (FL), BiP(NBD) (residue 25-407) and BiP(NBDL) (NBD plus interdomain linker, residue 25-420). The putative cleavage sites are indicated by blue arrows. SP: signal peptide; V5: V5-tag. (b) RHBDL4 cleavage assays for FL BiP, BiP(NBD) or BiP(NBDL). Anti-V5 immunoblotting showed that RHBDL4 cleaved FL BiP at the C-terminus and the interdomain linker (blue arrows, lane 2). This was further confirmed by the results that RHBDL4 cleaved BiP(NBDL) but not BiP(NBD) at the C-terminus (blue arrow, lane 8). In the presence of exogenously expressed WT RHBDL4, the N-terminal fragment was dramatically secreted (red arrow, lane 8). However, the fragment was also detected without exogenously expressed RHBDL4 (lane 7), indicating that endogenous RHBDL4 proteolyzed BiP(NBDL). Interestingly, the catalytically inactive RHBDL4(S144A) mutant promoted the secretion of N-terminal fragment (lane 9), suggesting that the endogenous RHBDL4 activity was activated in the presence of exogenously expressed catalytically inactive mutant. (c) WT RHBDL4 dependent proteolytic fragments were detected in total media and the SN, but not in MVs. In addition, anti-V5 agarose beads were able to pulldown the proteolytic fragments in the media, further confirming that these fragments were not in vesicles. (b) and (c) was performed in two biological replicates with similar results.

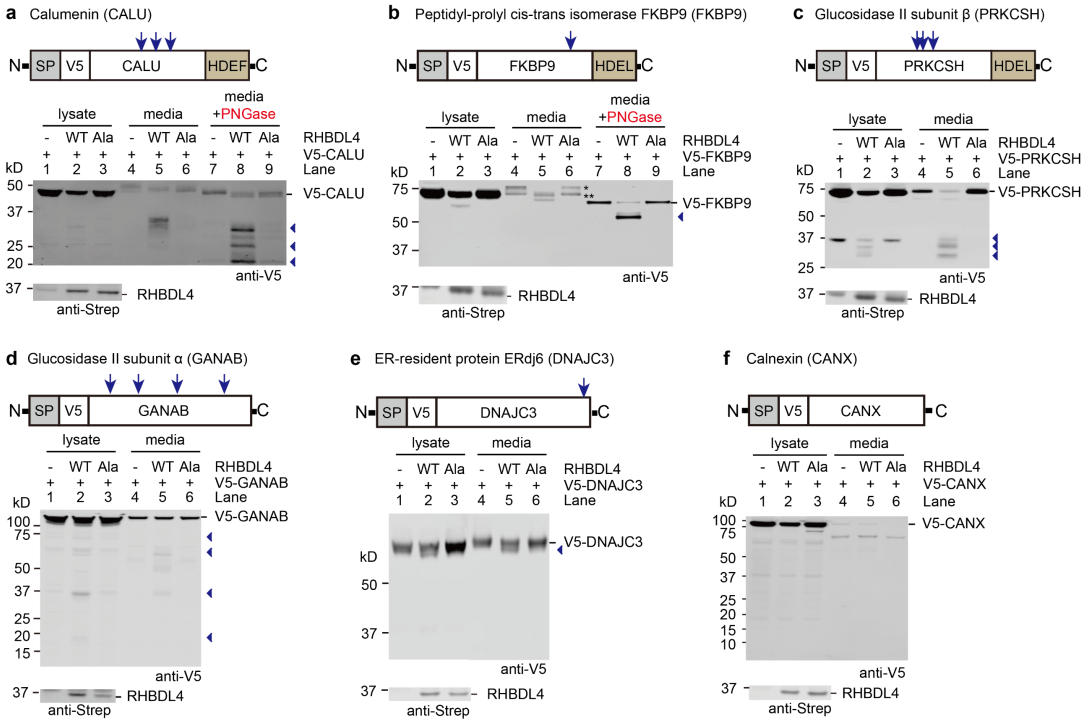

Supplementary Figure 14. RHBDL4 cleaves other ER-resident soluble chaperones. RHBDL4 cleavage assays for (a) CALU, (b) FKBP9, (c) PRKCSH, (d) GANAB, (e) DNAJC3, and (f) CANX. SP: signal peptide; V5: V5-tag. The putative cleavage sites are indicated by blue arrows. (a) WT RHBDL4 cleaved Calumenin in the internal region at multiple positions (lane 5). After PNGase treatment, multiple N-terminal fragments in media (blue triangles, lane 8) were resolved. (b) WT RHBDL4 cleaved FKBP9 at the C-terminal region. Treatment of the secreted proteins with PNGase showed that the generated N-terminal fragment (blue triangle, lane 8) was secreted into the media. *: glycosylated FKBP9; **: partially glycosylated FKBP9. (c) WT RHBDL4 cleaved Glucosidase II subunit β in the internal region at multiple positions (blue triangles, lane 2). The resulting proteolytic fragments were secreted into the media (lane 5). (d) WT RHBDL4 cleaved Glucosidase II subunit α at multiple positions. Glucosidase II subunit α, which does not have a C-terminal KDEL retention motif, is resident in the ER by interacting with Glucosidase II subunit β. The generated N-terminal fragments were also detected in the media. (e) WT RHBDL4 cleaved DNAJC3 at the C-terminus (lane 2). DNAJC3 is resident in ER by interacting with other ER resident chaperones. The generated N-terminal proteolytic fragment was secreted into the media (blue triangle, lane 5). (f) ER-resident chaperone Calnexin – which was not significantly enriched by RHBDL4(S144Dap) with respect to controls – was chosen as a negative control. No RHBDL4-dependent proteolytic bands were detected both in cell lysate and the extracellular media. (a-c) and (e) were repeated in two biological replicates, and (d) and (f) were repeated in three biological replicates, all with similar results.

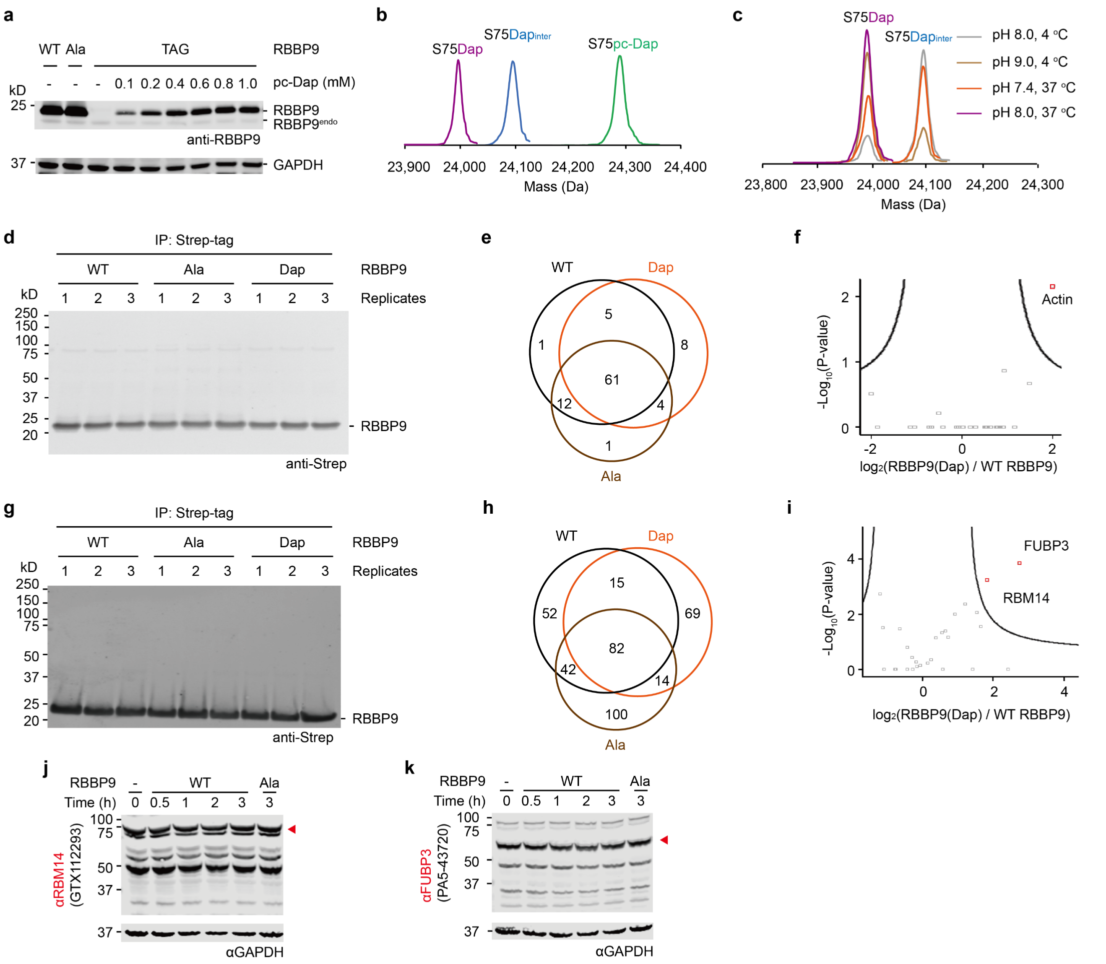

Supplementary Figure 15. RBBP9 is unlikely to be an endopeptidase in mammalian cells. (a) The expression of RBBP9(S75pc-Dap) as a function of pc-Dap concentration in the medium. The expression of RBBP9(S75pc-Dap) is not further increased when the media is supplemented with more than 0.5 mM pc-Dap. The exogenously expressed RBBP9 and endogenous RBBP9 was detected by an anti-RBBP9 antibody. GAPDH: loading control. (b) The two-step deprotection of RBBP9(S75pc-Dap) at pH 8.0, 37 °C. Green trace, purified RBBP9(S75pc-Dap)-twin-Strep: expected 24291 Da; observed 24291 Da. Blue trace, for samples directly after illumination, [RBBP9(S75$\text{Dap}_{\text{inter}}$)]-twin-Strep: expected 24098 Da; observed 24097 Da. Purple trace, proteins after illumination were incubated in Tris buffer (50 mM Tris, pH 8.0, 150 mM NaCl, 5 mM DTT) for 24 h at 37 °C, [RBBP9(S75Dap)]: expected 23994 Da; observed 23993.5 Da. (c) The second step deprotection relies on relatively high pH values. Only 40% intermediate was converted to fully deprotected RBBP9(S75Dap) at pH 7.4 after 24 h incubation. In comparison, the intermediate was entirely converted to fully deprotected product at pH 8. (d-f) are data from cell lysate experiments, and (g-i) are data from in live cell experiments. (d) WT RBBP9, RBBP9(S75A) or RBBP9(S75Dap) (1 μM) was incubated with Expi293 cell lysate for 3 h. RBBP9 variants were enriched by Strep-tag pulldown. The proteins eluted from beads were analyzed by an anti-Strep antibody. No higher MW bands were detected in the elution from RBBP9(S75Dap). (e) Comparison of the proteins identified in the RBBP9(S75Dap) elution to the WT RBBP9 and RBBP9(S75A) controls. Proteins identified by LC-MS/MS in at least two of the three replicates were considered as

positive identified in this analysis. (f) Volcano plot based on the LFQ values for the proteins identified in RBBP9(S75Dap) and WT RBBP9 samples from the cell lysate experiments. The black line represents the cut-off curve for significance ($S_0 = 1$, FDR < 0.01). Each data point is calculated in Perseus using $n = 3$ for each RBBP9 variant. (g) Immunoblotting analysis of RBBP9 variants enriched from an equal number of HEK293T cells after performing substrate trapping procedure in live cells. The proteins eluted from beads were analyzed by an anti-Strep antibody. Similarly, no higher MW bands were detected in the elution from RBBP9(S75Dap). Proteomic analysis of (h) and (i) was performed the same way as described in (e) and (f), respectively. Two proteins were enriched in RBBP9(S75Dap) elution after LFQ analysis. WB analysis for (j) RBM14 and (k) FUBP3 in Expi293 cell lysate treated with 1 μM WT RBBP9 or RBBP9(S75A) for the indicated time points. No specific cleavage was observed in either case by an anti-RBM14 or an anti-FUBP3 antibody, respectively. (a), (j) and (k) were performed in two biological replicates with similar results. (d) and (g) were performed in biological triplicate.

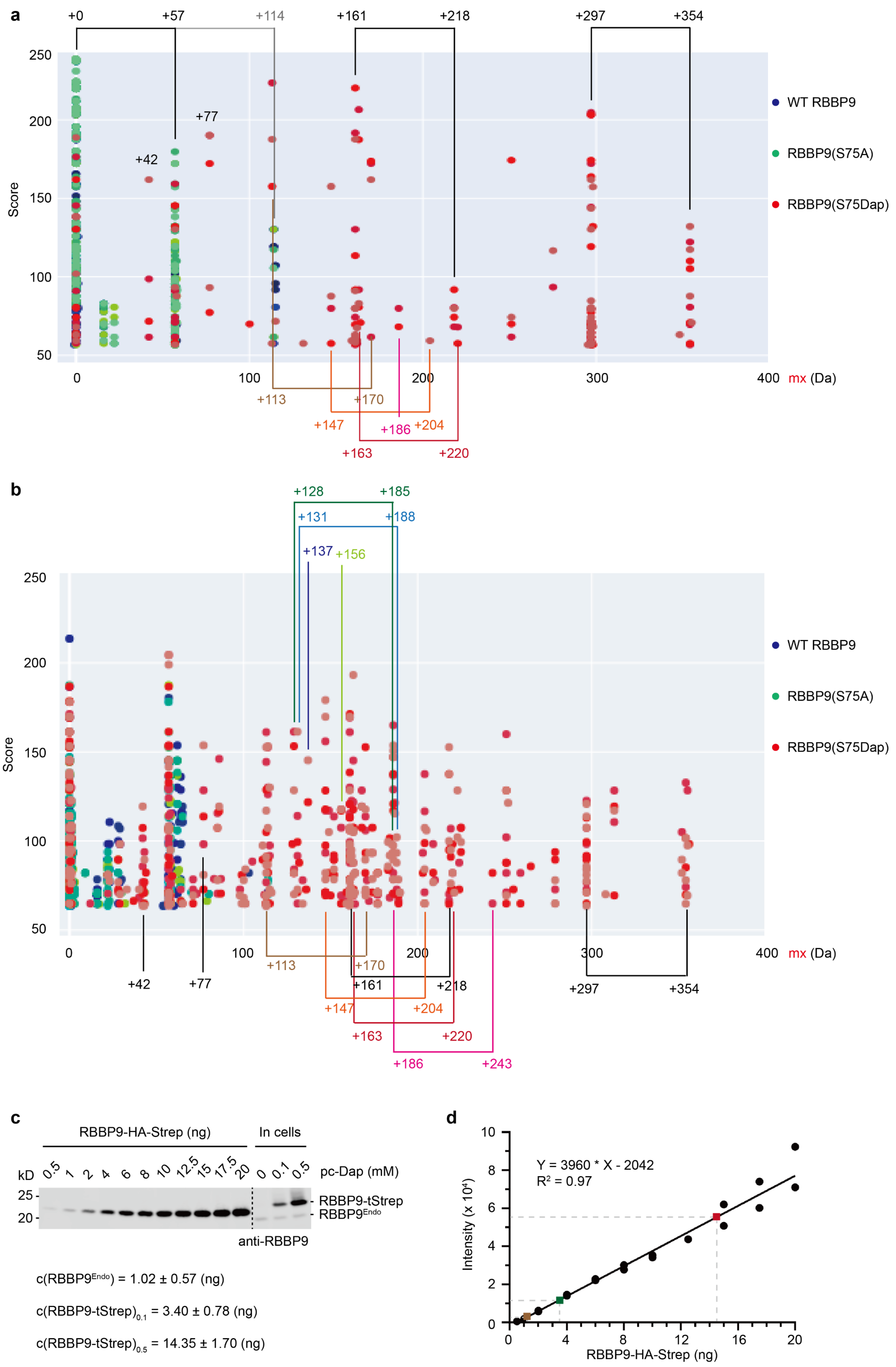

Supplementary Figure 16. The masses of X (mx) obtained from the top-scoring spectra from WT RBBP9, RBBP9(S75A), or RBBP9(S75Dap), which were treated with iodoacetamide before trypsin digestion. Each dot represents the mass shift of the MW of the peptide relative to the parental Dap- (or Ser- or Ala-) containing peptide

and the score for the peptide, calculated as described in **Methods**. (a) mx obtained from RBBP9(S75Dap) samples which were enriched from cells expressing RBBP9(S75pc-Dap) in the presence of 0.1 mM pc-Dap. Because His74 can partially react with iodoacetamide, we observed +57 pairs as illustrated in the figure for +0/+57 (parental, +114 was also observed), +113/+170 (L/I), +147/+204 (F), +163/+220 (Y) and +297/+354 (pc-Dap). In addition, the Pept(Dap$_{inter}$), in its unmodified form is a +104 Da adduct, was captured as a +161 Da adduct after reaction with iodoacetamide (and also a small population of +218 due to the His alkylation). The corresponding tryptic peptides derived from near-cognate suppression of amber codon by glutamine and tyrosine in place of Dap were observed with mass shifts of +42 Da and +77 Da, respectively. (b) mx obtained from RBBP9(S75Dap) samples which were enriched from cells expressing RBBP9(S75pc-Dap) in the presence of 0.5 mM pc-Dap. In addition to +113/+170 (L/I), +147/+204 (F), +163/+220 (Y) and +186/+243 (W) pairs, we observed +128/+185 (K), +131/+188 (M), +137 (H), +156 (R). Green dots: WT RBBP9; Black dots: RBBP9(S75A); Red dots: RBBP9(S75Dap). (c) Quantifying the levels of RBBP9(S75pc-Dap) in (a) and (b) relative to endogenous RBBP9. Immunoblotting analysis of purified RBBP9-HA-Strep (0.5 – 20 ng), endogenous RBBP9 and RBBP9(S75pc-Dap)-twin-Strep (RBBP9-tStrep) in the presence of 0, 0.1 mM or 0.5 mM pc-Dap by an anti-RBBP9 antibody. (d) The standard curve of immunoblotting band intensity (Y axis) and the amount of RBBP9 (X axis). Based on the standard curve and the intensities of RBBP9 species, we calculated the amount of endogenous RBBP9 and RBBP9(S75pc-Dap) present in the same sample. Brown square: endogenous RBBP9; green square: RBBP9(S75pc-Dap) produced in the presence of 0.1 mM pc-Dap; red square: RBBP9(S75pc-Dap) produced in the presence of 0.5 mM pc-Dap. When cells were supplied with 0.1 mM pc-Dap, the production of RBBP9(S75pc-Dap) is about 3.3-fold above endogenous RBBP9 levels and RBBP9(S75Dap) selectivity captured Leu/Ile, Tyr, Phe and Trp. However, when cells were supplied with 0.5 mM pc-Dap, the production of RBBP9(S75pc-Dap) is about 14-fold above endogenous RBBP9 levels; under these conditions RBBP9(S75Dap) still captured Ile/Leu, Tyr, Phe and Trp, but also captured Lys, Met, His and Arg. We subsequently demonstrated that these amino acids, with aliphatic and charged side chains, are poorer substrates for RBBP9 mediated cleavage than Leu/Ile, Phe, Tyr and Trp (Fig. 5a). (a) and (b) were performed in biological triplicate. (c) was performed in two biological replicates. (d) was generated using n = 2 independent measurements of the band intensities from (c) in Image Studio Lite.

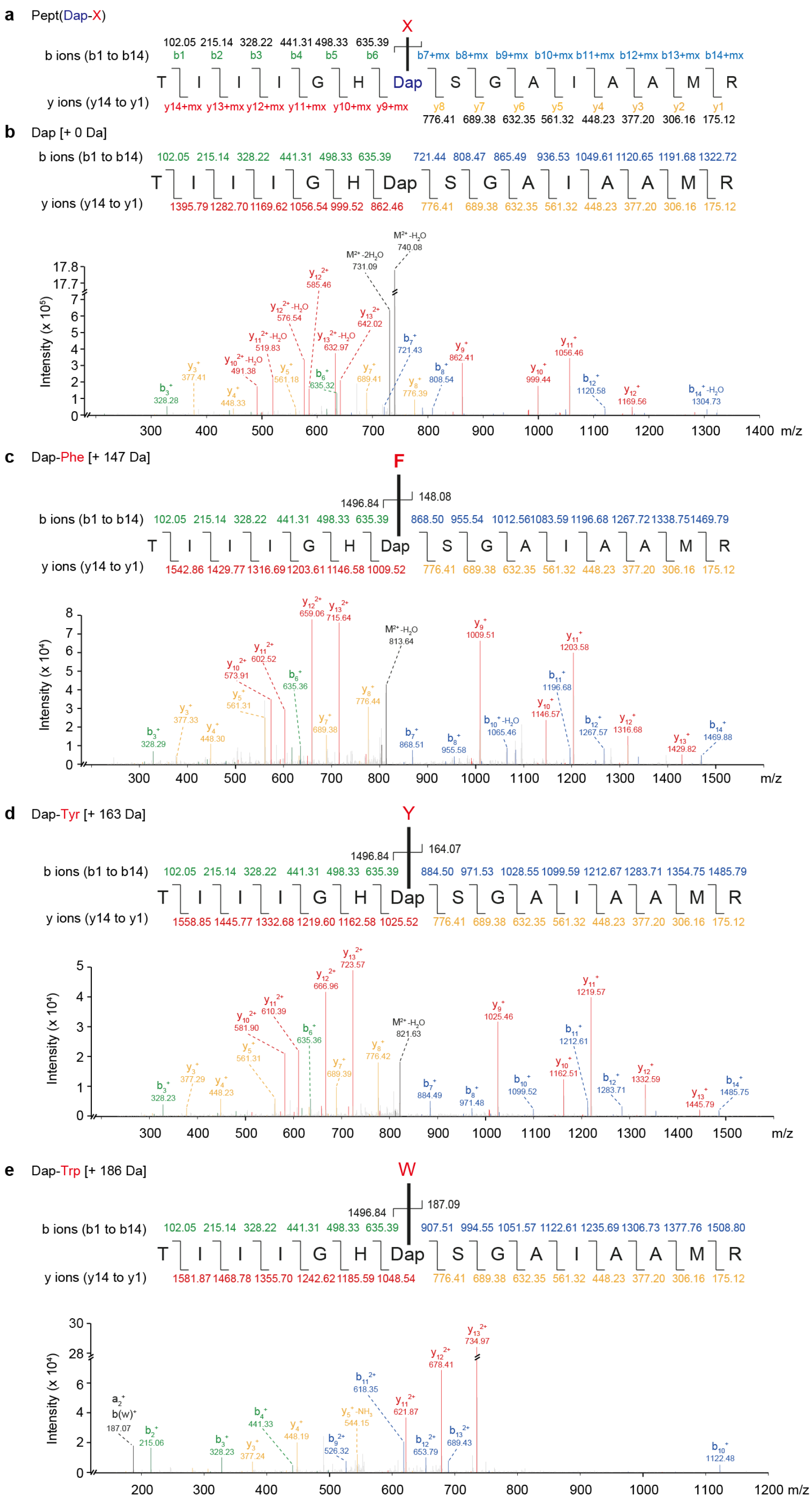

Supplementary Figure 17. LC-MS/MS characterization of Pept(Dap-X) reveals aromatic amino acids conjugation to RBBP9(S75Dap). (A) Calculation of the theoretical b and y ions based on the peptide sequence and X. LC-MS/MS spectra and peak assignment for (B) X = 0 (Pept(Dap)), (C) X = 147 (Pept(Dap-F)), (D) X = 163 (Pept(Dap-Y)), and (E) X = 186 (Pept(Dap-W)).

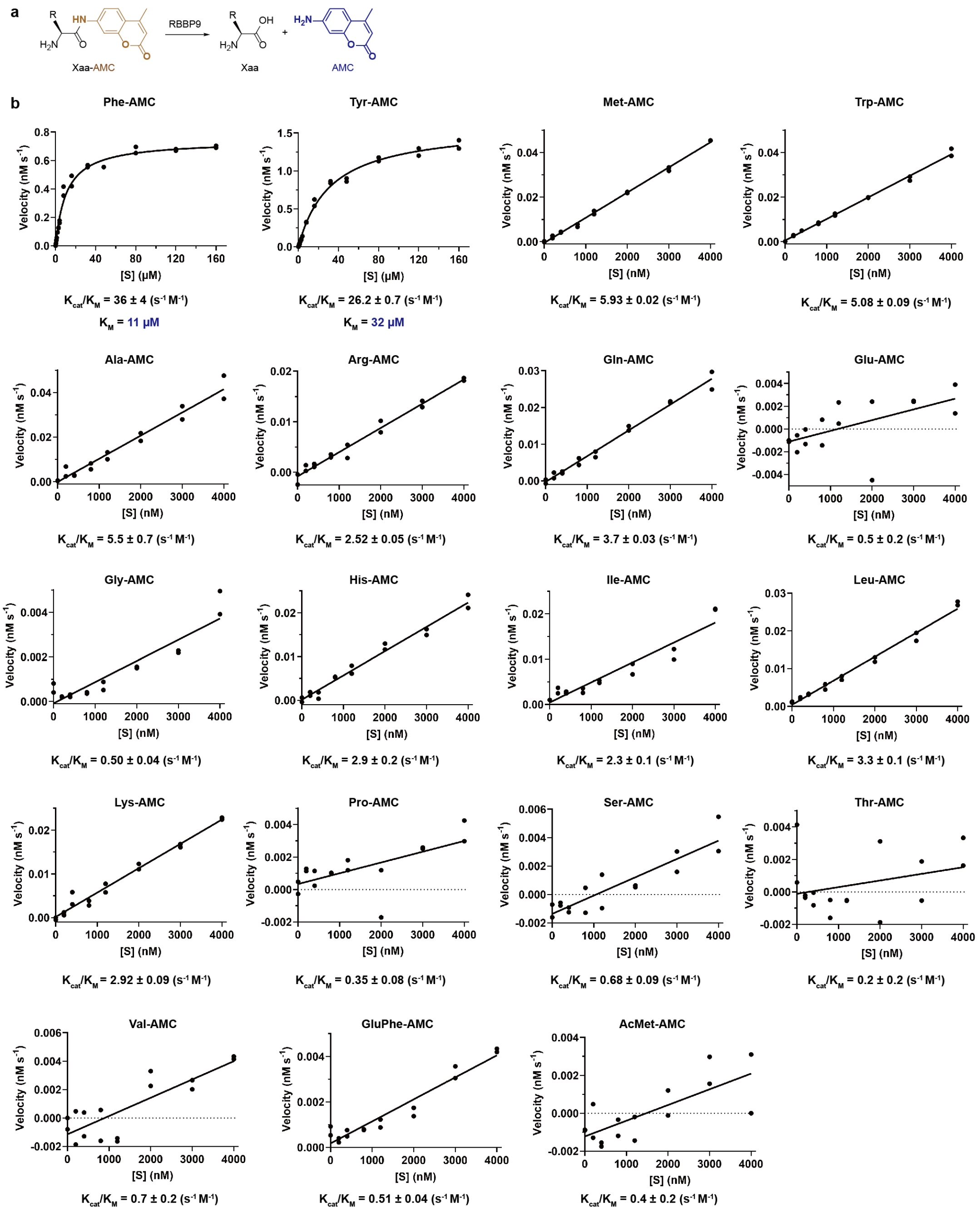

Supplementary Figure 18. Aminopeptidase activity assay of RBBP9 on 19 aa-AMC compounds. (a) The mechanism of the fluorescence-based aminopeptidase activity assay of RBBP9. RBBP9 hydrolyzes the amide bond between amino acid (aa) and 7-amino-4-methylcoumarin (AMC). The reaction kinetics was measured based on the accumulation of AMC. (b) The specificity constants obtained for 19 aa-AMC compounds. Michaelis Menten kinetics was employed for Phe-AMC and Tyr-AMC, and the pseudo-first order kinetics was employed to calculate specificity constants for the others, as described in **Methods**. The linear regression was calculated using n = 2 independent measurements.

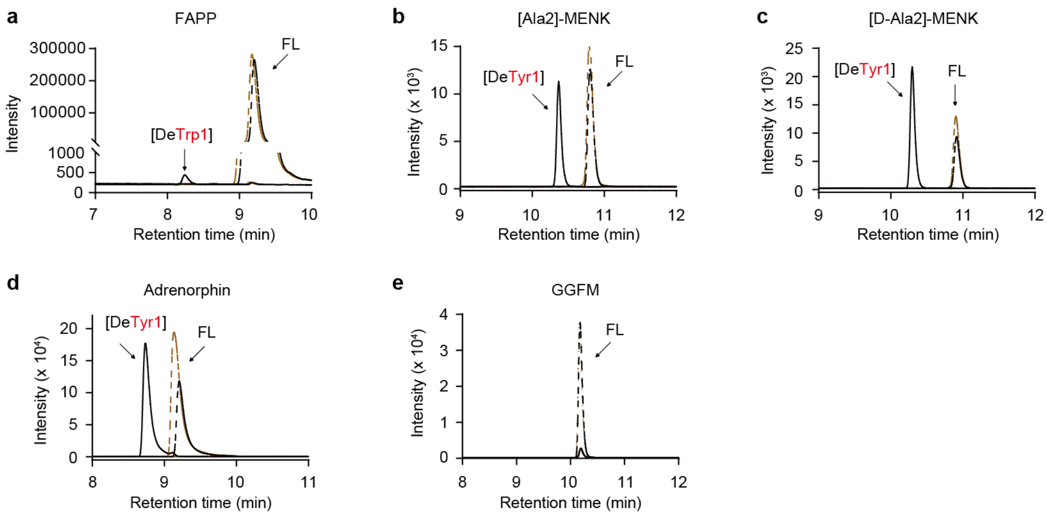

Supplementary Figure 19. RBBP9 has aminopeptidase activity on peptides. WT RBBP9 or RBBP9(S75A) (2 μM) was incubated with (a) Fibronectin adhesion-promoting peptide (FAPP) (100 μM), (b) [Ala2]-MENK (100 μM), (c) [D-Ala2]-MENK (100 μM), (d) Adrenorphin (100 μM), and (e) GGFM (100 μM) in tris buffer (pH 8) for 8 h. The original full-length (FL) peptide and the product after removing the first amino acid was determined by mass spectrometry. Black solid line: detection of product after incubating with WT RBBP9; brown solid line: detection of product after incubating with RBBP9(S75A); black dashed line: detection of FL peptide after incubating with WT RBBP9; brown dashed line: detection of FL peptide after incubating with RBBP9(S75A). The peptide sequence and the corresponding mass are summarized in Supplementary Table 5. The aminopeptidase activity of RBBP9 on MENK analogs were not affected when the second amino acid glycine is replaced with L-alanine or D-alanine.

Supplementary Table 1. Comparison to substrates identified by the COFRADIC approach (N-terminomics)[17]

Reported	Protein name	Found	Protein name	Note
P68133[a]	Actin, alpha skeletal muscle	P63261	Actin, cytoplasmic 2	Enriched
Q71U36[b]	Tubulin alpha-1A chain	P68363	Tubulin alpha-1B chain	Enriched
P07437	Tubulin beta chain	P07437	Tubulin beta chain	Enriched
Q99867[c]	Tubulin b-4q	P68371	Tubulin beta-4B chain	Enriched
Q9BUF5[d]	Tubulin beta-6 chain	P68371	Tubulin beta-4B chain	Enriched
P08670	Vimentin	P08670	Vimentin	Not enriched
O94788	Retinal dehydrogenase 2	/	/	Not found
Q99714	HADH2	Q99714	HADH2	Exclusively found
Q04637	eIF-4G1	Q04637	eIF-4G1	Exclusively found
P68104	EF-Tu	P68104	EF-Tu	Enriched
Q13263	TIF1-beta	Q13263	TIF1-beta	Enriched
P27708	CAD	P27708	CAD	Enriched
O75351[e]	VPS4B	Q9UN37	VPS4A	Exclusively found
Q8N163[f]	CCAR2	/	/	Not found
Q86XE2[g]	PDXDC1	/	/	Not found

Note: [a]: P63261 (Actin, cytoplasmic 2) instead of P68133 (Actin, alpha skeletal muscle) should be the correct protein identified according to the identified peptide sequences.

[b]: Peptides identified by N-terminomics also belong to P68363 (Tubulin alpha-1B chain), which was enriched in HtrA2(S306Dap) samples.

[c,d]: Peptides identified by N-terminomics also belong to P68371 (Tubulin beta-4B chain), which was enriched in HtrA2(S306Dap) samples. In addition, Q99867 was deleted from Uniprot on January 25, 2012.

[e]: Peptides identified by N-terminomics also belong to Q9UN37 (VPS4A), which was exclusively found HtrA2(S306Dap) samples.

[f]: Q8N163 is CCAR2.

[g]: Q86XE2 is PDXDC1.

Enriched: Proteins enriched based on LFQ analysis.

Not enriched: Proteins found in all samples, but not enriched in HtrA2(S306Dap) samples.

Exclusively found: Protein only detected in HtrA2(S306Dap) samples. These proteins are highlighted in red in Supplementary Data File 1.

Supplementary Table 2. Summary of enriched proteins in HtrA2(S306Dap) compared to WT HtrA2

Protein IDs (Uniprot)	Gene name	p-Value**	Enrichment (fold)	MW (KD)	antibody	Company	Substrates
P24928	POLR2A	6.31E-04	84.45	217	ab76123	abcam	✓
P58107	EPPK1	1.26E-04	73.52	558	/*	/	/
Q9NU22	MDN1	7.94E-03	68.59	633	A304-739A-T	Bethyl Laboratories	✓
C9J2Y9	POLR2B	5.01E-04	45.25	134	ab228933	abcam	✓
Q14204	DYNC1H1	1.00E-02	39.40	532	ab245554	abcam	✓
Q92616	GCN1	5.01E-04	34.30	326	ab86139	abcam	✓
O15027	SEC16A	1.58E-06	29.86	252	ab70722	abcam	✓
P49792	RANBP2	1.00E-04	27.86	358	ab2938	abcam	✓
P49327	FASN	3.98E-04	24.25	273	ab128870	abcam	✗
Q9NR09	BIRC6	1.26E-03	19.70	530	ab19609	abcam	✓
Q00610	CLTC	3.98E-04	17.15	192	ab172958	abcam	✓
P27708	CAD	1.58E-02	16.00	243	/	/	Reported
Q15149	PLEC	6.31E-04	12.13	532	ab32528	abcam	✓
P33993	MCM7	1.58E-03	12.13	81	GTX110278	GeneTex	✓
P19387	POLR2C	1.58E-03	12.13	31	ab182150	abcam	✓
Q9BWF3	RBM4	3.98E-05	10.56	40	ab251923	abcam	✓
Q15645	TRIP13	1.26E-03	10.56	49	ab128171	abcam	✓
Q13615	MTMR3	3.98E-03	9.85	134	H00008897-B01P	Novus Biologicals	✓
P07814	EPRS	3.16E-02	9.19	171	ab31531	abcam	✓
P31689	DNAJA1	2.51E-02	8.57	45	ab126774	abcam	✗
O75643	SNRNP200	2.00E-02	8.00	245	23875-1-AP	Proteintech	✗
Q96PK6	RBM14	2.00E-06	8.00	70	ab228692	abcam	✓
P63244	RACK1	7.94E-03	7.46	35	ab129084	abcam	✓

Q9NZB2	FAM120A	1.00E-02	6.96	122	GTX120824	GeneTex	✓
O14654	IRS4	6.31E-03	6.50	134	ab52622	abcam	✓
Q9H6R7	WDCP	1.26E-03	4.92	79	A303-337-T	Bethyl Laboratories	✓
P25705	ATP5F1A	2.51E-03	4.92	60	ab176569	abcam	✘
P04181	OAT	7.94E-04	4.29	49	ab137679	abcam	✓
P49411	TUFM	7.94E-05	4.00	50	ab173300	abcam	✓
Q13263	TIF-1b	1.26E-05	3.73	89	/	/	Reported

*: We could not source an antibody suitable for WB analysis.

**: The p-value was generated in Perseus using n = 4 measurements.

Supplementary Table 3. Summary of enriched proteins in HtrA2(S306Dap) compared to HtrA2(S306A)

Protein IDs (Uniprot)	Gene name	p-Value**	Enrichment (fold)	MW (KD)	antibody	Company	Substrates
Q9NU22	MDN1	3.98E-03	73.52	633	A304-739A-T	Bethyl Laboratories	✓
P27708	CAD	7.94E-03	32.00	243	/	/	Reported
Q92616	GCN1	1.00E-04	27.86	326	ab86139	abcam	✓
P24928	POLR2A	6.31E-04	84.45	217	ab76123	abcam	✓
C9J2Y9	POLR2B	5.01E-03	24.25	134	ab228933	abcam	✓
P49327	FASN	6.31E-04	22.63	273	ab128870	abcam	✗
Q14204	DYNC1H1	7.94E-03	21.11	532	ab245554	abcam	✓
P58107	EPPK1	2.00E-03	17.15	558	/*	/	/
P63244	RACK1	1.58E-03	13.93	35	ab129084	abcam	✓
P49411	TUFM	3.16E-06	11.31	50	ab173300	abcam	✓
P15924	DSP	3.98E-02	10.56	332	MAB9080	R&D system	✓
P23396	RPS3	1.00E-03	9.85	27	A2533	Abclonal	✓
Q13263	TIF1-b	3.16E-04	8.57	89	/	/	Reported
Q9BWF3	RBM4	3.98E-05	10.56	40	ab251923	abcam	✓
Q9NR09	BIRC6	7.94E-03	7.46	530	ab19609	abcam	✓
P68363	TUBA1B	5.01E-06	6.50	50	/	/	Reported
P07437	TUBB	1.00E-04	6.50	50	/	/	Reported
P68371	TUBB4B	6.31E-05	5.66	50	/	/	Reported
P68104	EEF1A1	2.00E-05	5.28	50	/	/	Reported
P63261	ACTG1	2.00E-05	5.28	42	/	/	Reported

*: We could not source an antibody suitable for WB analysis. **: The p-value was generated in Perseus using n =4 measurements.

Supplementary Table 4. Potential substrates identified by RHBDL4(S144Dap).

Entry	Protein ID	Gene name	Protein name	Compartment	RHBDL4(S144Dap) vs WT RHBDL4		RHBDL4(S144Dap) vs RHBDL4(S144A)	
					p-Value	Enrichment (fold)	p-Value	Enrichment (fold)
1*	Q9BS26	ERP44	ER protein 44	ER	2.344E-03	70.03	1.349E-03	23.92
2*	P11021	HSPA5	ER chaperone BiP	ER	1.096E-04	53.08	1.230E-04	12.82
3	P52272	HNRNPM	hnRNP M	Nucleus	6.761E-03	10.78	3.260E-03	1.76
4	P31943	HNRNPH1	hnRNP H	Nucleus	1.585E-04	7.94	2.754E-05	4.44
5	O00116	AGPS[a]	Alkyl-DHAP synthase	Peroxisome	3.890E-03	7.11	5.152E-02	0.58
6	P20700	LMNB1[a]	Lamin-B1	Nuclear inner membrane (lipid anchor)	1.047E-02	5.31	6.187E-01	1.13
7*	O95140	MFN2	Mitofusin-2	ER/Mitochondrion (transmembrane)	1.047E-02	5.13	2.009E-03	2.01
8*	Q14697	GANAB	Neutral alpha-glucosidase AB	ER	1.202E-02	4.59	5.861E-03	2.19
9	P06493	CDK1	Cyclin-dependent kinase 1	Nucleus, Mitochondrion	1.995E-03	4.14	4.169E-03	3.56
10	O00571	DDX3X	ATP-dependent RNA helicase DDX3X	Nucleus, Cytosol	2.239E-03	3.84	4.732E-03	1.91
11*	Q9NXE4	SMPD4[a]	Sphingomyelin phosphodiesterase 4	ER (transmembrane)	8.318E-03	3.73	4.305E-01	1.15
12*	Q9HD20	ATP13A1[a]	Manganese-transporting ATPase 13A1	ER (transmembrane)	4.898E-04	3.32	8.913E-01	0.97

13	Q8N1F7	NUP93[b]	Nucleoporin Nup93	Nuclear pore complex	N.D.	∞	3.236E-03	6.23
14	P26368	U2AF2	hU2AF65	Nucleus	5.834E-03	3.17	3.890E-03	6.02
15[*]	Q15084	PDIA6 [b]	Protein disulfide-isomerase A6	ER	N.D.	∞	5.370E-05	4.76
16	Q16778	H2BC21 [b]	Histone H2B.q	Nucleus	N.D.	∞	5.623E-04	4.44
17[**]	P50402	EMD[c]	Emerin	Nuclear inner membrane (transmembrane)	1	11.79	2.818E-02	3.94
18	Q14331	FRG1	Protein FRG1	Nucleus	4.102E-02	1.64	1.549E-03	3.14
19[*]	Q96A33	CCDC47[c]	Coiled-coil domain-containing protein 47	ER (transmembrane)	1	3.02	1.585E-03	2.77
20[***]	Q8N6H7	ARFGAP2[d]	ARF GAP 2	Golgi, Peripheral membrane	N.D.	∞	N.D.	∞
21[*]	P27797	CALR [d]	Calreticulin	ER	N.D.	∞	N.D.	∞
22[*]	O43852	CALU [d]	Calumenin	ER	N.D.	∞	N.D.	∞
23[***]	Q9H444	CHMP4B [d]	Charged multivesicular body protein 4b	Endosome, Peripheral membrane	N.D.	∞	N.D.	∞
24	Q9NZN8	CNOT2 [d]	CCR4-associated factor 2	Nucleus	N.D.	∞	N.D.	∞
25[***]	O75718	CRTAP [d]	Cartilage-associated protein	Extracellular matrix	N.D.	∞	N.D.	∞
26[*]	Q13217	DNAJC3 [d]	ERdj6	ER	N.D.	∞	N.D.	∞
27[***]	Q14677	CLINT1 [d]	Clathrin interactor 1	Peripheral membrane	N.D.	∞	N.D.	∞
28[*]	O95302	FKBP9 [d]	PPIase FKBP9	ER	N.D.	∞	N.D.	∞
29[*]	P14314	PRKCSH [d]	Glucosidase 2 subunit beta	ER	N.D.	∞	N.D.	∞
30	P78318	IGBP1 [d]	Immunoglobulin-	Cytosol	N.D.	∞	N.D.	∞

			binding protein 1					
31**	Q8NC56	LEMD2 [d]	hLEM2	Nuclear inner membrane (transmembrane)	N.D.	∞	N.D.	∞
32*	Q14494	NFE2L1 [d]	ER membrane sensor NFE2L1	ER, Nuclear inner membrane (transmembrane)	N.D.	∞	N.D.	∞
33*	O15460	P4HA2 [d]	Prolyl 4-hydroxylase subunit alpha-2	ER	N.D.	∞	N.D.	∞
34	Q96PU8	QKI [d]	Protein quaking	Nucleus	N.D.	∞	N.D.	∞
35	Q9BWF3	RBM4 [d]	RNA-binding protein 4	Nucleus, cytosol	N.D.	∞	N.D.	∞
36***	Q6WKZ4	RAB11FIP1 [d]	Rab11-FIP1	Endosome, Phagosome	N.D.	∞	N.D.	∞
37***	P07602	PSAP [d]	Prosaposin	Lysosome	N.D.	∞	N.D.	∞
38***	O15027	SEC16A [d]	Protein transport protein Sec16A	Cytosol, ER peripheral membrane	N.D.	∞	N.D.	∞
39	Q9BT92	TCHP [d]	Protein TCHP	Mitochondrion, Cytoskeleton, plasma membrane	N.D.	∞	N.D.	∞
40	P31483	TIA1 [d]	Nucleolysin TIA-1 isoform p40	Nucleus	N.D.	∞	N.D.	∞
41***	Q6ZVM7	TOM1L2 [d]	TOM1-like protein 2	Extracellular, endosome, exosome	N.D.	∞	N.D.	∞
42	P50616	TOB1 [d]	Protein Tob1	Nucleus	N.D.	∞	N.D.	∞
43	Q969T9	WBP2 [d]	WW domain-binding protein 2	Nucleus	N.D.	∞	N.D.	∞

[a]: Candidates enriched significantly in RHBDL4(S144Dap) samples compared to WT RHBDL4, but not significantly enriched compared to RHBDL4(S144A) samples.

[b]: Candidates enriched significantly in RHBDL4(S144Dap) samples compared to RHBDL4(S144A) samples, but not found in WT RHBDL4 samples.

[c]: Candidates enriched significantly in RHBDL4(S144Dap) samples compared to either WT RHBDL4 or RHBDL4(S144A) samples, but the p-Value for WT RHBDL4 samples is 1.

[d]: Candidates only found in RHBDL4(S144Dap) samples, but not detected in WT RHBDL4 or RHBDL4(S144A) samples.

*: ER resident proteins; **: Nuclear transmembrane proteins; ***: Proteins in secretory pathways.

Supplementary Table 5. RBBP9 aminopeptidase activity on peptides.

Name	Amino acid Sequence	$[M+H]^+$	$[[M\text{-}Xaa_1]+H]^+$	$[M+2H]^{2+}$	$[[M\text{-}Xaa_1]+2H]^{2+}$	Substrate
Nociceptin	FGGFTGARKSARKLANQ	1810.1	1662.9	905.5*	832*	✔
MENK	YGGFM	574.7*	411.5*	-	-	✔
[Ala_2]-MENK	YAGFM	588.7*	425.5*	-	-	✔
[D-Ala_2]-MENK	Y[d-Ala]GFM	588.7*	425.5*	-	-	✔
Adrenorphin	YGGFMRRV	986.2	823	493.6*	412*	✔
FAPP (Fibronectin adhesion-promoting peptide)	WQPPRARI	1024.2	838	512.6*	419.5*	✔
GGFM ([$DeTyr_1$]-MENK)	GGFM	411.5*	354.4*	-	-	✘

*: Masses used for SIM detection.

Supplementary Methods

Material

Brefeldin A (00-4506-51, 1000x) was purchased from Thermo Fisher. L-Phe-AMC (sc-221810), L-Tyr-AMC (sc-207814), L-Met-AMC (sc-284923), L-Trp-AMC (sc-286111), L-Ala-AMC (sc-295253), L-Arg-AMC (sc-215211), L-Gln-AMC (sc-286085), L-Glu-AMC (sc-281541), L-Gly-AMC (sc-474569), L-His-AMC (sc-281543), L-Ile-AMC (sc-295292c), L-Leu-AMC (sc-300884), L-Lys-AMC (sc-300887B), L-Pro-AMC (sc-207808), L-Ser-AMC (sc-207811), L-Thr-AMC (sc-207812), L-Val-AMC (sc-300896), L-GluPhe-AMC (sc-215075) and AcMet-AMC (sc-284923) were purchased from Santa Cruz Biotechnology. MG132 (M8699), L-Phe (78019, BioUltra), Nociceptin (487960), MENK (M6638), [D-Ala2]-MENK (E2006), Adrenorphin (T8156) and Fibronection adhesion-promoting peptide (F3667) were purchased from Merck. Peptide [Ala2]-MENK and [DeTyr1]-MENK were purchased from Bankpeptide. Ub-AMC (U-550), SUMO1-AMC (UL-551), NEDD8-AMC (UL-552), ISG15-AMC (UL-553) were purchased from R&D Systems.

Antibodies

Secondary antibodies include IRDye 680RD secondary antibodies (Goat anti-Mouse (925-68070) and Goat anti-Rabbit (925-68071), Li-Cor) and IRDye 800CW secondary antibodies (Goat anti-Mouse (925-32210) and Goat anti-Rabbit (926-32211), Li-Cor). Primary antibodies include: anti-Strep (ab76949 (produced in rabbit), abcam; MCA2489 (produced in mouse), Bio-Rad), anti-GFP (2956(D5.1), Cell Signaling), anti-HA (3724(C29F4, produced in rabbit) and 2367(6E2, produced in mouse), Cell Signaling), anti-GAPDH (2118(14C10, produced in rabbit), Cell Signaling; TA802519 (produced in mouse), OriGene), anti-V5 (R960-25, Thermo Fisher), anti-FLAG (F3165 (produced in mouse), Merck; AHP1074 (produced in rabbit), Bio-Rad), anti-RHBDL4

(20869-1-AP, Proteintech), anti-CCDC47 (HPA029674, Merck), anti-BiP (GTX113340, GeneTex), anti-CALR (ab92516, abcam), anti-PDIA6 (ab154820, abcam), anti-Calnexin (10427-2-AP, Proteintech), anti-KDEL (ab176333, abcam), anti-TUFM (ab173300, abcam), anti-LaminB1 (12987-1-AP, Proteintech), anti-β-Tubulin (86298(D3U1W), Cell Signaling), anti-Ub (MAB8595, R&D systems), anti-Transferrin (66171-1-Ig, Proteintech), and anti-RBBP9 (ab157202, abcam). Antibodies used for HtrA2 substrate validation were listed in Supplementary Tables 2 and 3.

Purification of recombinant proteases in *E. coli*

C-terminal HA-Strep-tagged TEV, HtrA2(134-458), SCoV2-PLpro and UL36^{USP} were purified by StrepTrap HP column (GE Healthcare). BL21 (DE3) cells were transformed with WT, catalytically inactive mutant (catalytic Cys of TEV or SCoV2-PLpro was mutated to Ala, catalytic Cys of UL36^{USP} was mutated to Ser, and catalytic Ser of HtrA2(134-458) was mutated to Ala), or amber mutant (catalytic Cys or Ser mutated to TAG stop codon) with pMB-DapRST[15]. Transformants were grown in 2xTY media containing 10 μg/mL tetracycline (and 75 μg/mL spectinomycin for co-transformed cells) overnight at 37 °C. The overnight culture was diluted 1:100 into fresh 2xTY media supplemented with corresponding antibiotics and shaken at 37 °C. 100 μM pc-Dap was added to co-transformed cells when OD_{600} reached 0.3. When OD_{600} reached around 0.6, cultures were moved to 20 °C, and protein expression was induced by adding 250 μM isopropyl β-D-1-thiogalactopyranoside (IPTG) for 20 h. Cells were harvested by centrifugation and resuspended in lysis buffer (50 mM Tris-HCl pH 7.5, 150 mM NaCl, 2 mM DTT, 0.5 mg/mL lysozyme (Sigma), 50 μg/mL DNase (Sigma)) and lysed by sonication. When purifying catalytically inactive or amber mutant, protease inhibitors (Roche) were added in the lysis buffer. The lysate was centrifuged at 40,000 g for 30 min and filtered through a 0.45 μm polyethersulfone membrane. The

cleared lysate was loaded to 1 mL or 5 mL StrepTrap HP column. After sample loading, the column was washed with Tris buffer (50 mM Tris pH 8.0, 150 mM NaCl, 1 mM EDTA, 5 mM DTT). Proteins were eluted using a linear gradient of desthiobiotin (0 to 2.5 mM). The protein containing fractions were analyzed by SDS-PAGE and collected accordingly. Purified proteins were frozen in 10% glycerol and stored at -80 °C.

Cleavage of UBL-AMC

Recombinant proteases (WT UL36^{USP}, UL36^{USP}(C65S), SCoV2-PLpro or SCoV2-PLpro(C111A), 2 nM) were diluted in 50 µL HEPES buffer (50 mM HEPES, pH 7.5, 150 mM NaCl, 0.5 mM EDTA and 1 mM DTT). 50 µL of UBL-AMC (Ub-AMC, SUMO1-AMC, NEDD8-AMC or ISG15-AMC, 200 nM) in HEPES buffer was mixed with the protease solution. Release of AMC fluorescence was monitored with an excitation wavelength of 345 nm and an emission wavelength of 445 nm at 25 °C.

Protease(Dap) conjugation with UBL-AMC

UL36^{USP}(C65Dap) or SCoV2-PLpro(C111Dap) (1 µM) was incubated with UBL-AMC (5 µM) in Tris buffer (50 mM Tris, pH 8.0, 150 mM NaCl, 0.5 mM EDTA, 5 mM DTT) at 37 °C. 10 µL of each reaction taken at indicated time points was stopped by mixing with 3.5 µL of 4 x LDS loading buffer and heated at 65 °C for 15 min. The conjugate formation was analyzed by an anti-HA antibody against the C-terminal HA-tag of proteases.

Flow Cytometry analysis

48 h after transfection, cells in 24-well plates were washed with PBS, detached by trypsin/EDTA solution, and resuspended in the growth medium. Cells were pelleted and resuspended in PBS supplemented with 3% FBS. Cells were analyzed using Becton Dickinson LSRFortessa (407 nm violet laser (V-450) for BFP excitation, 488 nm blue

laser (B-525) for GFP excitation, 561 nm yellow-green laser (YG-610) for mCherry excitation). The front scatter (FSC) and side scatter (SSC) were used to identify intact cells. Non-transfected cells, cells only transfected with mCherry or eGFP plasmid, and BFP positive cells were used to define the gate. Mean background fluorescence from non-transfected cells was subtracted from the measured signal. The data was analyzed in FCS Express 7 software (De Novo software).

Immunofluorescence (IF)

40 h after co-transfection of DapRST with WT RHBDL4-GFP or RHBDL4(S144TAG)-GFP plasmid (0.5 mM pc-Dap was added to the media for incorporation), HEK293T cells were fixed by 4% paraformaldehyde (PFA) and permeabilized by incubating in PBS containing 0.2% Triton X-100 at RT for 30 min. Then, cells were blocked with blocking buffer (5% BSA in PBST) and incubated in diluted primary antibodies (anti-Calnexin (1:250), anti-LaminB1 (1:250), or anti-TUFM (1:250)) in blocking buffer at 4 °C overnight. Alexa Fluor 546-conjugated secondary antibody (1:500, A10040, Thermo Fisher) was added at RT for 1 h. The slides were finally stained with DAPI (D9542, Merck), mounted and analyzed under Zeiss LSM 710 Upright microscope. The images were analyzed by Fiji (ImageJ2).

LC-MS/MS characterization of the branched tryptic peptide of TEV(Dap)-GFP

The LC-MS/MS file (in RAW format) was first converted to mzML format using ProteoWizard. Data preparation and processing were then performed using custom Python scripts written with the pyOpenMS package. In brief, collected spectra were centroided and all MS2 spectra with a precursor mass lower than that of the unconjugated Dap-containing tryptic peptide from TEV were filtered out. For each filtered MS2 spectrum, the ten most abundant peaks in each 100 Th mass interval were extracted.

Based on the tryptic peptide sequence of the TEV-derived chain and the GFP-derived chain, a list of theoretical ion masses was calculated; these corresponded to the MS2 fragmentation of the branched active site tryptic peptide from the TEV(Dap)-GFP conjugate. This list contained (from both peptide chains): the monocationic b- and y-ions, the dicatonic b- and y- ions (if the charge of the precursor ion was at least two), and ions corresponding to water or ammonium losses from the side-chains of b- or y-ions. Peaks in the MS2 spectrum were matched against this list, and a score for this matching was calculated as previously described. The top-scoring spectrum was extracted; the molecular weight of the precursor ion and the peaks in the spectrum were manually interrogated to verify the assignment.

Supplementary Data File 1: 237 proteins uniquely identified in HtrA2(S306Dap) samples

Accession	Protein Name
ZSWM8_HUMAN	Zinc finger SWIM domain-containing protein 8 OS=Homo sapiens GN=ZSWIM8 PE=1 SV=1
ZNF24_HUMAN	Zinc finger protein 24 OS=Homo sapiens GN=ZNF24 PE=1 SV=4
ZFYV1_HUMAN	Zinc finger FYVE domain-containing protein 1 OS=Homo sapiens GN=ZFYVE1 PE=1 SV=1
ZC3HE_HUMAN	Zinc finger CCCH domain-containing protein 14 OS=Homo sapiens GN=ZC3H14 PE=1 SV=1
XRCC5_HUMAN	X-ray repair cross-complementing protein 5 OS=Homo sapiens GN=XRCC5 PE=1 SV=3
XPO2_HUMAN	Exportin-2 OS=Homo sapiens GN=CSE1L PE=1 SV=3
XP32_HUMAN	Skin-specific protein 32 OS=Homo sapiens GN=XP32 PE=1 SV=1
VPS4A_HUMAN	Vacuolar protein sorting-associated protein 4A OS=Homo sapiens GN=VPS4A PE=1 SV=1
UTRO_HUMAN	Utrophin OS=Homo sapiens GN=UTRN PE=1 SV=2
UGGG1_HUMAN	UDP-glucose:glycoprotein glucosyltransferase 1 OS=Homo sapiens GN=UGGT1 PE=1 SV=3
UFL1_HUMAN	E3 UFM1-protein ligase 1 OS=Homo sapiens GN=UFL1 PE=1 SV=2
UCK2_HUMAN	Uridine-cytidine kinase 2 OS=Homo sapiens GN=UCK2 PE=1 SV=1
UBR5_HUMAN	E3 ubiquitin-protein ligase UBR5 OS=Homo sapiens GN=UBR5 PE=1 SV=2
UBR4_HUMAN	E3 ubiquitin-protein ligase UBR4 OS=Homo sapiens GN=UBR4 PE=1 SV=1
UBP2L_HUMAN	Ubiquitin-associated protein 2-like OS=Homo sapiens GN=UBAP2L PE=1 SV=2
U2AF2_HUMAN	Splicing factor U2AF 65 kDa subunit OS=Homo sapiens GN=U2AF2 PE=1 SV=4
TSC2_HUMAN	Tuberin OS=Homo sapiens GN=TSC2 PE=1 SV=2
TRAF2_HUMAN	TNF receptor-associated factor 2 OS=Homo sapiens GN=TRAF2 PE=1 SV=2
TRAD1_HUMAN	TRAF-type zinc finger domain-containing protein 1 OS=Homo sapiens GN=TRAFD1 PE=1 SV=1
TNR6B_HUMAN	Trinucleotide repeat-containing gene 6B protein OS=Homo sapiens GN=TNRC6B PE=1 SV=4
TMM33_HUMAN	Transmembrane protein 33 OS=Homo sapiens GN=TMEM33 PE=1 SV=2
TLE3_HUMAN	Transducin-like enhancer protein 3 OS=Homo sapiens GN=TLE3 PE=1 SV=2
TF3C5_HUMAN	General transcription factor 3C polypeptide 5 OS=Homo sapiens GN=GTF3C5 PE=1 SV=2
TES_HUMAN	Testin OS=Homo sapiens GN=TES PE=1 SV=1
TELO2_HUMAN	Telomere length regulation protein TEL2 homolog OS=Homo sapiens GN=TELO2 PE=1 SV=2

TCPE_HUMAN	T-complex protein 1 subunit epsilon OS=Homo sapiens GN=CCT5 PE=1 SV=1
TBL3_HUMAN	Transducin beta-like protein 3 OS=Homo sapiens GN=TBL3 PE=1 SV=2
TBB4A_HUMAN	Tubulin beta-4A chain OS=Homo sapiens GN=TUBB4A PE=1 SV=2
SYVC_HUMAN	Valine--tRNA ligase OS=Homo sapiens GN=VARS PE=1 SV=4
SYTM_HUMAN	Threonine--tRNA ligase, mitochondrial OS=Homo sapiens GN=TARS2 PE=1 SV=1
SYRC_HUMAN	Arginine--tRNA ligase, cytoplasmic OS=Homo sapiens GN=RARS PE=1 SV=2
SYQ_HUMAN	Glutamine--tRNA ligase OS=Homo sapiens GN=QARS PE=1 SV=1
SYLC_HUMAN	Leucine--tRNA ligase, cytoplasmic OS=Homo sapiens GN=LARS PE=1 SV=2
SYIC_HUMAN	Isoleucine--tRNA ligase, cytoplasmic OS=Homo sapiens GN=IARS PE=1 SV=2
SYFA_HUMAN	Phenylalanine--tRNA ligase alpha subunit OS=Homo sapiens GN=FARSA PE=1 SV=3
SYDC_HUMAN	Aspartate--tRNA ligase, cytoplasmic OS=Homo sapiens GN=DARS PE=1 SV=2
SYAM_HUMAN	Alanine--tRNA ligase, mitochondrial OS=Homo sapiens GN=AARS2 PE=1 SV=1
STRN4_HUMAN	Striatin-4 OS=Homo sapiens GN=STRN4 PE=1 SV=2
STAU1_HUMAN	Double-stranded RNA-binding protein Staufen homolog 1 OS=Homo sapiens GN=STAU1 PE=1 SV=2
SSRP1_HUMAN	FACT complex subunit SSRP1 OS=Homo sapiens GN=SSRP1 PE=1 SV=1
SRS10_HUMAN	Serine/arginine-rich splicing factor 10 OS=Homo sapiens GN=SRSF10 PE=1 SV=1
SRPRB_HUMAN	Signal recognition particle receptor subunit beta OS=Homo sapiens GN=SRPRB PE=1 SV=3
SR140_HUMAN	U2 snRNP-associated SURP motif-containing protein OS=Homo sapiens GN=U2SURP PE=1 SV=2
SQSTM_HUMAN	Sequestosome-1 OS=Homo sapiens GN=SQSTM1 PE=1 SV=1
SPTB2_HUMAN	Spectrin beta chain, non-erythrocytic 1 OS=Homo sapiens GN=SPTBN1 PE=1 SV=2
SON_HUMAN	Protein SON OS=Homo sapiens GN=SON PE=1 SV=4
SMRC2_HUMAN	SWI/SNF complex subunit SMARCC2 OS=Homo sapiens GN=SMARCC2 PE=1 SV=1
SMCA4_HUMAN	Transcription activator BRG1 OS=Homo sapiens GN=SMARCA4 PE=1 SV=2
SMC4_HUMAN	Structural maintenance of chromosomes protein 4 OS=Homo sapiens GN=SMC4 PE=1 SV=2
SMC3_HUMAN	Structural maintenance of chromosomes protein 3 OS=Homo sapiens GN=SMC3 PE=1 SV=2
SMC1A_HUMAN	Structural maintenance of chromosomes protein 1A OS=Homo sapiens GN=SMC1A PE=1 SV=2
SLN11_HUMAN	Schlafen family member 11 OS=Homo sapiens GN=SLFN11 PE=1 SV=2
SF3B6_HUMAN	Splicing factor 3B subunit 6 OS=Homo sapiens GN=SF3B6 PE=1 SV=1

SEPT2_HUMAN	Septin-2 OS=Homo sapiens GN=SEPT2 PE=1 SV=1
SEC13_HUMAN	Protein SEC13 homolog OS=Homo sapiens GN=SEC13 PE=1 SV=3
SCAM3_HUMAN	Secretory carrier-associated membrane protein 3 OS=Homo sapiens GN=SCAMP3 PE=1 SV=3
SC11A_HUMAN	Signal peptidase complex catalytic subunit SEC11A OS=Homo sapiens GN=SEC11A PE=1 SV=1
SAMH1_HUMAN	Deoxynucleoside triphosphate triphosphohydrolase SAMHD1 OS=Homo sapiens GN=SAMHD1 PE=1 SV=2
RTN4_HUMAN	Reticulon-4 OS=Homo sapiens GN=RTN4 PE=1 SV=2
RT34_HUMAN	28S ribosomal protein S34, mitochondrial OS=Homo sapiens GN=MRPS34 PE=1 SV=2
RT29_HUMAN	28S ribosomal protein S29, mitochondrial OS=Homo sapiens GN=DAP3 PE=1 SV=1
RT22_HUMAN	28S ribosomal protein S22, mitochondrial OS=Homo sapiens GN=MRPS22 PE=1 SV=1
RT02_HUMAN	28S ribosomal protein S2, mitochondrial OS=Homo sapiens GN=MRPS2 PE=1 SV=1
RS23_HUMAN	40S ribosomal protein S23 OS=Homo sapiens GN=RPS23 PE=1 SV=3
RRP44_HUMAN	Exosome complex exonuclease RRP44 OS=Homo sapiens GN=DIS3 PE=1 SV=2
RRP12_HUMAN	RRP12-like protein OS=Homo sapiens GN=RRP12 PE=1 SV=2
RPB7_HUMAN	DNA-directed RNA polymerase II subunit RPB7 OS=Homo sapiens GN=POLR2G PE=1 SV=1
RPAP2_HUMAN	Putative RNA polymerase II subunit B1 CTD phosphatase RPAP2 OS=Homo sapiens GN=RPAP2 PE=1 SV=1
RPAP1_HUMAN	RNA polymerase II-associated protein 1 OS=Homo sapiens GN=RPAP1 PE=1 SV=3
RPAC1_HUMAN	DNA-directed RNA polymerases I and III subunit RPAC1 OS=Homo sapiens GN=POLR1C PE=1 SV=1
RPAB5_HUMAN	DNA-directed RNA polymerases I, II, and III subunit RPABC5 OS=Homo sapiens GN=POLR2L PE=1 SV=1
RPAB1_HUMAN	DNA-directed RNA polymerases I, II, and III subunit RPABC1 OS=Homo sapiens GN=POLR2E PE=1 SV=4
RPA2_HUMAN	DNA-directed RNA polymerase I subunit RPA2 OS=Homo sapiens GN=POLR1B PE=1 SV=2
RPA1_HUMAN	DNA-directed RNA polymerase I subunit RPA1 OS=Homo sapiens GN=POLR1A PE=1 SV=2
ROA1_HUMAN	Heterogeneous nuclear ribonucleoprotein A1 OS=Homo sapiens GN=HNRNPA1 PE=1 SV=5
RN114_HUMAN	E3 ubiquitin-protein ligase RNF114 OS=Homo sapiens GN=RNF114 PE=1 SV=1
RM55_HUMAN	39S ribosomal protein L55, mitochondrial OS=Homo sapiens GN=MRPL55 PE=1 SV=1
RM49_HUMAN	39S ribosomal protein L49, mitochondrial OS=Homo sapiens GN=MRPL49 PE=1 SV=1
RM48_HUMAN	39S ribosomal protein L48, mitochondrial OS=Homo sapiens GN=MRPL48 PE=1 SV=2
RM37_HUMAN	39S ribosomal protein L37, mitochondrial OS=Homo sapiens GN=MRPL37 PE=1 SV=2
RM22_HUMAN	39S ribosomal protein L22, mitochondrial OS=Homo sapiens GN=MRPL22 PE=1 SV=1

RM14_HUMAN	39S ribosomal protein L14, mitochondrial OS=Homo sapiens GN=MRPL14 PE=1 SV=1
RM09_HUMAN	39S ribosomal protein L9, mitochondrial OS=Homo sapiens GN=MRPL9 PE=1 SV=2
RM03_HUMAN	39S ribosomal protein L3, mitochondrial OS=Homo sapiens GN=MRPL3 PE=1 SV=1
RL6_HUMAN	60S ribosomal protein L6 OS=Homo sapiens GN=RPL6 PE=1 SV=3
RL35A_HUMAN	60S ribosomal protein L35a OS=Homo sapiens GN=RPL35A PE=1 SV=2
RL28_HUMAN	60S ribosomal protein L28 OS=Homo sapiens GN=RPL28 PE=1 SV=3
RL18A_HUMAN	60S ribosomal protein L18a OS=Homo sapiens GN=RPL18A PE=1 SV=2
RL15_HUMAN	60S ribosomal protein L15 OS=Homo sapiens GN=RPL15 PE=1 SV=2
RL10_HUMAN	60S ribosomal protein L10 OS=Homo sapiens GN=RPL10 PE=1 SV=4
RHEB_HUMAN	GTP-binding protein Rheb OS=Homo sapiens GN=RHEB PE=1 SV=1
RFC5_HUMAN	Replication factor C subunit 5 OS=Homo sapiens GN=RFC5 PE=1 SV=1
RFC4_HUMAN	Replication factor C subunit 4 OS=Homo sapiens GN=RFC4 PE=1 SV=2
RFC3_HUMAN	Replication factor C subunit 3 OS=Homo sapiens GN=RFC3 PE=1 SV=2
RFC2_HUMAN	Replication factor C subunit 2 OS=Homo sapiens GN=RFC2 PE=1 SV=3
RBM24_HUMAN	RNA-binding protein 24 OS=Homo sapiens GN=RBM24 PE=1 SV=1
RBM22_HUMAN	Pre-mRNA-splicing factor RBM22 OS=Homo sapiens GN=RBM22 PE=1 SV=1
RBBP7_HUMAN	Histone-binding protein RBBP7 OS=Homo sapiens GN=RBBP7 PE=1 SV=1
RAGP1_HUMAN	Ran GTPase-activating protein 1 OS=Homo sapiens GN=RANGAP1 PE=1 SV=1
RAD50_HUMAN	DNA repair protein RAD50 OS=Homo sapiens GN=RAD50 PE=1 SV=1
RAB7A_HUMAN	Ras-related protein Rab-7a OS=Homo sapiens GN=RAB7A PE=1 SV=1
RAB14_HUMAN	Ras-related protein Rab-14 OS=Homo sapiens GN=RAB14 PE=1 SV=4
RAB10_HUMAN	Ras-related protein Rab-10 OS=Homo sapiens GN=RAB10 PE=1 SV=1
PURA_HUMAN	Transcriptional activator protein Pur-alpha OS=Homo sapiens GN=PURA PE=1 SV=2
PTN23_HUMAN	Tyrosine-protein phosphatase non-receptor type 23 OS=Homo sapiens GN=PTPN23 PE=1 SV=1
PSMD8_HUMAN	26S proteasome non-ATPase regulatory subunit 8 OS=Homo sapiens GN=PSMD8 PE=1 SV=2
PSMD3_HUMAN	26S proteasome non-ATPase regulatory subunit 3 OS=Homo sapiens GN=PSMD3 PE=1 SV=2
PSMD2_HUMAN	26S proteasome non-ATPase regulatory subunit 2 OS=Homo sapiens GN=PSMD2 PE=1 SV=3
PRS8_HUMAN	26S protease regulatory subunit 8 OS=Homo sapiens GN=PSMC5 PE=1 SV=1

PRS7_HUMAN	26S protease regulatory subunit 7 OS=Homo sapiens GN=PSMC2 PE=1 SV=3
PRS4_HUMAN	26S protease regulatory subunit 4 OS=Homo sapiens GN=PSMC1 PE=1 SV=1
PRKDC_HUMAN	DNA-dependent protein kinase catalytic subunit OS=Homo sapiens GN=PRKDC PE=1 SV=3
PPT1_HUMAN	Palmitoyl-protein thioesterase 1 OS=Homo sapiens GN=PPT1 PE=1 SV=1
PPP6_HUMAN	Serine/threonine-protein phosphatase 6 catalytic subunit OS=Homo sapiens GN=PPP6C PE=1 SV=1
PP2AA_HUMAN	Serine/threonine-protein phosphatase 2A catalytic subunit alpha isoform OS=Homo sapiens GN=PPP2CA PE=1 SV=1
PO210_HUMAN	Nuclear pore membrane glycoprotein 210 OS=Homo sapiens GN=NUP210 PE=1 SV=3
PML_HUMAN	Protein PML OS=Homo sapiens GN=PML PE=1 SV=3
PLOD2_HUMAN	Procollagen-lysine,2-oxoglutarate 5-dioxygenase 2 OS=Homo sapiens GN=PLOD2 PE=1 SV=2
PLOD1_HUMAN	Procollagen-lysine,2-oxoglutarate 5-dioxygenase 1 OS=Homo sapiens GN=PLOD1 PE=1 SV=2
PLEC_HUMAN	Plectin OS=Homo sapiens GN=PLEC PE=1 SV=3
PFKAP_HUMAN	ATP-dependent 6-phosphofructokinase, platelet type OS=Homo sapiens GN=PFKP PE=1 SV=2
PELP1_HUMAN	Proline-, glutamic acid- and leucine-rich protein 1 OS=Homo sapiens GN=PELP1 PE=1 SV=2
P66A_HUMAN	Transcriptional repressor p66-alpha OS=Homo sapiens GN=GATAD2A PE=1 SV=1
P5CS_HUMAN	Delta-1-pyrroline-5-carboxylate synthase OS=Homo sapiens GN=ALDH18A1 PE=1 SV=2
P4HA1_HUMAN	Prolyl 4-hydroxylase subunit alpha-1 OS=Homo sapiens GN=P4HA1 PE=1 SV=2
OTUD4_HUMAN	OTU domain-containing protein 4 OS=Homo sapiens GN=OTUD4 PE=1 SV=4
OST48_HUMAN	Dolichyl-diphosphooligosaccharide--protein glycosyltransferase 48 kDa subunit OS=Homo sapiens GN=DDOST PE=1 SV=4
ODPB_HUMAN	Pyruvate dehydrogenase E1 component subunit beta, mitochondrial OS=Homo sapiens GN=PDHB PE=1 SV=3
ODPA_HUMAN	Pyruvate dehydrogenase E1 component subunit alpha, somatic form, mitochondrial OS=Homo sapiens GN=PDHA1 PE=1 SV=3
NU205_HUMAN	Nuclear pore complex protein Nup205 OS=Homo sapiens GN=NUP205 PE=1 SV=3
NU107_HUMAN	Nuclear pore complex protein Nup107 OS=Homo sapiens GN=NUP107 PE=1 SV=1
NSF_HUMAN	Vesicle-fusing ATPase OS=Homo sapiens GN=NSF PE=1 SV=3
NOB1_HUMAN	RNA-binding protein NOB1 OS=Homo sapiens GN=NOB1 PE=1 SV=1
NEST_HUMAN	Nestin OS=Homo sapiens GN=NES PE=1 SV=2
MYH9_HUMAN	Myosin-9 OS=Homo sapiens GN=MYH9 PE=1 SV=4
MTMR4_HUMAN	Myotubularin-related protein 4 OS=Homo sapiens GN=MTMR4 PE=1 SV=2
MTA2_HUMAN	Metastasis-associated protein MTA2 OS=Homo sapiens GN=MTA2 PE=1 SV=1

MSH2_HUMAN	DNA mismatch repair protein Msh2 OS=Homo sapiens GN=MSH2 PE=1 SV=1
MRE11_HUMAN	Double-strand break repair protein MRE11A OS=Homo sapiens GN=MRE11A PE=1 SV=3
MPCP_HUMAN	Phosphate carrier protein, mitochondrial OS=Homo sapiens GN=SLC25A3 PE=1 SV=2
MLH1_HUMAN	DNA mismatch repair protein Mlh1 OS=Homo sapiens GN=MLH1 PE=1 SV=1
MCM3_HUMAN	DNA replication licensing factor MCM3 OS=Homo sapiens GN=MCM3 PE=1 SV=3
MCA3_HUMAN	Eukaryotic translation elongation factor 1 epsilon-1 OS=Homo sapiens GN=EEF1E1 PE=1 SV=1
MAP1B_HUMAN	Microtubule-associated protein 1B OS=Homo sapiens GN=MAP1B PE=1 SV=2
MAGD1_HUMAN	Melanoma-associated antigen D1 OS=Homo sapiens GN=MAGED1 PE=1 SV=3
MACF1_HUMAN	Microtubule-actin cross-linking factor 1, isoforms 1/2/3/5 OS=Homo sapiens GN=MACF1 PE=1 SV=4
M2OM_HUMAN	Mitochondrial 2-oxoglutarate/malate carrier protein OS=Homo sapiens GN=SLC25A11 PE=1 SV=3
LRC59_HUMAN	Leucine-rich repeat-containing protein 59 OS=Homo sapiens GN=LRRC59 PE=1 SV=1
LRC58_HUMAN	Leucine-rich repeat-containing protein 58 OS=Homo sapiens GN=LRRC58 PE=1 SV=2
LIMS1_HUMAN	LIM and senescent cell antigen-like-containing domain protein 1 OS=Homo sapiens GN=LIMS1 PE=1 SV=4
LIMD1_HUMAN	LIM domain-containing protein 1 OS=Homo sapiens GN=LIMD1 PE=1 SV=1
LARP4_HUMAN	La-related protein 4 OS=Homo sapiens GN=LARP4 PE=1 SV=3
LAR4B_HUMAN	La-related protein 4B OS=Homo sapiens GN=LARP4B PE=1 SV=3
LAMA5_HUMAN	Laminin subunit alpha-5 OS=Homo sapiens GN=LAMA5 PE=1 SV=8
L2HDH_HUMAN	L-2-hydroxyglutarate dehydrogenase, mitochondrial OS=Homo sapiens GN=L2HGDH PE=1 SV=3
KITH_HUMAN	Thymidine kinase, cytosolic OS=Homo sapiens GN=TK1 PE=1 SV=2
KISHA_HUMAN	Protein kish-A OS=Homo sapiens GN=TMEM167A PE=1 SV=1
KHDR3_HUMAN	KH domain-containing, RNA-binding, signal transduction-associated protein 3 OS=Homo sapiens GN=KHDRBS3 PE=1 SV=1
KDM1A_HUMAN	Lysine-specific histone demethylase 1A OS=Homo sapiens GN=KDM1A PE=1 SV=2
KAP2_HUMAN	cAMP-dependent protein kinase type II-alpha regulatory subunit OS=Homo sapiens GN=PRKAR2A PE=1 SV=2
K2C1B_HUMAN	Keratin, type II cytoskeletal 1b OS=Homo sapiens GN=KRT77 PE=2 SV=3
K1C18_HUMAN	Keratin, type I cytoskeletal 18 OS=Homo sapiens GN=KRT18 PE=1 SV=2
IR3IP_HUMAN	Immediate early response 3-interacting protein 1 OS=Homo sapiens GN=IER3IP1 PE=1 SV=1
IMA5_HUMAN	Importin subunit alpha-5 OS=Homo sapiens GN=KPNA1 PE=1 SV=3
IMA1_HUMAN	Importin subunit alpha-1 OS=Homo sapiens GN=KPNA2 PE=1 SV=1

IGBP1_HUMAN	Immunoglobulin-binding protein 1 OS=Homo sapiens GN=IGBP1 PE=1 SV=1
IF4G1_HUMAN	Eukaryotic translation initiation factor 4 gamma 1 OS=Homo sapiens GN=EIF4G1 PE=1 SV=4
IDHP_HUMAN	Isocitrate dehydrogenase [NADP], mitochondrial OS=Homo sapiens GN=IDH2 PE=1 SV=2
IDH3G_HUMAN	Isocitrate dehydrogenase [NAD] subunit gamma, mitochondrial OS=Homo sapiens GN=IDH3G PE=1 SV=1
IDH3B_HUMAN	Isocitrate dehydrogenase [NAD] subunit beta, mitochondrial OS=Homo sapiens GN=IDH3B PE=1 SV=2
IDH3A_HUMAN	Isocitrate dehydrogenase [NAD] subunit alpha, mitochondrial OS=Homo sapiens GN=IDH3A PE=1 SV=1
HUWE1_HUMAN	E3 ubiquitin-protein ligase HUWE1 OS=Homo sapiens GN=HUWE1 PE=1 SV=3
HLTF_HUMAN	Helicase-like transcription factor OS=Homo sapiens GN=HLTF PE=1 SV=2
HELZ_HUMAN	Probable helicase with zinc finger domain OS=Homo sapiens GN=HELZ PE=1 SV=2
HEAT1_HUMAN	HEAT repeat-containing protein 1 OS=Homo sapiens GN=HEATR1 PE=1 SV=3
HCD2_HUMAN	3-hydroxyacyl-CoA dehydrogenase type-2 OS=Homo sapiens GN=HSD17B10 PE=1 SV=3
HACD3_HUMAN	Very-long-chain (3R)-3-hydroxyacyl-CoA dehydratase 3 OS=Homo sapiens GN=HACD3 PE=1 SV=2
GT251_HUMAN	Procollagen galactosyltransferase 1 OS=Homo sapiens GN=COLGALT1 PE=1 SV=1
GRWD1_HUMAN	Glutamate-rich WD repeat-containing protein 1 OS=Homo sapiens GN=GRWD1 PE=1 SV=1
GOGB1_HUMAN	Golgin subfamily B member 1 OS=Homo sapiens GN=GOLGB1 PE=1 SV=2
GBB1_HUMAN	Guanine nucleotide-binding protein G(I)/G(S)/G(T) subunit beta-1 OS=Homo sapiens GN=GNB1 PE=1 SV=3
GAPD1_HUMAN	GTPase-activating protein and VPS9 domain-containing protein 1 OS=Homo sapiens GN=GAPVD1 PE=1 SV=2
GANAB_HUMAN	Neutral alpha-glucosidase AB OS=Homo sapiens GN=GANAB PE=1 SV=3
G3BP1_HUMAN	Ras GTPase-activating protein-binding protein 1 OS=Homo sapiens GN=G3BP1 PE=1 SV=1
FLNB_HUMAN	Filamin-B OS=Homo sapiens GN=FLNB PE=1 SV=2
FKB10_HUMAN	Peptidyl-prolyl cis-trans isomerase FKBP10 OS=Homo sapiens GN=FKBP10 PE=1 SV=1
FBRL_HUMAN	rRNA 2'-O-methyltransferase fibrillarin OS=Homo sapiens GN=FBL PE=1 SV=2
ETFA_HUMAN	Electron transfer flavoprotein subunit alpha, mitochondrial OS=Homo sapiens GN=ETFA PE=1 SV=1
ESYT1_HUMAN	Extended synaptotagmin-1 OS=Homo sapiens GN=ESYT1 PE=1 SV=1
ELP1_HUMAN	Elongator complex protein 1 OS=Homo sapiens GN=IKBKAP PE=1 SV=3
ELAV2_HUMAN	ELAV-like protein 2 OS=Homo sapiens GN=ELAVL2 PE=1 SV=2
EIF3I_HUMAN	Eukaryotic translation initiation factor 3 subunit I OS=Homo sapiens GN=EIF3I PE=1 SV=1
EIF3F_HUMAN	Eukaryotic translation initiation factor 3 subunit F OS=Homo sapiens GN=EIF3F PE=1 SV=1

EIF3E_HUMAN	Eukaryotic translation initiation factor 3 subunit E OS=Homo sapiens GN=EIF3E PE=1 SV=1
EIF3B_HUMAN	Eukaryotic translation initiation factor 3 subunit B OS=Homo sapiens GN=EIF3B PE=1 SV=3
EIF3A_HUMAN	Eukaryotic translation initiation factor 3 subunit A OS=Homo sapiens GN=EIF3A PE=1 SV=1
EDC4_HUMAN	Enhancer of mRNA-decapping protein 4 OS=Homo sapiens GN=EDC4 PE=1 SV=1
ECHB_HUMAN	Trifunctional enzyme subunit beta, mitochondrial OS=Homo sapiens GN=HADHB PE=1 SV=3
DYHC2_HUMAN	Cytoplasmic dynein 2 heavy chain 1 OS=Homo sapiens GN=DYNC2H1 PE=1 SV=4
DX39A_HUMAN	ATP-dependent RNA helicase DDX39A OS=Homo sapiens GN=DDX39A PE=1 SV=2
DRG1_HUMAN	Developmentally-regulated GTP-binding protein 1 OS=Homo sapiens GN=DRG1 PE=1 SV=1
DPOD1_HUMAN	DNA polymerase delta catalytic subunit OS=Homo sapiens GN=POLD1 PE=1 SV=2
DNJB6_HUMAN	DnaJ homolog subfamily B member 6 OS=Homo sapiens GN=DNAJB6 PE=1 SV=2
DNJB1_HUMAN	DnaJ homolog subfamily B member 1 OS=Homo sapiens GN=DNAJB1 PE=1 SV=4
DHE3_HUMAN	Glutamate dehydrogenase 1, mitochondrial OS=Homo sapiens GN=GLUD1 PE=1 SV=2
DDB1_HUMAN	DNA damage-binding protein 1 OS=Homo sapiens GN=DDB1 PE=1 SV=1
CRNL1_HUMAN	Crooked neck-like protein 1 OS=Homo sapiens GN=CRNKL1 PE=1 SV=4
CPSF1_HUMAN	Cleavage and polyadenylation specificity factor subunit 1 OS=Homo sapiens GN=CPSF1 PE=1 SV=2
COX41_HUMAN	Cytochrome c oxidase subunit 4 isoform 1, mitochondrial OS=Homo sapiens GN=COX4I1 PE=1 SV=1
COPB_HUMAN	Coatomer subunit beta OS=Homo sapiens GN=COPB1 PE=1 SV=3
COPA_HUMAN	Coatomer subunit alpha OS=Homo sapiens GN=COPA PE=1 SV=2
COA7_HUMAN	Cytochrome c oxidase assembly factor 7 OS=Homo sapiens GN=COA7 PE=1 SV=2
CNOT4_HUMAN	CCR4-NOT transcription complex subunit 4 OS=Homo sapiens GN=CNOT4 PE=1 SV=3
CNOT1_HUMAN	CCR4-NOT transcription complex subunit 1 OS=Homo sapiens GN=CNOT1 PE=1 SV=2
CKAP5_HUMAN	Cytoskeleton-associated protein 5 OS=Homo sapiens GN=CKAP5 PE=1 SV=3
CHD4_HUMAN	Chromodomain-helicase-DNA-binding protein 4 OS=Homo sapiens GN=CHD4 PE=1 SV=2
CDN2A_HUMAN	Cyclin-dependent kinase inhibitor 2A OS=Homo sapiens GN=CDKN2A PE=1 SV=2
CDK4_HUMAN	Cyclin-dependent kinase 4 OS=Homo sapiens GN=CDK4 PE=1 SV=2
CBWD1_HUMAN	COBW domain-containing protein 1 OS=Homo sapiens GN=CBWD1 PE=1 SV=1
CARM1_HUMAN	Histone-arginine methyltransferase CARM1 OS=Homo sapiens GN=CARM1 PE=1 SV=3
CALX_HUMAN	Calnexin OS=Homo sapiens GN=CANX PE=1 SV=2

BRAT1_HUMAN	BRCA1-associated ATM activator 1 OS=Homo sapiens GN=BRAT1 PE=1 SV=2
BAG6_HUMAN	Large proline-rich protein BAG6 OS=Homo sapiens GN=BAG6 PE=1 SV=2
BAG2_HUMAN	BAG family molecular chaperone regulator 2 OS=Homo sapiens GN=BAG2 PE=1 SV=1
ATPO_HUMAN	ATP synthase subunit O, mitochondrial OS=Homo sapiens GN=ATP5O PE=1 SV=1
AT1A1_HUMAN	Sodium/potassium-transporting ATPase subunit alpha-1 OS=Homo sapiens GN=ATP1A1 PE=1 SV=1
ARF1_HUMAN	ADP-ribosylation factor 1 OS=Homo sapiens GN=ARF1 PE=1 SV=2
ARAF_HUMAN	Serine/threonine-protein kinase A-Raf OS=Homo sapiens GN=ARAF PE=1 SV=2
AKAP9_HUMAN	A-kinase anchor protein 9 OS=Homo sapiens GN=AKAP9 PE=1 SV=3
AGAL_HUMAN	Alpha-galactosidase A OS=Homo sapiens GN=GLA PE=1 SV=1
ACTZ_HUMAN	Alpha-centractin OS=Homo sapiens GN=ACTR1A PE=1 SV=1
ACL6A_HUMAN	Actin-like protein 6A OS=Homo sapiens GN=ACTL6A PE=1 SV=1
ABCF3_HUMAN	ATP-binding cassette sub-family F member 3 OS=Homo sapiens GN=ABCF3 PE=1 SV=2
ABCF2_HUMAN	ATP-binding cassette sub-family F member 2 OS=Homo sapiens GN=ABCF2 PE=1 SV=2
ABCE1_HUMAN	ATP-binding cassette sub-family E member 1 OS=Homo sapiens GN=ABCE1 PE=1 SV=1
AAAT_HUMAN	Neutral amino acid transporter B(0) OS=Homo sapiens GN=SLC1A5 PE=1 SV=2
2AAA_HUMAN	Serine/threonine-protein phosphatase 2A 65 kDa regulatory subunit A alpha isoform OS=Homo sapiens GN=PPP2R1A PE=1 SV=4

Note: Proteins highlighted in red are previously identified by the N-terminomics[17].

Supplementary Data File 2: 63 proteins uniquely identified in both the RHBDL4(S144Dap) and RHBDL4(S144A) samples.

Protein ID	Gene name	Protein name	Localization	Note
Q15084	PDIA6	Protein disulfide-isomerase A6	ER	
P13667	PDIA4	Protein disulfide-isomerase A4	ER	
P13674	P4HA1	Prolyl 4-hydroxylase α1	ER	
Q8N1F7	NUP93	Nucleoporin Nup93	Nuclear pore complex	
P50570	DNM2	Dynamin-2	cytoskeleton	
Q99615	DNAJC7	DnaJ homolog subfamily C7	Cytosol, Nucleus	
Q08426	EHHADH	Peroxisomal bifunctional enzyme	Peroxisome	
Q15293	ECN1	Reticulocalbin-1	ER	
Q07065	CKAP4	Cytoskeleton-associated protein 4	ER	Transmembrane
O94901	SUN1	SUN domain-containing protein 1	Nuclear inner membrane	Transmembrane
Q02218	OGDH	2-oxoglutarate dehydrogenase	Mitochondrion	
Q8NE01	CNNM3	Metal transporter CNNM3	Plasma membrane	Transmembrane
P48449	LSS	Lanosterol synthase	ER	
Q9UKM7	MAN1B1	ER mannosidase 1	ER	Transmembrane
Q5JTZ9	AARS2	Alanine-tRNA ligase	Mitochondrion	
P18850	ATF6	c-AMP-dependent transcription factor ATF-6α	ER	Transmembrane
Q8IXB1	DNAJC10	DnaJ homolog subfamily C10	ER	
Q15436	SEC23A	Protein transport protein Sec23A	Cytosol, ER	
Q9H9S5	FKRP	Fukutin-related protein	ER, Golgi	
P11413	G6PD	Glucose-6-phosphate 1-dehydrogenase	Cytosol	
O95292	VAPB	Vesicle-associated membrane protein-associated protein	ER	Transmembrane
Q14257	RCN2	Reticulocalbin-2	ER	
Q9H0V1	TMEM168	Transmembrane protein 168	membrane	
Q96S52	PIGS	GPI transamidase component PIG-S	ER	Transmembrane
Q9NYJ8	TAB2	TAK1-binding protein	Cytosol	

Q9BY77	POLDIP3	Polymerase δ-interacting protein 3	Nucleus	
P30101	PDIA3	Protein disulfide-isomerase A3	ER	
Q96AY3	FKBP10	Peptidyl-prolyl cis-trans isomerase FKBP10	ER	
Q12797	ASPH	Aspartyl-asparaginyl β-hydroxylase	ER	Transmembrane
Q14435	GALNT3	Polypeptide N-acetylgalactosaminyltransferase 3	Golgi	Transmembrane
Q9UM54	MYO6	Unconventional myosin-VI	Cytosol	
O00159	MYO1C	Unconventional myosin-Ic	Nucleus, Cytosol	
Q8IZH2	XRN1	5'-5' exoribonuclease 1	Cytosol	
P49790	NUP153	Nuclear pore complex protein Nup153	Nuclear pore complex	
Q96QK1	VPS35	Vacuolar protein sorting-associated protein 35	Endosome	
P04181	OAT	Ornithine aminotransferase	Mitochondrion	
Q2NL82	TSR1	Pre-rRNA-processing protein TSR1 homolog	Nucleus	
O75534	CSDE1	Cold shock domain-containing protein E1	Cytosol	
P15104	GLUL	Glutamine synthetase	Cytosol	
Q5H8A4	PIGG	GPI ethanolamine phosphate transferase 2	ER	Transmembrane
Q8WZA1	POMGNT1	Protein O-linked-mannose β-1,2-N-acetylglucosaminyltransferase 1	Golgi	
Q13492	PICALM	Phosphatidylinositol-binding clathrin assembly protein	Cytosol	
P23378	GLDC	Glycine dehydrogenase	Mitochondrion	
O75521	ECI2	Enoyl-CoA δ isomerase 2	Mitochondrion	
O00411	POLRMT	DNA-directed RNA polymerase	Mitochondrion	
Q5JTH9	RRP12	RRP12-like protein	Nuclear inner membrane	Transmembrane
Q5T1Q4	SLC35F1	Solute carrier family 35 member F1	Membrane	Transmembrane
Q969M3	YIPF5	Protein YIPF5	ER, Golgi	Transmembrane
Q9Y285	FARSA	Phenylalanine-tRNA ligase α subunit	Cytosol	
Q86X29	LSR	Lipolysis-stimulated lipoprotein receptor	Plasma membrane	Transmembrane
O14617	AP3D1	AP-3 complex subunit δ-1	Golgi	
O14980	XPO1	Exportin-1	Nucleus, Cytosol	
Q68DH5	LMBRD2	G-protein coupled receptor-associated protein LMBRD2	Plasma membrane	Transmembrane

P17812	CTPS1	CTP synthase 1	Cytosol	
Q9UHI6	DDX20	Probable ATP-dependent RNA helicase DDX20	Nucleus, Cytosol	
Q86UL3	GPAT4	Glycerol-3-phosphate acytransferase 4	ER	Transmembrane
Q9BYD6	MRPL1	39S ribosomal protein L1	Mitochondrion	
Q9H2M9	RAB3GAP2	Rab3 GTPase-activating protein non-catalytic subunit	Cytosol	
Q9Y2A7	NCKAP1	Nck-associated protein 1	Plasma membrane	Transmembrane
Q92903	CDS1	Phosphatidate cytidylyltransferase 1	ER	Transmembrane
Q15334	LLGL1	Lethal giant larvae protein homolog 1	Cytoskeleton	
P02545	LMNA	Prelamin-A/C	Nucleus	
Q8NI60	COQ8A	Atypical kinase COQ8A	Mitochondrion	

Supplementary references

1 Fleig, L. *et al.* Ubiquitin-dependent intramembrane rhomboid protease promotes ERAD of membrane proteins. *Mol Cell* **47**, 558-569, doi:10.1016/j.molcel.2012.06.008 (2012).

2 Knopf, J. D. *et al.* Intramembrane protease RHBDL4 cleaves oligosaccharyltransferase subunits to target them for ER-associated degradation. *J Cell Sci*, doi:10.1242/jcs.243790 (2020).

3 Paschkowsky, S., Hamze, M., Oestereich, F. & Munter, L. M. Alternative Processing of the Amyloid Precursor Protein Family by Rhomboid Protease RHBDL4. *J Biol Chem* **291**, 21903-21912, doi:10.1074/jbc.M116.753582 (2016).

4 Wunderle, L. *et al.* Rhomboid intramembrane protease RHBDL4 triggers ER-export and non-canonical secretion of membrane-anchored TGFalpha. *Sci Rep* **6**, 27342, doi:10.1038/srep27342 (2016).

5 Chen, Q. *et al.* Inverting the Topology of a Transmembrane Protein by Regulating the Translocation of the First Transmembrane Helix. *Mol Cell* **63**, 567-578, doi:10.1016/j.molcel.2016.06.032 (2016).

6 Denard, B., Han, S., Kim, J., Ross, E. M. & Ye, J. Regulating G protein-coupled receptors by topological inversion. *Elife* **8**, doi:10.7554/eLife.40234 (2019).

7 Lim, J. J. *et al.* Structural insights into the interaction of p97 N-terminus domain and VBM in rhomboid protease, RHBDL4. *Biochem J* **473**, 2863-2880, doi:10.1042/BCJ20160237 (2016).

8 Lemberg, M. K. & Freeman, M. Functional and evolutionary implications of enhanced genomic analysis of rhomboid intramembrane proteases. *Genome Res* **17**, 1634-1646, doi:10.1101/gr.6425307 (2007).

9 Freeman, M. The rhomboid-like superfamily: molecular mechanisms and biological roles. *Annu Rev Cell Dev Biol* **30**, 235-254, doi:10.1146/annurev-cellbio-100913-012944 (2014).

10 Brown, M. S., Ye, J., Rawson, R. B. & Goldstein, J. L. Regulated intramembrane proteolysis: a control mechanism conserved from bacteria to humans. *Cell* **100**, 391-398, doi:10.1016/s0092-8674(00)80675-3 (2000).

11 Johnson, N. *et al.* Quantitative proteomics screen identifies a substrate repertoire of rhomboid protease RHBDL2 in human cells and implicates it in epithelial homeostasis. *Sci Rep* **7**, 7283, doi:10.1038/s41598-017-07556-3 (2017).

12 Kühnle, N. *et al.* Intramembrane protease RHBDL4 interacts with erlin complex to target unstable soluble proteins for degradation. 848754, doi:10.1101/848754 %J bioRxiv (2019).

13 Dickey, S. W., Baker, R. P., Cho, S. & Urban, S. Proteolysis inside the membrane is a rate-governed reaction not driven by substrate affinity. *Cell* **155**, 1270-1281, doi:10.1016/j.cell.2013.10.053 (2013).

14 Wu, Z. *et al.* Structural analysis of a rhomboid family intramembrane protease reveals a gating mechanism for substrate entry. *Nat Struct Mol Biol* **13**, 1084-1091, doi:10.1038/nsmb1179 (2006).

15 Huguenin-Dezot, N. *et al.* Trapping biosynthetic acyl-enzyme intermediates with encoded 2,3-diaminopropionic acid. *Nature* **565**, 112-117, doi:10.1038/s41586-018-0781-z (2019).

16 Genereux, J. C. *et al.* Unfolded protein response-induced ERdj3 secretion links ER stress to extracellular proteostasis. *EMBO J* **34**, 4-19, doi:10.15252/embj.201488896 (2015).
17 Vande Walle, L. *et al.* Proteome-wide Identification of HtrA2/Omi Substrates. *J Proteome Res* **6**, 1006-1015, doi:10.1021/pr060510d (2007).
