# Supplementary material for: Mechanism-based traps enable protease and hydrolase substrate discovery
Source: Nature. 2022 Feb 16;602(7898):701–7. doi: 10.1038/s41586-022-04414-9 (PMC8866121; doi:10.1038/s41586-022-04414-9)
Supplement: Supplementary file 2 — Reporting Summary [file 41586_2022_4414_MOESM2_ESM.pdf]

nature portfolio

Corresponding author(s): Shan Tang, Jason W. Chin

Last updated by author(s): Dec 15, 2021

Reporting Summary

Nature Portfolio wishes to improve the reproducibility of the work that we publish. This form provides structure for consistency and transparency in reporting. For further information on Nature Portfolio policies, see our Editorial Policies and the Editorial Policy Checklist.

Statistics

For all statistical analyses, confirm that the following items are present in the figure legend, table legend, main text, or Methods section.

n/a	Confirmed	
☐	☒	The exact sample size (n) for each experimental group/condition, given as a discrete number and unit of measurement
☐	☒	A statement on whether measurements were taken from distinct samples or whether the same sample was measured repeatedly
☐	☒	The statistical test(s) used AND whether they are one- or two-sided<br>*Only common tests should be described solely by name; describe more complex techniques in the Methods section.*
☒	☐	A description of all covariates tested
☒	☐	A description of any assumptions or corrections, such as tests of normality and adjustment for multiple comparisons
☐	☒	A full description of the statistical parameters including central tendency (e.g. means) or other basic estimates (e.g. regression coefficient) AND variation (e.g. standard deviation) or associated estimates of uncertainty (e.g. confidence intervals)
☐	☒	For null hypothesis testing, the test statistic (e.g. F, t, r) with confidence intervals, effect sizes, degrees of freedom and P value noted<br>*Give P values as exact values whenever suitable.*
☒	☐	For Bayesian analysis, information on the choice of priors and Markov chain Monte Carlo settings
☒	☐	For hierarchical and complex designs, identification of the appropriate level for tests and full reporting of outcomes
☒	☐	Estimates of effect sizes (e.g. Cohen's d, Pearson's r), indicating how they were calculated

Our web collection on statistics for biologists contains articles on many of the points above.

Software and code

Policy information about availability of computer code

Data collection	Odyssey CLx imaging system (Li-Cor); MS Chemstation (Rev.C.01.06[61]); MARS Data Analysis Software (version 3.20 R2); XIA2 (version 0.7.90), DIALS (version 3.1.3), scaled using Aimless and Refmac (version 5.8.0258) in the CCP4 suite (version 7.0.078) of programs for crystal data processing
Data analysis	Image Studio Lite (version 5.2.5) for western blot analysis; Mascot Search Engine Program (version 2.4) and Scaffold Proteome Software (version 4) for protein identification (Venn Diagrams); MaxQuant Software (version 1.6.3.4) and Perseus (version 1.6.2.3) for LFQ analysis (Volcano Plots); FCS Express Flow Cytometry software (version 7) for FACS data analysis; Fiji (ImageJ2) for IF analysis; GraphPad Prism (version 8) for graphs; Refmac (version 5.8.0258) and COOT (0.8.9.2) for structure refinement and manual model building; Pymol (version 1.0r2) for protein structural visualization; ProteoWizard (version 3.0.11252) for converting LC-MS/MS RAW files to mzML format; Custom Python (version 3.8.1) scripts with the pyOpenMS package (version 2.4.0) for MS data preparation and processing. The code used for RBBP9 substrate identification by LC-MS/MS analysis is available at https://doi.org/10.5281/zenodo.5768340.

For manuscripts utilizing custom algorithms or software that are central to the research but not yet described in published literature, software must be made available to editors and reviewers. We strongly encourage code deposition in a community repository (e.g. GitHub). See the Nature Portfolio guidelines for submitting code & software for further information.

Data

Policy information about availability of data

All manuscripts must include a data availability statement. This statement should provide the following information, where applicable:

- Accession codes, unique identifiers, or web links for publicly available datasets
- A description of any restrictions on data availability
- For clinical datasets or third party data, please ensure that the statement adheres to our policy

The structure of RBBP9 in complex with Phe is available in the Protein Data Bank under accession code 7OEX. The mass spectrometry proteomics data have been deposited to the ProteomeXchange Consortium via the PRIDE partner repository with the accession number PDX030381. All other datasets and materials generated or analyzed in this study are available from the corresponding authors upon reasonable request. The data used to analyze serine and cysteine proteases clans were downloaded from the MEROPS database (https://www.ebi.ac.uk/merops/)

Field-specific reporting

Please select the one below that is the best fit for your research. If you are not sure, read the appropriate sections before making your selection.

☒ Life sciences ☐ Behavioural & social sciences ☐ Ecological, evolutionary & environmental sciences

For a reference copy of the document with all sections, see nature.com/documents/nr-reporting-summary-flat.pdf

Life sciences study design

All studies must disclose on these points even when the disclosure is negative.

Sample size	No sample size calculations were performed. These are biochemical experiments, not animal experiments, and there are therefore no individuals to sample. Sample size in not a relevant parameter, but the number of replicates is relevant. The number of replicates is indicated in the relevant figure legend.
Data exclusions	No data was excluded
Replication	Three replicates were performed for protein identification by LC-MS/MS; Two replicates were performed to obtain the entire masses. All attempts at replicates were successful. Three or two replicates (annotated in the figure legends) were performed for immunoblotting analysis. All immunobltoting analysis were reproducible. Two replicates were performed for fluorescence-based aminopeptidase activity analysis. All fluorescent measurements were reproducible. All experiments were performed in the number of replicates indicated in the figure legends. All replicates were successful.
Randomization	These are biochemical experiments, where different components need to be added to different reactions. A single investigator needs to perform defined distinct and skilled operations on different samples to make the experiment meaningful and therefore randomization does not make sense.
Blinding	These are biochemical experiments, where different components need to be added to different reactions. A single investigator needs to perform defined distinct and skilled operations on different samples to make the experiment meaningful and therefore blinding does not make sense.

Reporting for specific materials, systems and methods

We require information from authors about some types of materials, experimental systems and methods used in many studies. Here, indicate whether each material, system or method listed is relevant to your study. If you are not sure if a list item applies to your research, read the appropriate section before selecting a response.

Materials & experimental systems

n/a	Involved in the study	
☐	☒	Antibodies
☐	☒	Eukaryotic cell lines
☒	☐	Palaeontology and archaeology
☒	☐	Animals and other organisms
☒	☐	Human research participants
☒	☐	Clinical data
☒	☐	Dual use research of concern

Methods

n/a	Involved in the study	
☒	☐	ChIP-seq
☐	☒	Flow cytometry
☒	☐	MRI-based neuroimaging

Antibodies

Antibodies used

Anti-Strep (ab76949); Anti-Calreticulin (ab92516); Anti-PDIA6 (ab154820); Anti-KDEL (ab176333); Anti-POLR2A (ab76123); Anti-POLR2B (ab228933); Anti-DYNC1H1 (ab245554); Anti-GCN1 (ab86139); Anti-SEC16A (ab70722); Anti-RANBP2 (ab2938); Anti-FASN (ab128870); Anti-BIRC6 (ab19609); Anti-CLTC (ab172958); Anti-PLEC (ab32528); Anti-POLR2C (ab182150); Anti-RBM4 (ab251923); Anti-TRIP13 (ab128171); Anti-EPRS (ab31531); Anti-DNAJA1 (ab126774); Anti-RBM14 (ab228692); Anti-RACK1 (ab129084); Anti-IRS4 (ab52622); Anti-ATP5F1A (ab176569); Anti-OAT (ab137679), Anti-TUFM (ab173300) and Anti-RBBP9 (ab157202) were from abcam. Anti-GFP (2956(D5.1)); Anti-HA (3724(C29F4); Anti-HA (2367(6E2)); Anti-GAPDH (2118(14C10)) and Anti-Tubulin (86298(D3U1W)) were from Cell Signaling. Anti-V5 (R960-25) was from Thermo Fisher. Anti-FLAG (F3165) and Anti-CCDC47 (HPA029674) were from Merck. Anti-FLAG (AHP1074GA) was from Bio-Rad. Anti-MDN1 (A304-739A-T) and Anti-WDCP (A303-337-T) were from Bethyl Laboratories. Anti-BiP (GTX113340); Anti-MCM7 (GTX110278) and Anti-FAM120A (GTX120824) were from GeneTex. Anti-MTMR3 (H00008897-B01P) was from Novus Biologicals. Anti-RHBDL4 (20869-1-AP); Anti-Calnexin (10427-2-AP); Anti-LaminB1(12987-1-AP), Anti-Transferrin (66171-1-Ig) and Anti-SNRNP200 (23875-1-AP) were from Proteintech. Anti-DSP (MAB9080) and Anti-Ub (MAB8595) were from R&D Systems. Anti-RPS3 (A2533) was from ABclonal. Anti-GAPDH (TA802519) was from Origene. Goat anti-Mouse (925-68070; 925-32210) secondary antibodies and Goat anti-Rabbit (925-68071; 926-32211) secondary antibodies were from Li-Cor.

Validation

Validations are based on the data from the manufacturers.
Anti-Strep (ab76949) was validated by detecting recombinant proteins with strep-tag in cell lysate. https://www.abcam.com/strep-tag-ii-antibody-ab76949.html
Anti-Calreticulin (ab92516) was knockout validated in HAP1 cell line. https://www.abcam.com/calreticulin-antibody-epr3924-er-marker-ab92516.html
Anti-PDIA6 (ab154820): https://www.abcam.com/pdia6-antibody-epr10132b-ab154820.html
Anti-KDEL (ab176333): https://www.abcam.com/kdel-antibody-epr12668-ab176333.html
Anti-POLR2A (ab76123): https://www.abcam.com/rna-polymerase-ii-rpb1-antibody-epr1509y-chip-grade-ab76123.html
Anti-POLR2B (ab228933): https://www.abcam.com/rpb2-antibody-c-terminal-ab228933.html
Anti-DYNC1H1 (ab245554): https://www.abcam.com/dync1h1-antibody-ab245554.html
Anti-GCN1 (ab86139): https://www.abcam.com/gcn1-antibody-ab86139.html
Anti-SEC16A (ab70722): https://www.abcam.com/sec16asec16-antibody-ab70722.html
Anti-RANBP2 (ab2938): https://www.abcam.com/ranbp2-antibody-ab2938.html
Anti-FASN (ab128870) was knockout validated in HAP1 cell line. https://www.abcam.com/fatty-acid-synthase-antibody-epr7466-ab128870.html?productWallTab=ShowAll
Anti-BIRC6 (ab19609): https://www.abcam.com/birc6apollon-antibody-ab19609.html?productWallTab=ShowAll
Anti-CLTC (ab172958): https://www.abcam.com/clathrin-heavy-chain-antibody-epr12235b-ab172958.html
Anti-PLEC (ab32528) was knockout validated in U2OS cell line. https://www.abcam.com/plectin-antibody-e398p-ab32528.html?productWallTab=ShowAll
Anti-POLR2C (ab182150): https://www.abcam.com/rpb3-antibody-epr13294b-ab182150.html
Anti-RBM4 (ab251923): https://www.abcam.com/rbm4-antibody-ab251923.html
Anti-TRIP13 (ab128171): https://www.abcam.com/trip13pch2-antibody-ab128171.html?productWallTab=ShowAll
Anti-EPRS (ab31531): https://www.abcam.com/glutamyl-prolyl-trna-synthetasepars-antibody-ab31531.html?productWallTab=ShowAll
Anti-DNAJA1 (ab126774) was knockout validated in HEK293T cell line. https://www.abcam.com/dnaja1-antibody-epr7248-ab126774.html
Anti-RBM14 (ab228692): https://www.abcam.com/rbm14-antibody-n-terminal-ab228692.html
Anti-RACK1 (ab129084): https://www.abcam.com/rack1-antibody-epr7388-ab129084.html
Anti-IRS4 (ab52622): https://www.abcam.com/irs4-antibody-ep907y-ab52622.html
Anti-ATP5F1A (ab176569): https://www.abcam.com/atp5a-antibody-epr13030b-ab176569.html?productWallTab=ShowAll
Anti-OAT (ab137679): https://www.abcam.com/ornithine-aminotransferase-antibody-ab137679.html
Anti-TUFM (ab173300): https://www.abcam.com/tufm-antibody-epr12797b-ab173300.html
Anti-GFP (D5.1, 2956) was validated by detecting recombinant GFP in cell lysate. https://www.cellsignal.co.uk//products/primary-antibodies/gfp-d5-1-rabbit-mab/2956
Anti-HA (3724(C29F4) was validated by detecting recombinant proteins with HA-tag in cell lysate. https://www.cellsignal.co.uk/products/primary-antibodies/ha-tag-c29f4-rabbit-mab/3724?site-search-type=Products&N=4294956287&Ntt=3724s&fromPage=plp&_requestid=1510669
Anti-HA (2367(6E2)): https://www.cellsignal.com/products/primary-antibodies/ha-tag-6e2-mouse-mab/2367
Anti-GAPDH (2118(14C10)): https://www.cellsignal.com/products/primary-antibodies/gapdh-14c10-rabbit-mab/2118
Anti-GAPDH (TA802519): https://www.origene.com/catalog/antibodies/primary-antibodies/ta802519/gapdh-mouse-monoclonal-antibody-clone-id-oti2d9
Anti-Tubulin (86298(D3U1W)): https://www.cellsignal.co.uk/products/primary-antibodies/b-tubulin-d3u1w-mouse-mab/86298
Anti-V5 (R960-25) was validated by detecting V5-tagged recombinant proteins in cell lysate. https://www.thermofisher.com/antibody/product/V5-Tag-Antibody-Monoclonal/R960-25
Anti-FLAG (F3165) was validated by detecting FLAG-tagged recombinant proteins in cell lysate. https://www.sigmaaldrich.com/GB/en/product/sigma/f3165?context=product
Anti-FLAG (AHP1074GA) was validated by detecting recombinant proteins with FLAG-tag in cell lysate. https://www.bio-rad-antibodies.com/polyclonal/synthetic-peptide-dykddddk-tag-antibody-ahp1074.html?f=purified
Anti-CCDC47 (HPA029674): https://www.sigmaaldrich.com/GB/en/product/sigma/hpa029674
Anti-MDN1 (A304-739A-T): https://www.bethyl.com/product/A304-739A/Midasin+Antibody
Anti-WDCP (A303-337-T): https://www.bethyl.com/product/A303-337A?referrer=search#
Anti-BiP (GTX113340): https://www.genetex.com/Product/Detail/Grp78-antibody/GTX113340#datasheet
Anti-MCM7 (GTX110278): https://www.genetex.com/Product/Detail/MCM7-antibody-N2C2-Internal/GTX110278
Anti-FAM120A (GTX120824): https://www.genetex.com/Product/Detail/FAM120A-antibody/GTX120824
Anti-MTMR3 (H00008897-B01P): validated by detection of MTMR3 in transfected 273T cell line. https://www.novusbio.com/products/mtmr3-antibody_h00008897-b01p?utm_source=citeab&utm_medium=referral&utm_campaign=product&utm_term=primaryantibodies

Anti-RHBDL4 (20869-1-AP) was knockout validated in HCT116 cell line in the current paper. The RHBDL4 positive bands were detected in HEK293 cell line and mouse kidney and testis tissues. https://www.ptgcn.com/products/RHBDD1-Antibody-20869-1-AP.htm
Anti-Calnexin (10427-2-AP): https://www.ptglab.com/products/CANX-Antibody-10427-2-AP.htm
Anti-LaminB1(12987-1-AP): https://www.ptgcn.com/products/LMNB1-Antibody-12987-1-AP.htm
Anti-SNRNP200 (23875-1-AP): https://www.ptgcn.com/products/SNRNP200-Antibody-23875-1-AP.htm
Anti-DSP (MAB9080): https://www.rndsystems.com/cn/products/human-desmoplakin-antibody-824038_mab9080
Anti-RPS3 (A2533): https://abclonal.com/catalog-antibodies/RPS3RabbitpAb/A2533
Goat anti-Mouse (925-68070): https://www.licor.com/bio/reagents/irdye-680rd-goat-anti-mouse-igg-secondary-antibody
Goat anti-Mouse (925-32210): https://www.licor.com/bio/reagents/irdye-800cw-goat-anti-mouse-igg-secondary-antibody
Goat anti-Rabbit (925-68071): https://www.licor.com/bio/reagents/irdye-680rd-goat-anti-rabbit-igg-secondary-antibody
Goat anti-Rabbit (926-32211): https://www.licor.com/bio/reagents/irdye-800cw-goat-anti-rabbit-igg-secondary-antibody
Anti-transferrin (66171-1-Ig): https://www.ptgcn.com/products/TF-Antibody-66171-1-Ig.htm
Anti-Ub (MAB8595): https://www.rndsystems.com/cn/products/human-ubiquitin-antibody-1002a_mab8595
Anti-RBBP9 (ab157202): https://www.abcam.com/bog-antibody-epr9905-ab157202.html

Eukaryotic cell lines

Policy information about cell lines

Cell line source(s)	HEK293T cells were purchased from European Collection of Cell Cultures (ECACC).Expi293 cells (A14527) were purchased from Thermo Fisher. HCT116 cells were purchased from American Type Culture Collection (ATCC).
Authentication	HEK293T cell line was authenticated by ECACC, Expi293 cell line was authenticated by Thermo Fisher, and HCT116 cell line was authenticated by ATCC using STR DNA profiling analysis to rule-out intra- and inter-species contamination.
Mycoplasma contamination	All cell lines tested negative for Mycoplasma contamination.
Commonly misidentified lines (See ICLAC register)	None

Flow Cytometry

Plots

Confirm that:

☒ The axis labels state the marker and fluorochrome used (e.g. CD4-FITC).

☒ The axis scales are clearly visible. Include numbers along axes only for bottom left plot of group (a 'group' is an analysis of identical markers).

☒ All plots are contour plots with outliers or pseudocolor plots.

☒ A numerical value for number of cells or percentage (with statistics) is provided.

Methodology

Sample preparation	48 h after transfection, cells in 24-well plates were washed with PBS, detached by trypsin/EDTA solution, and resuspended in the growth medium. Cells were pelleted and resuspended in PBS supplemented with 3% FBS.
Instrument	Becton Dickinson LSRFortessa (407 nm violet laser (V-450) for BFP excitation, 488 nm blue laser (B-525) for eGFP excitation, 561 nm yellow-green laser (YG-610) for mCherry excitation).
Software	FCS Express 7 software (De Novo software)
Cell population abundance	20,000 intact cells (gated by from scatter and side scatter) were analyzed for each sample.
Gating strategy	The front scatter and side scatter were used to identify intact cells. Non-transfected cells, cells only transfected with mCherry or eGFP plasmid and BFP positive cells were used to define the gate.

☒ Tick this box to confirm that a figure exemplifying the gating strategy is provided in the Supplementary Information.
